# Prior Knowledge for Predictive Modeling: The Case of Acute Aquatic Toxicity

**DOI:** 10.1021/acs.jcim.1c01079

**Published:** 2022-08-23

**Authors:** Gulnara Shavalieva, Stavros Papadokonstantakis, Gregory Peters

**Affiliations:** † Department of Space, Earth and Environment, Division of Energy Technology, Chalmers University of Technology, SE-412 96 Gothenburg, Sweden; ‡ Institute of Chemical, Environmental and Bioscience Engineering, TU Wien, Getreidemarkt 9, 1060 Vienna, Austria; § Department of Technology Management and Economics, Chalmers University of Technology, SE-411 33 Gothenburg, Sweden

## Abstract

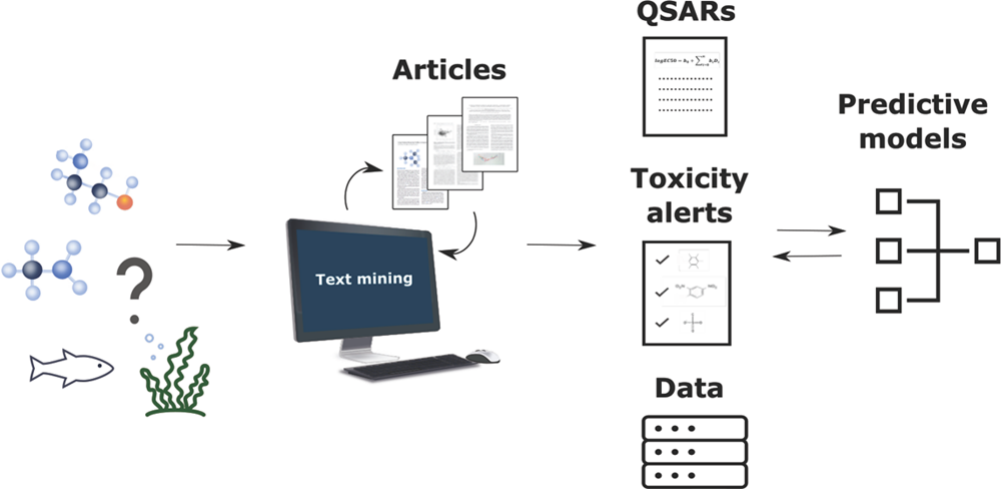

Early assessment of the potential impact of chemicals on health and the environment requires toxicological properties of the molecules. Predictive modeling is often used to estimate the property values *in silico* from pre-existing experimental data, which is often scarce and uncertain. One of the ways to advance the predictive modeling procedure might be the use of knowledge existing in the field. Scientific publications contain a vast amount of knowledge. However, the amount of manual work required to process the enormous volumes of information gathered in scientific articles might hinder its utilization. This work explores the opportunity of semiautomated knowledge extraction from scientific papers and investigates a few potential ways of its use for predictive modeling. The knowledge extraction and predictive modeling are applied to the field of acute aquatic toxicity. Acute aquatic toxicity is an important parameter of the safety assessment of chemicals. The extensive amount of diverse information existing in the field makes acute aquatic toxicity an attractive area for investigation of knowledge use for predictive modeling. The work demonstrates that the knowledge collection and classification procedure could be useful in hybrid modeling studies concerning the model and predictor selection, addressing data gaps, and evaluation of models’ performance.

## Introduction

Environmental hazard, risk, and life-cycle (LCA) assessments of existing and newly developed chemicals for various industrial processes are highly dependent on the availability of chemical property data, which are often challenging to obtain. For instance, data on toxicological properties have been traditionally obtained through *in vivo* testing, resulting in the death of many animals and significant financial expenses.^1^ From early on, it was realized that the need for experimental testing could be reduced by applying *in silico* methods assisting in the prediction of chemical property data required for the chemicals’ safety assessment. *In silico* or nontesting methods for obtaining chemical property data include quantitative structure–activity relationships (QSARs), pharmacophores, and molecular modeling and data analysis tools, including machine learning (ML), data mining (DM) algorithms, and network analysis.^2^ The methods are constantly improved, and new tools are developed to enhance their performance and reliability.

*In silico* approaches are often used for prescreening a vast number of chemical alternatives to select potentially better options before proceeding with more rigorous and more resource intensive approaches, typically including experimental evaluation. They are constantly gaining ground over human expertise-based brainstorming, especially in early phases of chemical process and product design. The information on the potential impact on health and the environment caused by the production and use of chemicals, including the development of new compounds, has been shown to facilitate the design of greener alternatives from early on, before their synthesis, commercialization, and use. For instance, *in silico* methods are largely used in the automated computer-aided molecular design (CAMD) in the form of predictive models of physicochemical properties based on molecular structure. To this end, property prediction techniques that describe broad classes of compounds are desired to improve computational efficiency (i.e., to avoid use of segregated information and look-up tables), often in the cost of more accurate predictions. This is however generally accepted in these early design phases, given the complexity and broadness of the task (e.g., often many thousands of chemical structures are screened). Clearly, when the CAMD algorithm compares some hundreds or thousands of various molecular alternatives, the relative information on properties is of greater importance than the exact values. Thus, the accuracy of the methods must be sufficient to result in a meaningful final list of the candidate molecules.^3^ While the accuracy of the thermodynamic property prediction models (e.g., boiling point, viscosity, heat capacity) is typically high, the predictions of the sustainability-related properties are subjected to a lower accuracy due to the lack of models, often as a result of lack of data required to construct the model.^4^ However, despite the uncertainties introduced by the prediction models, it is beneficial to incorporate sustainability related indices during CAMD to widen the multicriteria nature of the screening,^4^ rather than completely ignore these less accurate sustainability related property predictions only to perform rigorous sustainability assessment (e.g., based on more solid experimental evidence) in later phases of design for very few selected compounds. Thus, researchers apply the models but find ways to account for the prediction errors by, for example, relaxing the property constraints, running a sensitivity,^5^ uncertainty,^6^ or reliability^7^ analysis. The most common indices incorporated into CAMD are health, safety, and environmental indicators,^7−11^ computed using such properties like acute oral toxicity and permissible exposure limits, flammability and explosiveness, and aquatic toxicity and bioconcentration, respectively. A limited number of studies integrated also LCA indices. For example, Weis and Visco (2010)^11^ and Heintz et al. (2014)^12^ integrated a single LCA score computed by quantitative–structure property relationships (QSPRs) constructed using the data on 46 frequently used solvents. Papadopoulos et al. (2020)^4^ applied a ML-based FineChem model with accuracy 20–40% to estimate the LCA values from fragments constituting the molecules. It should be clear that all these attempts to incorporate sustainability related properties into CAMD do not have the same rigor as, for instance, the one required in occupational health and safety reports or hazard and operability studies where the domain of interest is orders of magnitude smaller with respect to the number of chemicals.

The ML models show better performance and increased reliability in predicting the property values of chemicals when provided with a larger amount and better-quality training data. However, such data are often limited, especially for the newly designed chemical structures. One way to deal with insufficient training data is the development of models integrating data and scientific knowledge already existing in a certain field.^13−15^ Such models use both data and field-specific information, also called prior knowledge (PK) (e.g., in the form of certain generalizations and rules from a relevant field). The integration of prior knowledge improves robustness^15^ and interpretability of the model outputs.^14^ The approach has been successfully applied for image recognition, weather and climate modeling, medicine, bioinformatics, etc. For instance, Diligenti et al. (2017)^16^ have demonstrated an accuracy increase of a state-of-the-art deep neural network applied for image classification with the integration of prior knowledge. Faghmous and Kumar (2014)^17^ and Kashinath et al. (2021)^18^ have highlighted the importance of introducing the scientific theory and first-principles constraints to avoid dubious findings made by models built on large volumes of climate data. Culos et al. (2020)^19^ reported improved predictions for clinically relevant outcomes when immunological knowledge was incorporated into predictive models. Xuan et al. (2019)^20^ have integrated knowledge about drugs and diseases and sparse characteristics of drug-disease associations into predictive models to capture drug-related disease indications. The use of prior knowledge has also been shown to benefit the performance of chemical property prediction models. Palomba et al. (2012)^21^ have developed quantitative structure–property relationship (QSPR) models estimating blood-to-liver partition coefficients (log P(liver)) for volatile organic compounds. A hybrid approach combining the ML method with descriptor selection based on expert knowledge yielded higher accuracy models. Xu et al. (2017)^22^ have applied knowledge of correlated molecular activities to improve a multitask deep neural network (DNN) model’s predictive performance.

In all these cases of prior knowledge incorporation into predictive models, expertise in the field is of great importance. The enormous volumes of information exist for almost every domain. Researchers have been striving to reduce the amount of manual work required to process this information,^23^ which is a challenging process in itself.^24^ One of the sources of the domain’s prior knowledge is scientific publications. Extraction of knowledge existing in scientific articles is challenging,^25^ but the need for making such knowledge more accessible to researchers and nonprofessional users is growing. For example, Zhang et al. (2019)^26^ have proposed a Solution-oriented Knowledge Repository framework that provides scientific solutions mined from academic articles to the given research problems. Pandi et al. (2020)^27^ have described a text-mining approach to extract pharmacogenomics associations. Guo et al. (2021)^28^ have presented a method for extracting reactions from the chemical literature. Recent commercial software, IRIS,^29^ an AI engine for scientific text understanding, has been developed to facilitate literature review and data extraction from scientific publications. However, to our knowledge, there are no studies examining knowledge mining for predictive modeling.

This work aims to explore knowledge existing in the field of acute aquatic toxicity in a semiautomated way and evaluates a few potential ways of its use in predictive models for initial screening of chemicals. Acute aquatic toxicity testing is an essential element of environmental hazard and risk assessments frameworks and is included in EU chemical legislation.^30^ It plays a critical role in designing molecules with reduced persistency, bioaccumulation in the environment, and toxicity (PBT). There is also a need to develop models that could be integrated into the automated prescreening of chemicals in such applications as, for example, CAMD.^4^ Such applications might require the computation of acute aquatic toxicity values for new chemical structures not empirically tested, thus demanding the existence of quantitative structure–activity relationship (QSAR) models with adequately populated training sets. Therefore, the development of reliable, preferably easy to interpret, *in silico* acute aquatic toxicity prediction models is required to perform an environmental hazard assessment of compounds early in the design phase. The most commonly used *in silico* approaches are QSA(P)R (from now on referred to as QSARs) and read-across (observed similarity of molecular properties between structurally similar compounds) methods. The recent models strive to improve the prediction accuracy by applying machine learning algorithms^31−33^ and consensus modeling based on the prediction made by several models.^31,34^ There has also been increasing interest in developing advanced data-driven methods, e.g., models taking advantage of the knowledge transfer from related tasks^35,36^ to alleviate the molecular data scarcity problem.

On the one hand, the vast amount of research in the field of acute aquatic toxicity makes it an attractive topic for the development of predictive models with the use of knowledge existing in the field. On the other hand, the area is more specific than a more general topic like “environmental safety”. Limiting the subject to a more particular subdomain of the field might aid identification of the relevant research.

The current work aims to address the following questions: How can (semi)automated literature review accelerate knowledge mining? How can this be applied to reveal key factors influencing acute aquatic toxicity as an important safety-related metric for chemicals? In which way could the extracted knowledge be used in predictive modeling? A semiautomated knowledge extraction and the knowledge utilization methods in predictive modeling are proposed in the Methods section to answer these questions. The methods’ implementation is evaluated in the Results and Discussion section for the knowledge extraction part and the assessment of various ways to use this knowledge to hybridize predictive models. The Conclusions section summarizes the main findings and potential extensions of the work.

## Methods

### Knowledge Extraction

**Figure 1:**
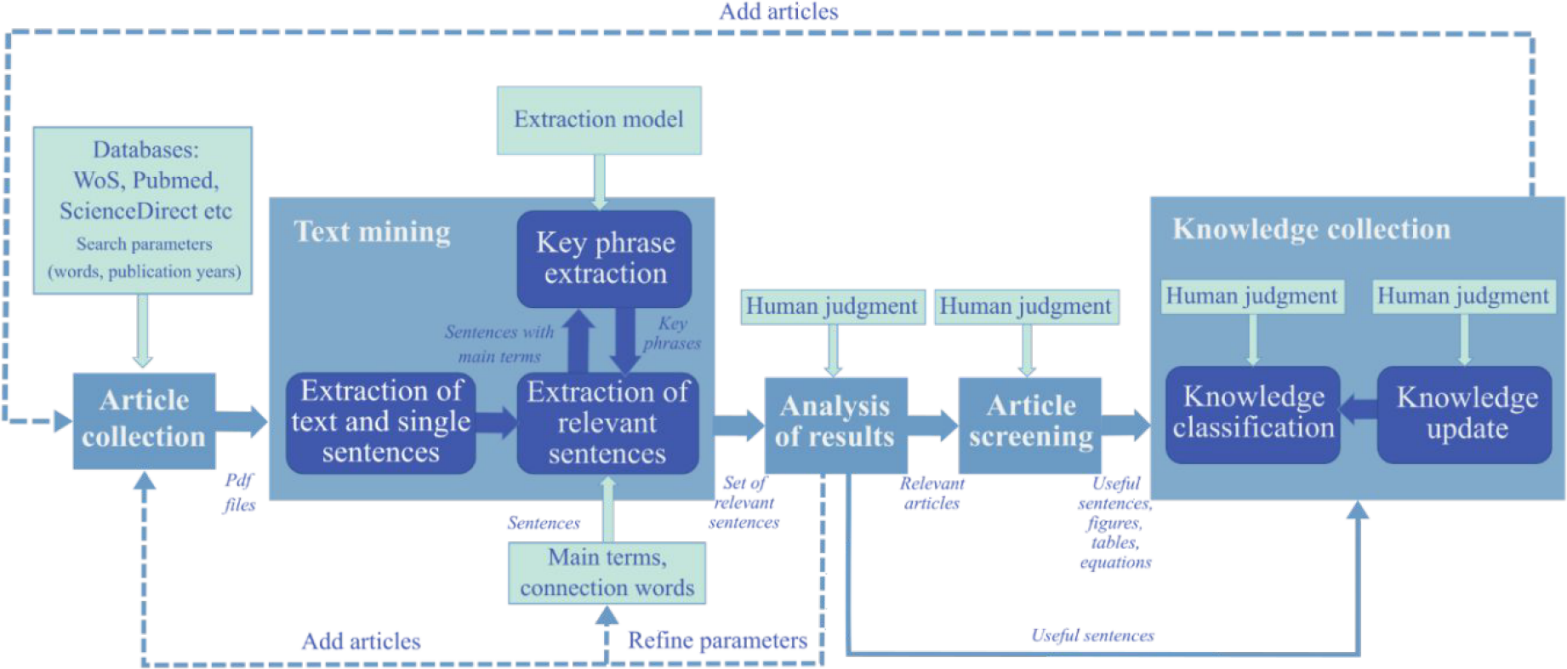
Knowledge extraction method for a specific domain.^37^

The method applied for knowledge extraction from scientific articles presented in Figure 1 combines automated and manual text processing.^37^ The knowledge extraction starts with article collection, followed by text mining, analysis of the obtained results, additional article screening, and knowledge collection. The text mining part was automated, and the results of the automated part provided information that guided the manual processing of the extracted knowledge. The method is best applied in a specific domain; this helps guide the automated knowledge extraction and the manual process requiring human judgment.^37^

The knowledge extraction was performed on scientific articles gathered from the ScienceDirect, PubMed, and Web of Science databases. “Aquatic toxicity” and a period covering 21 years (2000–2020) were used as the search parameters.^37^ Only the articles with titles related to predictive ecotoxicity, QSARs, information on the aquatic toxicity of the separate chemical classes (groups), and modes of action (MoA) were collected. Studies on inorganics, metals and metallorganic compounds, ionic liquids, epoxides, peroxides, and mixtures were excluded. The exclusion of certain groups of chemicals is a standard practice in the domain due to the software’s inability to compute descriptors and/or read SMILEs (simplified molecular-input line-entry systems) of specific chemical classes. For instance, domain knowledge indicates that chemicals with rapidly degrading groups, such as peroxides and epoxides, are very reactive under environmental conditions, and it is recommended to consider the breakdown products instead.^38^ The article collection step resulted in the identification of around 400 publications, which were then used for the text mining.^37^ The bibliometric information on the collected articles is analyzed in Figures S1 and S2 in Supporting Information, S1.

The automated text mining (Figure 1) consisted of three main parts: extraction of article text and single sentences, key phrase extraction, and extraction of the relevant sentences.^37^ First, the text was recognized and precleaned, such that the title, abstract, and references were removed, as well as extra spaces that appeared during the text recognition. The complete sentences of the text were used for the extraction of relevant sentences. The relevant sentences were identified based on the reader-provided input, namely the presence of preselected “main terms”, and “connection words”. The main terms included words “toxicity”, “acute”, “LC50”, and “EC50”. The following words served as the connection words (as complete words or lemmas): “increase”, “decreas”, “relat”, “correlate”, “structure”, “fragment”, “class”, “significant”, “high”, “affect”, “low”, “link”, “reason”, “determin”, “predict”, “influence”, “severe”, and “depend”. The text mining generated a list of relevant sentences for every article.^37^ A python-based package “*knowmine*” was developed to automate the text mining step. A more detailed description of the *knowmine* package can be found in Supporting Information, S2.

The extracted set of the relevant sentences was then evaluated manually to identify useful sentences.^37^ In this study, a sentence was considered useful if it contained information that could be used in predictive modeling (i.e., the sentence referred to aspects influencing acute aquatic toxicity values). The useful sentences were directly collected as knowledge or used to find articles and parts of the text for additional manual screening. The extra screening was performed to extract tables, figures, and equations as well as if the extracted sentence’s information was insufficient or needed clarification for future use (i.e., predictive modeling). The information retrieved in this step was used for structuring the knowledge via the development of a classification and update scheme.^37^ The information extracted from the articles steered the development of the knowledge classification and update schemes.

The update mechanism (Figure S3) was required for cases when the newly extracted information competed or complemented the previously classified information. A detailed description of the method and development of the classification scheme and update mechanism is presented in Supporting Information, S1.

The knowledge classification scheme is generic but in the present study fitted the purpose of identifying and understanding various types of knowledge of the aquatic toxicity field. It should also be remembered that the useful sentences, and thus the article screening and the knowledge collection, were set to contain information describing aspects influencing the chemicals’ acute aquatic toxicity value. This means that the obtained knowledge classification scheme is intended to assist efforts in developing predictive models with the use of the extracted knowledge.

In this sense, it could also be argued that the proposed knowledge extraction procedure is perhaps better described by the term “knowledge distillation”, with respect to the “purity” and loss of retrieved information and the oriented purpose of predictive modeling. However, the term knowledge extraction will be kept in the rest of the paper to describe the proposed approach as it is predominantly used in the relevant scientific literature.

### Predictive Modeling

A complete procedure for designing and implementing a predictive model based on an ML algorithm consists of several steps: task formulation, construction, training on the data, and evaluation of the model and inference regarding its use.^39^ The process does not necessarily follow such a chronological order but is rather iterative. Prior knowledge can be incorporated anywhere in this process.^39^ Some generic examples of the strategies for using prior knowledge in predictive modeling are described in Supporting Information, S3.

#### Data Set^37^

The aquatic toxicity data used in this study were retrieved from the PBT (persistency, bioaccumulation potential, toxicity) database collated by Strempel (2012).^40^ The original database created by Strempel (2012) contains 94,483 chemicals. Chemicals identified as inorganics, epoxides, and peroxides and those with molecular weight > 1,000 were excluded to avoid the errors encountered with prediction tools such as ECOSAR. The ECOSAR databases and the corresponding models were used to obtain the acute aquatic toxicity values for most of the chemicals in the original database. For approximately 2,000 chemicals, the toxicity data were obtained from the Aquire ECOTOX, Canadian Domestic Substance list, and EnviChem databases.^40^ “The most-sensitive species” approach was followed, i.e., the lowest effect concentration with LC50 and EC50 and a duration of either 96 h for fathead minnow or 48 h for daphnia (*D. magna*) was selected. For those chemicals for which no data were available in ECOSAR (7,783 molecules), the baseline toxicity was calculated on the basis of the octanol–water partition coefficient.^37^

Due to the variability of the data sources, data quality, and the absence of an indication of the origin of every value, the data set is associated with some uncertainties and inaccuracies.^37^ For instance, the reported accuracy level (i.e., when the estimated LC50 falls within the same regulatory category, high, moderate, low, no hazard, as the measured LC50) of ECOSAR predictions is only around 60%.^37,41^ Moreover, this data set is characterized by “mixed data” in terms of species, duration class, study type, etc. In this study, instead of targeting homogeneous clusters of data (i.e., where biological activity is measured for all compounds under the same conditions) to formulate a set of QSARs, the modeling approach follows the general concept (i.e., not the methodology) of Sheffield and Judson,^42^ namely it prioritizes having a large amount of data over having a pure data set, based on the assumption that chemical structure is the principal driver of end point variation. This approach avoids limiting the training data by experiment type and leaving the decision for the application and extension of the models to the user; instead it incorporates all commonly available data and “allows the model to adjust its predictions accordingly”.^42^

Another type of applying mixed data for toxicity inference of chemical substances is proposed by the “Guidance on Information Requirements and Chemical Safety Assessment”^43^ published by the European Chemicals Agency, where short-term aquatic toxicity data based on acute test or QSARs can be used for the screening of molecules. The substance is assessed as very toxic if L(E)C50 for algae, daphnia, or fish < 0.01 mg/L. In this case, a definitive conclusion can be drawn that the substance fulfills the T (Toxic) criterion without further testing. If EC50 or LC50 < 0.1 mg/L, the substance is considered as a Potential T (PT) candidate. If EC50 or LC50 ≥ 0.1 mg/L, long-term or chronic aquatic toxicity data is required for a more definitive assessment. Therefore, despite the mixed species approach and uncertainties, the current data set is suitable for use in the study to evaluate in which ways knowledge can be applied to improve the first screening of the substances (Toxic, Potentially Toxic, or Not Toxic) by means of predictive models.^43^

Only saturated aliphatic compounds that contain C, O, H, and N were considered for the study. This reduced data set of 2106 molecules enabled a decrease in the computational time, while testing several ways of prior knowledge use in predictive modeling. The data set is provided as a separate Excel file in the Supporting Information.

#### Predictive Models

In this work, a k-nearest neighbors (kNN) ML approach was chosen as an exemplary algorithm for constructing predictive models with and without prior knowledge. Models that were constructed without using prior knowledge were designated as “standard” models, and the models that did utilize prior knowledge were termed “hybrid” models.^37^ The standard models were developed for purposes of comparison, to evaluate the impact of the knowledge incorporation on the performances of the models. The hybrid models used the extracted knowledge that was relevant to the data set (e.g., concerning the toxicities of aliphatic compounds for fish and crustaceans).^37^

The kNN is an easy-to-implement algorithm without a highly intensive training procedure. This enables quicker testing of different ways of knowledge use in predictive modeling. The kNN estimates a missing property value using the molecules that are structurally most-similar (nearest neighbors) with the known property values.^37^ The nearest neighbors were identified in two ways: (i) a Tanimoto similarity^44^ between molecular fingerprints and (ii) the Manhattan distance^44^ between the molecular descriptor vectors (93 descriptors) representing the chemicals.^37^ The molecular fingerprints and descriptors were computed with the help of the open-source cheminformatics tools RDKit^45^ and PaDELPy.^46^ The optimal number of neighbors was determined by a cross-validation (CV) procedure for validation to training data ratios ranging from 5 to 30%. Out of the numerous CV runs the number of neighbors leading to the highest performance was selected for the final standard models: descriptor (DESC with the number of neighbors equal to 2, 4, 5, 6, and 8) and fingerprint-based (FPN with 2, 5, 7, 12, and 14 neighbors). More details on the development of the standard models can be found in Supporting Information S3.

**Figure 2:**
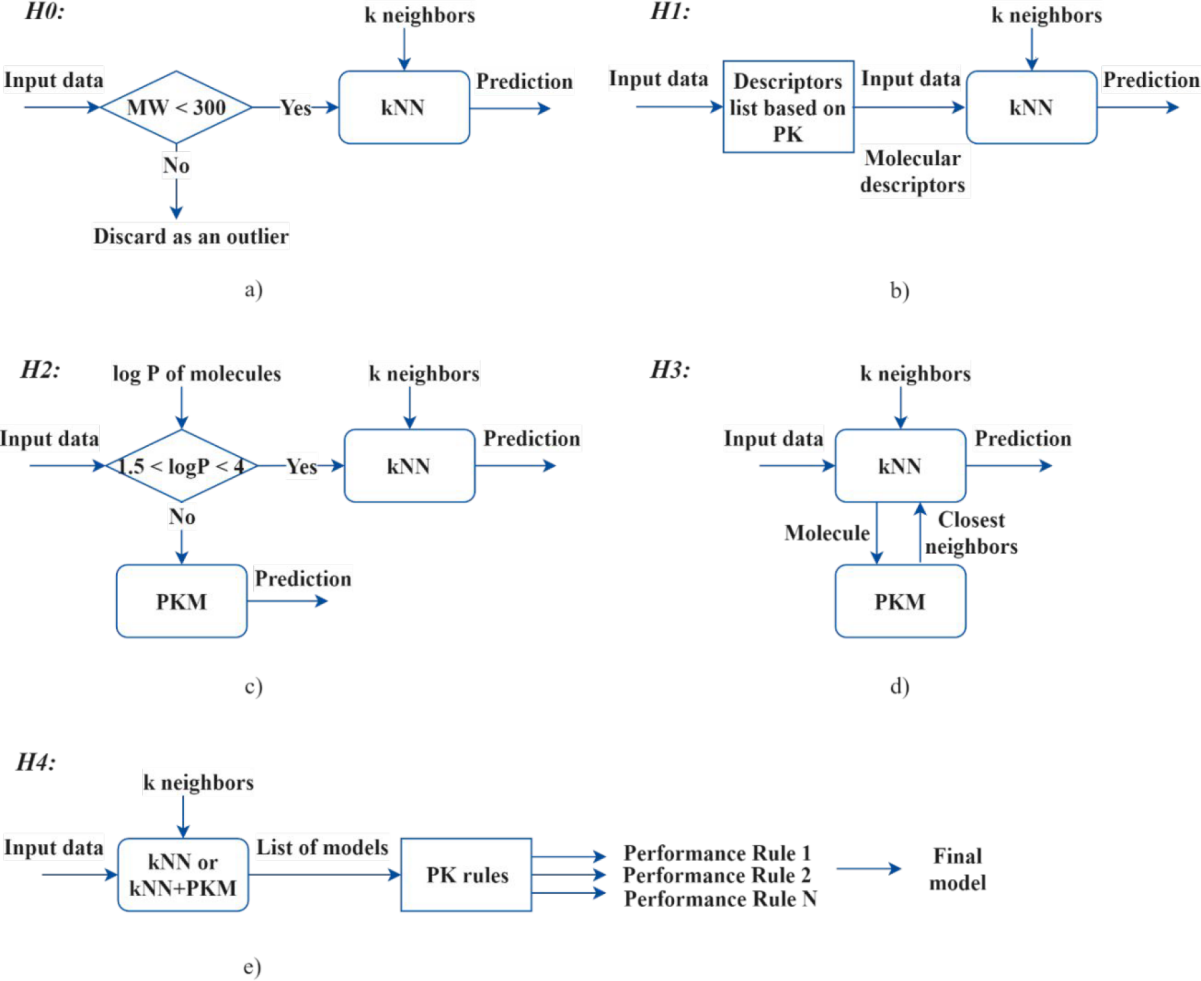
Schematic representation of the use of prior knowledge in the development of hybrid models: PK, prior knowledge; PKM, prior knowledge model (GC+QSAR)^37^

**Figure 3:**
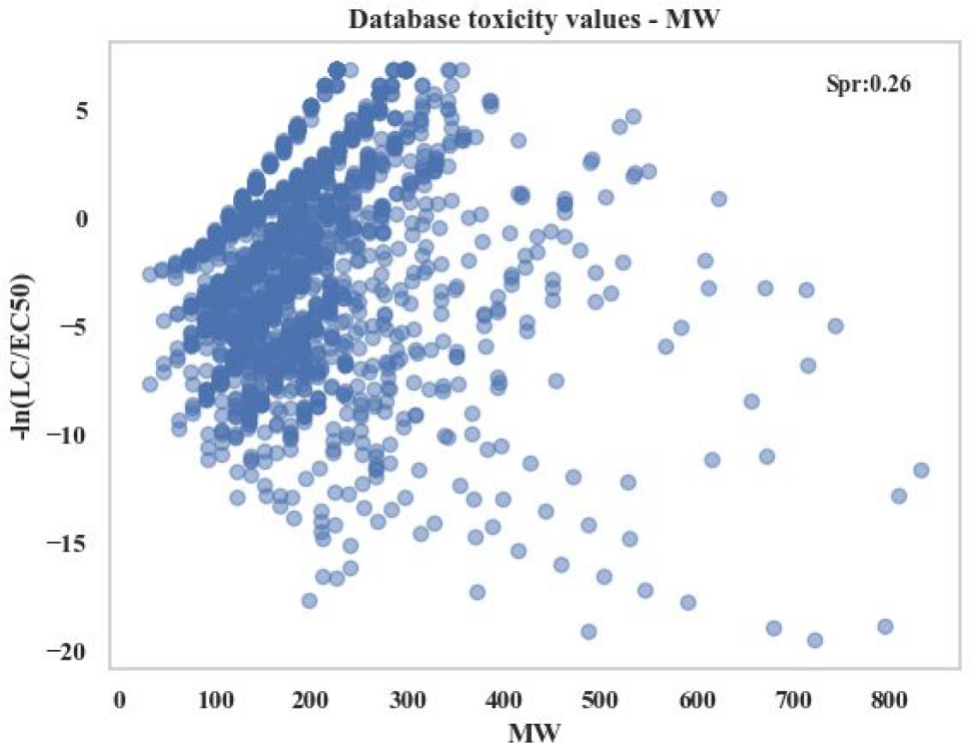
Correlation of data set toxicity (−ln(LC50/EC50)) values with molecular weight.

Prior knowledge was applied before, during, and after the kNN algorithm approach, according to the different schemes^37^ presented in Figure 2. For hybrid model H0 (Figure 2a), a concept of outlier detection based on single descriptor molecular weight (MW) was tested. The hypothesis behind this concept implied the presence of shared outliers between most of the descriptors used for the prediction. For instance, according to prior knowledge, the toxicity of a chemical increases with an increase in its MW. As seen in Figure 3, a correlation between the MW and toxicity values can be observed. However, the correlation is weak for most of the molecules with MW > 300 g/mol. In this way, 187 molecules were removed from the data set as outliers.^37^ A more detailed analysis of the outliers and the tested hypothesis is presented in Supporting Information, S4.

For hybrid model H1 (Figure 2b), a descriptor (predictor) selection was performed, such that from a list of potential molecular descriptors, those descriptors identified by prior knowledge as having a high influence on aquatic toxicity were selected to represent the molecular structures.^37^ It should be noted that these descriptors not only refer to descriptors used in previous QSAR modeling attempts but also incorporate information based on qualitative knowledge extraction. It should also be noted that this hybridization technique could generally involve a more thorough search for subsets of descriptors toward optimality, which however lies outside the scope of this work which aims to exemplify the potential of the knowledge extraction in hybridization methods. In this way, the descriptors “LogP”, “AATSC0p”, “TPSA”, “ETA_dEpsilon_A”, “SHBd”, and “Mi” were selected to represent some of the most frequently mentioned toxicity trends. The trends are relevant for the data set molecules and exhibit a good correlation with the toxicity values (Table 1). In the case of H2 hybridization (Figure 2c), the toxicity of the molecules was predicted using either a kNN algorithm or a prior knowledge-based model (PKM), depending on the value of octanol–water partition coefficients of the molecules log P (i.e., estimated by RDkit^45^). For molecules with log P between 1.5 and 4.0 (811 molecules), the kNN fingerprint-based model was used for estimation of their toxicity. The rest of the toxicity values were predicted by the PKM combining a GC^47^ model for fish fathead minnow with an interspecies equation for daphnia (*D. magna*) fish^48^ toxicity. The daphnia model was chosen as a better fit for the data set since daphnia is generally a more sensitive species than fish (See Data set subchapter). The specific range of log P was selected after the analysis of the standard models’ performance revealing larger prediction errors for molecules with log P in the 1.5–4.0 range. The selected GC approach has been shown to sufficiently cover both areas log P < 1 and log P > 4 based on the list of the used compounds for the regression.^37^

**Table 1:** Set of Rules for Evaluation of the Performances of the Models^37^

main toxicity trends	expressed in descriptors
Toxicity increases with hydrophobicity.^49,50^	Toxicity increases with an increase of MolLogP (RDkit).
Toxicity increases with polarizability.^31,50,51^	Toxicity increases with an increase of molar refractivity MR (RDkit).
	Toxicity decreases with an increase of GATS 1p (PaDELPy).
	Toxicity increases with an increase of AATSC0p (PaDELPy).
Toxicity has a negative correlation with topological polar surface area.^50,52^	Toxicity decreases with an increase in TPSA (RDkit).
Most of the toxic compounds act as hydrogen-bonding acceptors, while the least toxic compounds act mainly as hydrogen-bonding donors.^53,54^	Toxic compounds have lower SHBd (PaDELPy).
	Toxic compounds have lower maxHBint2 (PaDELPy).
There is a positive effect of unsaturation and electronegative atom count.^55^	Toxicity decreases with an increase of ETA_dEpsilon_A (PaDELPy).
Toxicity decreases with increase in ionization potential.^31,51^	Toxicity decreases when Mi (PaDELPy) increases.
	The larger the “GATS1i” (PaDELPy), the less likely the compound will be to react and generate toxicity.
Molecular size and bulk have positive influences on toxicity.^34,50,55,56^	With an increase of MW (RDkit), the toxicity increases.
	Toxicity is higher for higher values of ETA_Alpha (PaDELPy).
There is an inverse effect of branching on toxicity.^50,52,55,57^	Toxicity decreases with an increase of ETA_EtaP_B (PaDELPy).
Toxicities of primary, secondary, and dimethyl tertiary amines increase with increasing chain length.^58^	Toxicity of molecules containing N or amine group increases if the number of carbon atoms increases.
Toxicity increases with increasing alkyl chain length in ethoxylates.^59^	Toxicity of molecules containing the methoxy group increases if the number of carbon atoms increases.
Substitution of H atom with a methyl group (−CH3) on the N atom reduces the toxicity of amine surfactants.^60^	The toxicity of molecules decreases with the number of N–CH3 fragments.

The daphnia PKM model has also been used for the development of the H3 hybrid models.^37^ The models (Figure 2d) applied the PKM model to assist the kNN algorithm in selecting the closest neighbors; molecules with the closest estimations (smallest difference between the predictions) to those obtained by the PKM model were considered the nearest neighbors. Prior knowledge in the form of rules was also applied as a postassessment method for screening the developed models (H4, Figure 2e) to evaluate their performance.^37^ The rules are presented in Table 1, and full names of the descriptors introduced in the rules can be found in Table S16.

## Results and Discussion

This section is divided into two parts. The first part briefly introduces the knowledge extraction and classification results, and the second part presents the usage of prior knowledge for predictive modeling.

### 1 Knowledge Extraction

The main advantage of the partly automated literature review was a significant reduction (Table S1, Supporting Information) of the text for initial reading (>85%).^37^ Most of the sentences extracted by the automated text-mining procedure were useful, in that they could be used for predictive modeling or pointed out specific parts of the initial article for the subsequent manual screening. The method did not seem to require extensive knowledge of the field, as only some prior understanding was needed to define the main terms and connection words that would guide the search for relevant information.^37^ However, it might be useful to run a sensitivity analysis on a limited number of articles to adjust the search parameters if there is no field expertise or it is wished to limit the amount of the extracted information further.

**Figure 4:**
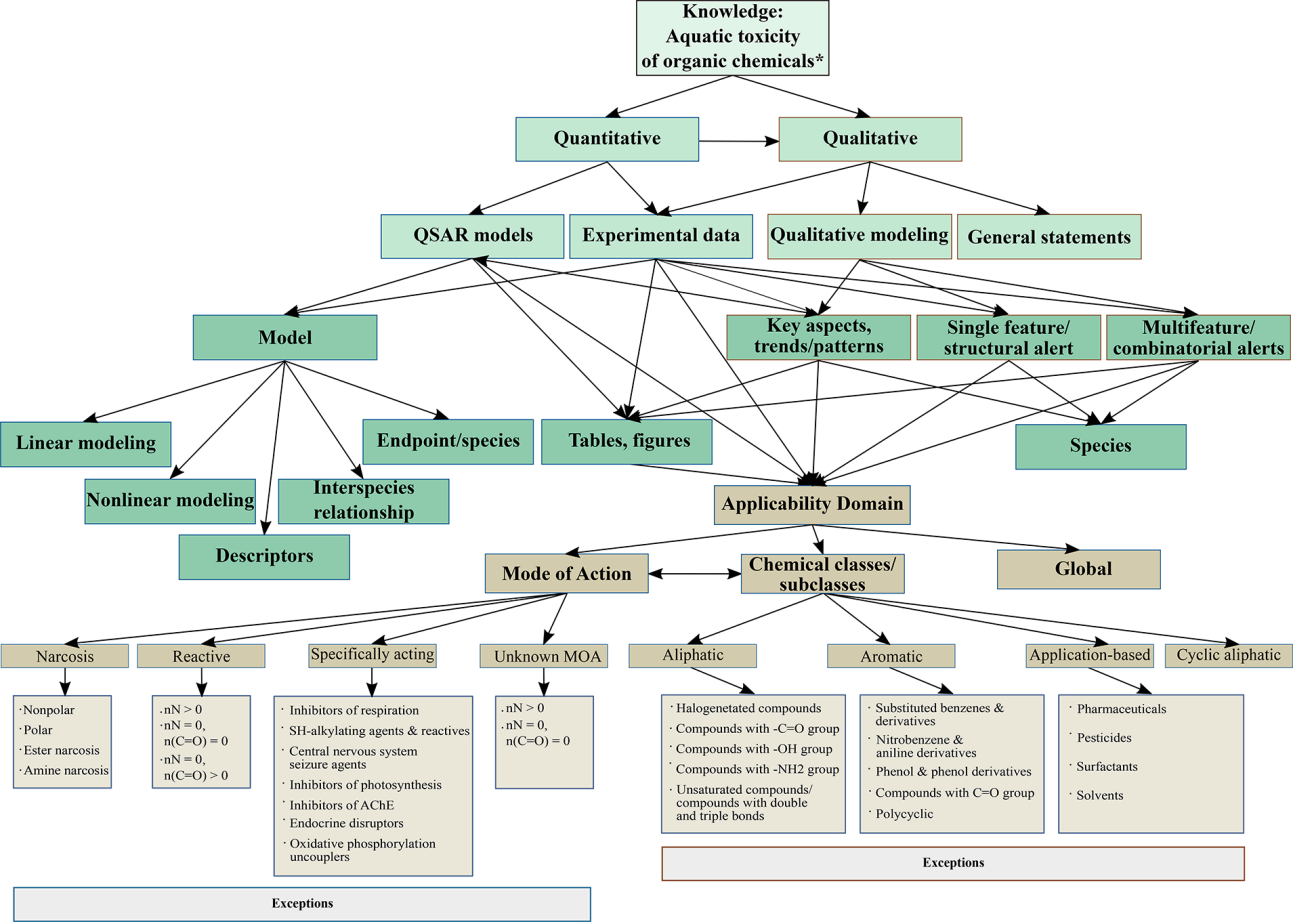
Knowledge classification scheme for aquatic toxicities of chemicals (*excluding inorganics, metals and metalloorganic compounds, ionic liquids, epoxides, peroxides, and mixtures).^37^

The knowledge collected from the scientific articles could be classified under two main categories: quantitative and qualitative information (Figure 4). The quantitative category comprises quantitative structure–activity relationship (QSAR) models and experimental data. The QSAR models differ by the type of modeling (linear, nonlinear) and descriptors used to develop the models. The QSARs define the organism-aquatic toxicity end point relationship for certain species or establish the correlation between end points of two different species (interspecies quantitative activity–activity relationships (QAARs)). The qualitative category contains qualitative modeling and general statements. Qualitative modeling comprises information on key toxicity aspects, trends, patterns, and single feature/structural or multifeature/combinatorial alerts. Compared to other categories, the general statements do not give detailed information about the descriptors or models but make a more generic description of the toxicological properties, models, or data quality. Qualitative and quantitative information could also be presented or analyzed in graphs, figures, and tables. Tables 2 and 3 present examples of prior knowledge collected under quantitative and qualitative categories. The knowledge collected for each category of the classification scheme depicted in Figure 4 is discussed in the following paragraphs in more detail.

**Table 2:** Examples of the Quantitative Knowledge Extracted as Part of the Classification Scheme

applicability domain	model type	end point	descriptors	performance^a^	ref
global	linear modeling (MLR+GA)	–logLC50 *Pimephales* promelas	AlogP, E_LUMO_, S2K, nRNH2	*n* = 408, *R*^2^ = 0.80	Pavan et al. (2006)^61^
				*Q*^2^_LOO_ = 0.80, *Q*^2^_Boostr_ = 0.80, *Q*^2^_ext_ = 0.72	
MoA, specifically acting chemicals	linear modeling	log(1/LC50) *Poecilia* reticulata	*E*_a_(max), ∑C_a_, Nv1	*n* = 31, *R*^2^ = 0.77	Raevsky et al. (2009)^62^

a Selected performance indicators from the respective original article.

**Table 3:** Examples of the Qualitative Knowledge Extracted as Part of the Classification Scheme

applicability domain	species, end point	extracted knowledge	ref
substituted benzenes	*Tetrahymena pyriformis*	positive correlation with end point:	Gupta et al. (2015)^63^
	pIGC50	- MW,	
		- nAtomP,	
		- TopoPSA	
		negative correlation	
		- SHdsCH,	
		lipoaffinity index	
pharmaceuticals	*D. magna*, fish	higher toxicity to *D. magna*:	Kar et al. (2018)^64^
	LC50	- keto group	
		- aasC fragment	
		higher toxicity to fish:	
		- keto group,	
		- X=C=X fragment,	
		- R–C(=X)–X fragment,	
		R–C≡X fragment	

#### QSAR Models

The QSAR models collected during the knowledge acquisition were developed for prediction of the toxicity values or classification of chemicals according to different toxicity levels and MoA classes.^37^ Most of the QSARs applied linear modeling (e.g., multilinear regression, principal component analysis, linear partial least-squares, ordinary least-square method) due to its simplicity and interpretability. The nonlinear models often exhibited higher levels of accuracy than the linear models built using the same set of chemicals. A general outcome from the QSAR studies was that ensemble or consensus models that combined several methods outperformed the models based on a single method.^65−68^ Improved performance was also observed when similar chemicals were grouped based on MoA^69^ or other similarity criteria^69,70^ before developing the prediction model.^37^

A wide variety of descriptors were used in the collected QSAR models.^37^ The descriptors with the highest impact on the acute toxicity value were associated with hydrophobic features (i.e., log Kow, logP, logD, Crippen logP, B08[C–C]), electrophilicity (i.e., ELUMO, Amax), polarizability (i.e., α, GATS 1p), acceptors and donors of hydrogen bonds (i.e., Ca, NHdon Hacc, polar groups descriptors), molecular size and branching (i.e., Vm, ElipVol, RDCHI), and polar surface area (i.e., TPSA). According to Gramatica et al. (2018),^71^ the nX (number of halogen atoms) and nBondsM (number of multiple bonds) descriptors, which are related to halogen substitution and unsaturation, increase the PBT behaviors of chemicals. The descriptors linked to the decrease in the PBT index were MAXDP2 (maximal electrotopological positive variation) and nHDonLipinski (number of donor atoms for H bonds). These two descriptors encode the ability of a chemical to form electrostatic and dipole–dipole interactions, as well as hydrogen bonds in the surrounding environment. Additionally, descriptors connected to the ionization potential were reported as an important parameter influencing the toxicity of the compounds.^31,72−74^ Ionization was shown to affect the biouptake and mechanisms of interaction with the macromolecule at the target sites.^72,73^ Hossain and Roy (2018)^75^ and Önlü and Saçan (2018)^76^ have developed QSAR models for Contaminants of Emerging Concern (CECs), including for instance pharmaceuticals, personal care products, pesticides, and surfactants. They have determined that the toxicities of CECs are mostly related to hydrophobicity,^75,76^ aromaticity,^75^ polarizability, and molecular size and shape.^76^

Various species were used to obtain toxicity information.^37^ The most commonly used species in the QSAR and other types of studies were the algal *Tetrahymena pyriformis (T. pyriformis)* (IGC50), crustacean *Daphnia magna (D. magna)* (EC50, LC50), and fish *Pimephales promelas* (fathead minnow) (LC50). The interspecies QSARs can be regarded as a separate class of the QSAR models. These models are typically based on a small volume of data and have a linear functional form with a few predictor variables.^37^ The fish-based model was recognized to be superior for predicting lacking toxicity data (i.e., for *T. pyriformis* and *D. magna*).^48^ The collected QSA(A)Rs, including details on the corresponding models and symbols, can be found in Tables S3–S7.

#### Sensitivity of Species

The smaller species like bacteria, algae, or crustaceans were found to be more sensitive than fish. However, the sensitivity of species varied depending on the type of chemicals they were exposed to. For instance, *Vibrio fisheri* was sensitive to parabens,^77^ nitrates,^78^ benzoic acids,^79^ and alkoxy-substituted benzenes.^78^*Chlorella vulgaris* was very sensitive to haloalkanes,^80^ while *Pseudokirchneriella subcapitata (P. subcapitata)* showed increased sensitivity to nitriles^81^ and, in general, to organic pollutants.^82^ High sensitivity to aromatic amines and highly lipophilic compounds^34^ was observed for daphnids.^83,84^ The skin and lipid content of multicellular organisms like daphnia and fish could prevent the biouptake for ionizable compounds; thus, the toxicity effect would be decreased.^51^

The algal *T. pyriformis* showed less sensitivity than other species, which might imply less experimental uncertainty of toxicity data available for this species.^85^ Although fish is frequently used for tests, Rawlings et al. (2019)^86^ argue that it is advisible to invest in algal and daphnids testing resulting in more conservative predictions than any fish.

#### Identified Toxicity Alerts, Trends, and Patterns

The collected knowledge^37^ suggests a consensus among researchers that acute toxicity is defined by the mode of toxicological action and the chemical characteristics.^87^ The higher toxicity values have often been associated with increased lipohilicity.^50,88,89^ The most toxic compounds were hydrophobic and acted as hydrogen-bonding or electron acceptors (e.g., hydrophobic nitroaromatic compounds with halogen and amino substituents^52,53,81,90,91^). Khan et al. (2019)^92^ have advised that if a hydrophobic group is necessary during the design of a drug compound, a higher polarity substitution should be preferred. Voutchkova et al. (2011)^93^ have suggested keeping logPo/w < 2 and Δ*E* (LUMO–HOMO) > 9 eV to increase the likelihood of designing a compound with low aquatic toxicity.^37^

Specific functional groups, such as cyano,^87^ isothiocyanate,^94^ and halogens^88,95−97^ enhance the toxicities of molecules.^37^ However, the extent of the increase appears to be dependent upon the molecular structure and position of the group in the molecule. Among the other reported toxicity alerts were amino groups, the presence of additional (one or more) aromatic rings with highly electronegative substituents close to each other (5–7 Å apart),^98^ nitro group, nitrile, disulfide, phosphoric acid derivatives, pyrazolyl group, and formamide groups,^99^ ring aromaticity, sulfur, aromatic esters, and vinyl moiety,^52^ double and triple bonds, and acrylate groups,^100^ to name the most frequently encountered.^37^ Table S2 of the Supporting Information presents examples of molecular features reported to increase or decrease toxicity. The extended version of the table containing the information collected under the qualitative category is available on request from the corresponding author.

#### Applicability Domain

Affinity for a specific chemical class or MoA was often seen as a critical determinant for predicting and understanding chemical toxicity,^101−104^ with MoA being more challenging to determine.^37,105^ The most covered applicability domain in MoA seemed to be nonpolar and polar narcosis, followed by specifically acting chemicals.^37^ The chemical classes that were most highly represented in the collected knowledge base were nitrobenzene and phenol derivatives, pesticides, pharmaceuticals, and halogenated aliphatics. Other chemical classes such as aliphatic alcohols, amines, amides, and acids were represented to a lesser extent, probably because their toxicity effects are instead studied in the context of a particular MoA. Compounds with double and triple bonds, such as vinyl/allyl group-containing chemicals, nitriles, propargyl alcohols, carbonyl-containing α,β-unsaturated chemicals, carbamates, and quinones, have often been examined separately, likely because of their reactive nature.^57,97,106−108^ Despite the clear benefits of assigning compounds to certain chemical classes or MoAs, many researchers strive to develop “global” models^37^ that were not limited by chemical class or MoA.^109^

The academic field of aquatic toxicity is diverse and extensive, both from the quantitative and qualitative perspectives.^37^ On the one hand, this diversity might foster the identification of relevant information, which could be used for predictive modeling. For example, the extracted knowledge can guide predictor (variable) selection and prioritization. Moreover, the experimental data collected from various studies can be used for training or external validation of the developed models. Interspecies correlations could help to close the data gaps present for data sets of certain species. Information on the species’ sensitivity and outliers discovered during the construction of the QSAR models could be used to explain the results or observed deviations in the predictions. The QSAR equations and alerts could be directly integrated into the training phase of the data science models as additional constraints or applied for model refinement. The discovered aquatic toxicity trends and patterns could be helpful for the analysis of the results obtained by the developed models, thus contributing to evaluation and selection of the optimal models. On the other hand, the wide variety of descriptors used in the studies, different quality of the toxicity data, applicability domain limitations, to name some important factors, make it quite challenging to apply directly the knowledge without analyzing the available information and constraints associated with its use. Thus, mapping and evaluating domain knowledge before its application could be helpful to facilitate navigation of the data.^37^ The results of some strategies for using prior knowledge in predictive modeling are presented in the next section.

### 2 Predictive Modeling

The summary of the performances of the final standard and hybrid models can be seen in Table 4. It is evident that the hybridization improves the coefficient of determination (*R*^2^) and Spearman’s correlation coefficient (Spr_m) of the standard descriptor-based models.^37^ The fingerprint-based models (both standard and hybrid) show lower values of *R*^2^ and Spr_m. Additionally, classification metrics were applied for the performance analysis. For the classification metrics, the data set toxicity values and all the predictions made by the models were divided into three categories: T, PT, and NT depending on the values (<0.01 mg/L, <0.1 mg/L, and ≥0.1 mg/L respectively). Then the classified predictions were assessed via balanced accuracy, recall, and precision. All the hybrid models except H3 improve the results of the classification, compared to the standard models’ performance. It should, however, be considered that the hybridization of the descriptor-based models was performed using the same data set as for the standard models. The QSAR-based model used for hybridizing the fingerprint-based models was built on a different set of data, which to some extent explains the lower performance. The method, however, might be used as a way to analyze and deal with the uncertainty of the data set. Moreover, there may be a trade-off between the level of prediction and classification accuracies for a specific data set and qualitative assessment of a model based on the identified prior knowledge trends, as will be discussed next.

**Table 4:** Summary of the Performances of the Models^a^

model	*R*^2^	Spr_m	accuracy	precision	recall
DESC_2	0.83	0.94	0.87	0.96	0.96
DESC_4	0.85	0.95	0.87	0.96	0.96
DESC_5	0.85	0.95	0.88	0.97	0.97
DESC _6	0.86	0.95	0.87	0.96	0.96
DESC _8	0.86	0.95	0.85	0.96	0.96
DESC_H0_3	**0.95**	**0.98**	**0.91**	**0.98**	**0.98**
DESC_H0_7	**0.95**	**0.98**	**0.90**	**0.97**	**0.97**
DESC_H1_2	**0.92**	**0.98**	**0.91**	**0.98**	**0.98**
FPN_2	0.70	0.84	0.56	0.85	0.84
FPN_5	0.74	0.86	0.53	0.84	0.85
FPN_7	0.74	0.86	0.50	0.83	0.84
FPN_12	0.73	0.86	0.51	0.84	0.85
FPN_14	0.73	0.86	0.50	0.84	0.85
FPN_H2_2	0.43	0.80	**0.74**	**0.90**	**0.87**
FPN_H2_7	0.45	0.80	**0.74**	**0.90**	**0.88**
FPN_H2_12	0.46	0.80	**0.74**	**0.90**	**0.88**
FPN_H3_12	0.52	0.76	0.54	0.87	0.88
FPN_H3_14	0.53	0.76	0.53	0.86	0.88

a DESC and FPN specify the descriptor-based and fingerprint-based models, respectively. H0, H1, H2, and H3 are the applied types of hybridization models. The designations _2 to _14 indicate the numbers of closest neighbors used for the prediction. The classification metrics uses three labels (T, PT, and NT) and considers the label imbalance.

**Figure 5:**
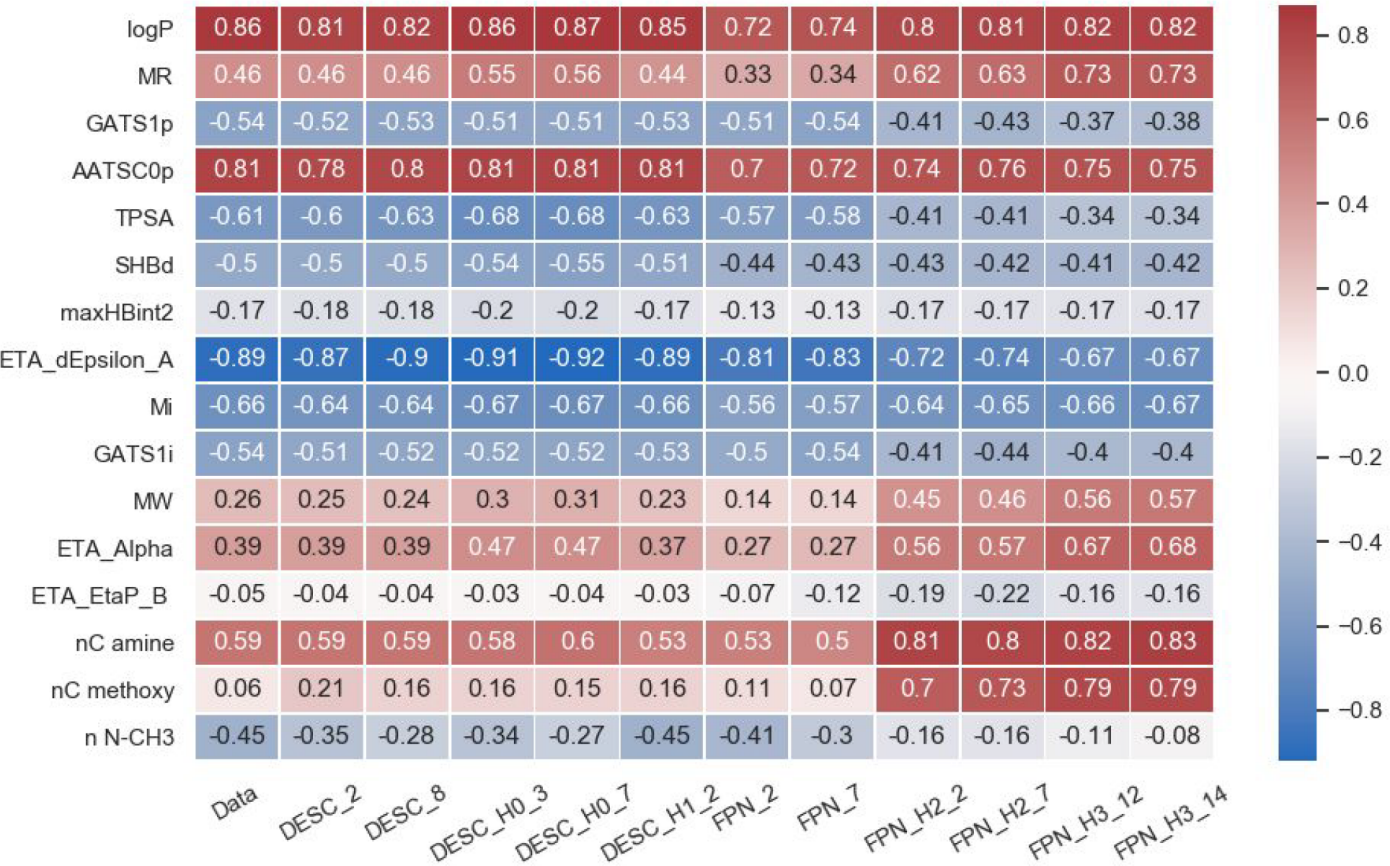
Spearman’s correlation coefficient between the descriptors (Table 1) and toxicity predictions made by the models. Red: positive correlation with toxicity, blue: negative. Only two descriptor and fingerprint-based models (best and worst) are shown due to the similar performance of the rest of the standard models to the presented ones.^37^

The heatmap in Figure 5 depicts how closely the predicted toxicity values follow the rules presented in Table 1. It is clear that some of the rules are better followed by the data set toxicity values (Data) than others. The rules related to maxHBint2 (hydrogen bonding) MW, ETA_EtaP_B (branching), and the methoxy group (nC methoxy) are supported to a lesser extent.^37^ Poor performance of the data set regarding the methoxy group-containing molecules might imply a deviant behavior of these molecules compared to the remainder of the data set. The low diversity of the data set in terms of branching might be the reason for a very low correlation of toxicity values with the ETA_EtaP_B descriptor. The predictions made by the standard descriptor-based models (DESC_2 and DESC_8) (except nC methoxy) show similar trends to the Data. The fingerprint-based models (FPN_2 and FPN_7) show lower levels of compliance with the rules than the descriptor-based models and the Data.^37^

The values for the hybrid models often show a better correlation with the rules.^37^ Interestingly, the highest variation between the results of the standard and the hybrid models is observed for the fingerprint-based models, which are the models with the lower prediction accuracy compared to the standard or descriptor-based hybrid models. Since different data and model parameters were used to develop the prior knowledge model, the hybrid models reveal trends not observed in the data set. A much higher influence of molecular size (MW, ETA_Alpha, partly MR) on the toxicity is noticed for the hybrid models. The toxicity values predicted by the models H2 and H3 exhibit better correlations with the increasing chain length in amines and methoxy group-containing molecules. However, models H2 and H3 seem to be inferior in considering the electronegativity and topology of molecules (lower correlation for GATS 1p, TPSA, GATS1i, ETA_dEpsilon_A) compared to the standard models. The models H2 and H3 do not capture the influence of N–CH3 fragments on the toxicity as well, probably due to the absence of this fragment’s toxicity contribution in the PKM GC method. The performance of H1 models is similar to or slightly lower than the rest of the descriptor-based models and Data. This suggests that a limited number of descriptors can fully represent the molecules in the data set.^37^

The analysis (Supporting Information, S4) of the outliers identified on the basis of MW (model H0) showed that these molecules also differ in logP, MR, TPSA, and ETA_Alpha values compared to the rest of the data set. The set of the outliers seems to consist of highly hydrophobic molecules exhibiting high toxicity and the low toxicity compounds with the reduced ability to permeate the cells of the living organisms due to their larger TPSA and size.

**Figure 6:**
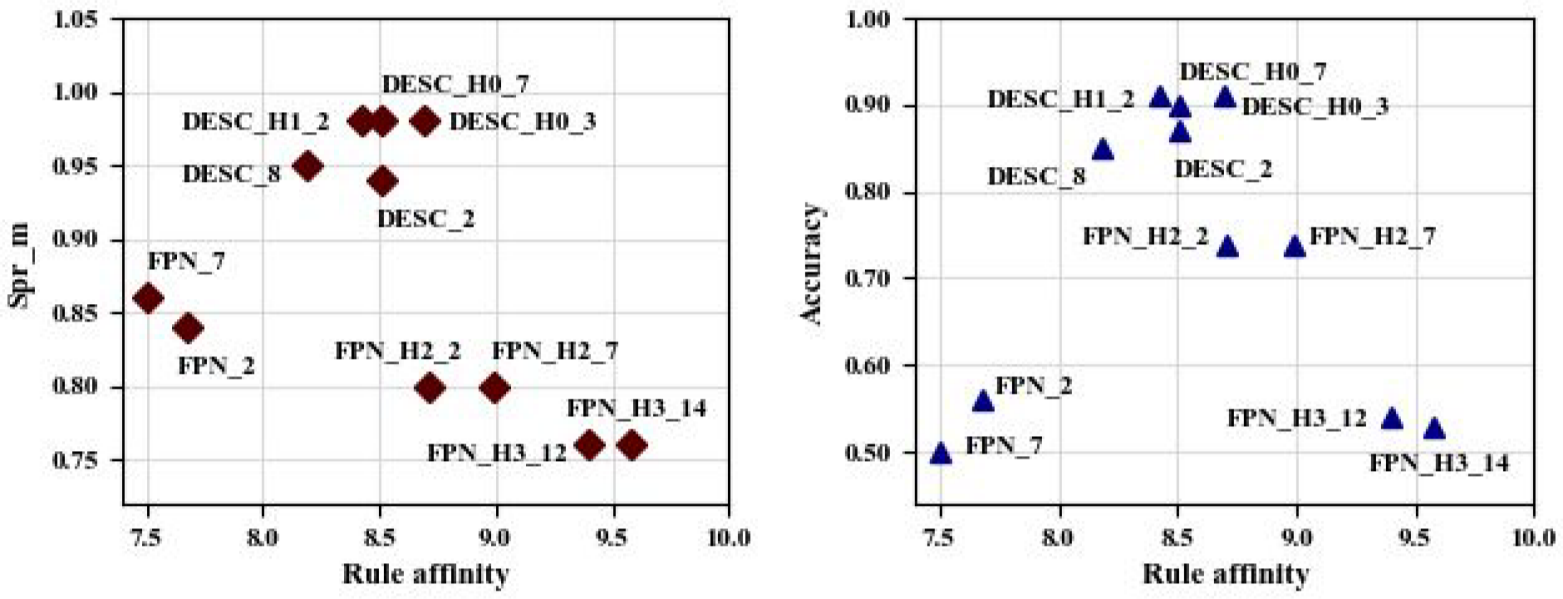
Spearman correlation coefficient (Spr_m) (left) and accuracy scores (right) vs Rule affinity for the standard and hybrid models.

Figure 6 presents the performances of the models in terms of the Spearman correlation coefficients, classification accuracies, and overall compliance of the models with the prior knowledge-based rules (Table 1 and Figure 5). The compliance with the rules, or rule affinity, was computed by first min-max normalization of the correlation coefficients within the same descriptor category followed by the summing of the normalized values over all the descriptors. In this multidimensional assessment it can be seen that the hybrid models DESC_H0_3 (part of the molecules are removed as outliers) and FPN_H2_7 (prediction is made by either standard or knowledge-based model depending on the value of log P) present some interesting optimality characteristics. Model DESC_H0_3 shows the best affinity with the rules compared to the rest of descriptor-based models and has the highest Spearman coefficient and accuracy. Model FPN_H2_7 demonstrates a good compromise between the correlation coefficient and accuracy and compliance with the rules.

The assessment presented in Figures 5 and 6 can generate ideas on developing hybrid models further toward an optimization-based approach (i.e., populating the Pareto-front of Figure 6 by more models, etc.). For instance, a combination of the knowledge-based approaches (i.e., H0 and H2, using MW and log P values) might be a better alternative to predict the toxicity values of the molecules of the data set.

The models are subjected to uncertainty due to the use of the mixed data set. However, the models are useful to compare the aquatic toxicity of a vast number of molecular structures (e.g., generated by CAMD) on the basis of a unified modeling framework and thus systematically reduce the number of chemical alternatives for later screening stages where rigorous experimentation and testing can be applied. Furthermore, the models illustrate the application of prior knowledge for the development and evaluation of hybrid models, which as a concept and methodology is valid independently from the nature of the mixed data set.

## Conclusions

This work demonstrated a systematic, semiautomated extraction and classification of knowledge in the field of acute aquatic toxicity and tested some ways of its integration into predictive models, which can be particularly useful in early design phases, where a vast number of chemicals should be screened, as in the case of CAMD approaches.

The semiautomated procedure of knowledge extraction significantly reduced the manual work required to process a large number of scientific articles while extracting generic and case-specific models, statements, and alerts. The automated text extraction might lead to the loss of valuable information; thus, the combination of the automated procedure with the manual text mining safeguards for the critical loss of relevant knowledge. The semiautomated knowledge extraction can be of assistance in interdisciplinary research when quick knowledge acquisition is required for different purposes (e.g., impact assessment). However, it should be noted that the semiautomated approach may still be introducing biases (although in a less subjective way than purely human-centric approaches). The bias can be introduced either through the standardization approaches when screening the vast amount of textual information or by the human-machine interaction in the form of keywords as input for the text mining approach and analysis of the extracted information and knowledge classification.

The knowledge collection and classification procedure can be useful in hybrid modeling studies concerning the model and predictor selection, prioritization, and constraints, addressing data gaps, and validating and interpreting model performance. The study demonstrated how the incorporation of prior knowledge improved the performance of the predictive models either in prediction accuracy or compliance with previously observed trends from the extracted prior knowledge. Furthermore, it was shown that the knowledge could be used in a variety of ways not only during the development of the models but also before and after for data analysis, model selection, evaluation, and adjustment.

The presented knowledge extraction method and approaches for knowledge incorporation into predictive models are generic and can be used in many other knowledge domains. The knowledge extraction method can easily incorporate more resources (in terms of amount and type), while the classified knowledge allows for more hybrid alternatives, also depending on the machine learning approach used (i.e., neural networks and deep learning approaches, classification trees, random forest regression, etc.). Thus, the presented hybridization methods should only be considered as examples of integrating the results of semiautomated knowledge extraction in the concept of hybrid modeling, and certainly diverse hybridization approaches can extend the presented concepts (e.g., using semiquantitative toxicity information not directly suitable for model calibration but possibly providing information to steer the calibration in the proper direction and test the results). Additionally, the knowledge extraction introduces a secondary model assessment beyond the prediction accuracy, namely the degree of compliance with different trends previously observed in the investigated knowledge domain. This makes it possible to apply multiobjective optimization in the development of predictive models, either with or without hybridization. Thus, more insights into model selection can be provided leading to more robust model development.

## Data and Software Availability

The knowmine package is available for installation via pip; the source files can be retrieved from https://github.com/GulnaraSh/Knowledge-mining-python. The titles of the input articles used for the knowledge mining are available by request from the corresponding author.

## Supporting Information Available

Details on knowledge extraction, collected QSA(A)Rs, and description of text mining knowmine package and models’ development (PDF)
Data set of 2106 molecules containing smiles and acute aquatic toxicity values (XLS)
