# Supplementary material for: Prior Knowledge for Predictive Modeling: The Case of Acute Aquatic Toxicity
Source: J Chem Inf Model. 2022 Aug 23;62(17):4018–31. doi: 10.1021/acs.jcim.1c01079 (PMC9472271; doi:10.1021/acs.jcim.1c01079)
Supplement: Supplementary file 1 — ci1c01079_si_001.pdf [file ci1c01079_si_001.pdf]

Supporting Information:

Prior knowledge for predictive modeling: the case of acute aquatic toxicity

Gulnara Shavalieva[1], *Stavros Papadokonstantakis*[1,2]*, *Gregory Peters*[3]

[1]Chalmers University of Technology, Department of Space, Earth and Environment, Division of Energy Technology, SE-412 96 Gothenburg, Sweden

[2]TU Wien, Institute of Chemical, Environmental and Bioscience Engineering, Getreidemarkt 9, 1060 Vienna, Austria

[3]Chalmers University of Technology, Department of Technology Management and Economics, SE-411 33 Gothenburg, Sweden

*Corresponding author: stavros.papadokonstantakis@tuwien.ac.at

ABBREVIATIONS

A. flos-aquae – freshwater cyanobacteria Aphanizomenon flos-aquae,

C.carpio – European carp Cyprinus carpio,

CPANN – counter-propagation artificial neural network,

C. pyrenoidosa – freshwater algae Chlorella pyrenoidosa,

C. vulgaris – microalga Chlorella vulgaris,

D. magna - planktonic crustacean Daphnia magna,

DTB – decision tree boost,

DTF – decision tree forest,

D. tertiolecta – algae Dunaliella tertiolecta,

GA – genetic algorithm,

GC – group contribution,

kNN – k-nearest neighbours,

L – linear models,

LDA – linear discriminant analysis,

L. gibba – plant Lemna gibba,

L. macrochirus (bluegill) – fish Lepomis macrochirus,

LR – linear regression,

NL – nonlinear models,

(A)NN – (artificial) neural networks,

N. pelliculosa – diatom Navicula pelliculosa,

MLR – multilinear regression,

O.mykiss (rainbow trout) – fish Oncorhynchus mykiss,

PCA – principal component analysis,

PLS – partial least squares,

P. promelas (fathead minnow) – fish Pimephales promelas,

P. reticulata (guppy) – fish Poecilia reticulata,

P. subcapitata – microalga Raphidocelis subcapitata,

RBFN – Radial basis function network,

R – random forests,

R. japonica – plant Rohdea japonica or frog Rana japonica,

S. costatum – marine diatom Skeletonema costatum,

S. obliguus – algae Scenedesmus obliquus,

S.quadricauda – freshwater microalgae Scenedesmus quadricauda,

S. subspicatus – algae Scenedesmus subspicatus,

SVM – support vector machines,

T. pyriformis – algae Tetrahymena pyriformis,

V.fischeri – bacteria Vibrio fischeri

S1. Knowledge extraction and collection

Article collection

The knowledge extraction was performed on scientific articles collected from ScienceDirect, Pubmed, and Web of Science[1]. “Aquatic toxicity” and a period of 21 years, from 2000 to 2020, were used as search parameters. The search resulted in the identification of thousands of publications, and these articles could be collected and analyzed by the proposed method. However, to reduce the amount of manual work, the domain was defined in an even stricter way. Only the

articles with titles related to predictive ecotoxicity, QSARs, information on the aquatic toxicity of the separate chemical classes (groups), and modes of action (MoA) were collected. Studies on inorganic, metals and metallorganic compounds, ionic liquids, epoxides, peroxides, and mixtures were excluded. The exclusion of certain groups of chemicals is a standard practice in the domain due to the inability of the software to compute descriptors and/or read SMILEs (simplified molecular-input line-entry systems) of specific chemical classes. Chemicals with rapidly degrading groups like peroxides and epoxides are very reactive under environmental conditions, and it is recommended to consider the breakdown products instead[2]. The article collection step resulted in the identification of around 400 publications[1]. Analysis of bibliometric information of the collected articles is presented in Figures S1 and S2. It can be seen that the distribution over the publication years is relatively even, but the highest number of papers is from the last three years (Figure S1a). About thirty authors, having the number of articles equal to or exceeding five (Figure S1b), seem to be publishing more in this domain. Around 4% of the investigated papers seem to dominate the citations (Figure S1c).

Visualization in Figure S2 performed with the help of VOSviewer[3] presents a term map for words with minimum co-occurrence equal to ten. The map is based on text data of titles and abstracts of the articles. The size of the circle and the word label show the importance of the term. The larger the circle and the label, the more frequent the term is. More correlated terms have a shorter distance between the circles. The color of a term circle indicates the average year of the publications containing the term. The lines between the terms represent co-occurrence links. The higher is the number of publications in which two words occur together, the stronger the link is[3]. According to the figure, the earlier studies seem to be dealing with modes and mechanisms of toxic action, analysis of the relationships between structure, molecular descriptors, and toxicity. The

later publications address prediction models and their performance. Overall, model-related topics also seem to dominate the research area of the collection of articles. The list of articles used for text mining is available from the corresponding author on request.

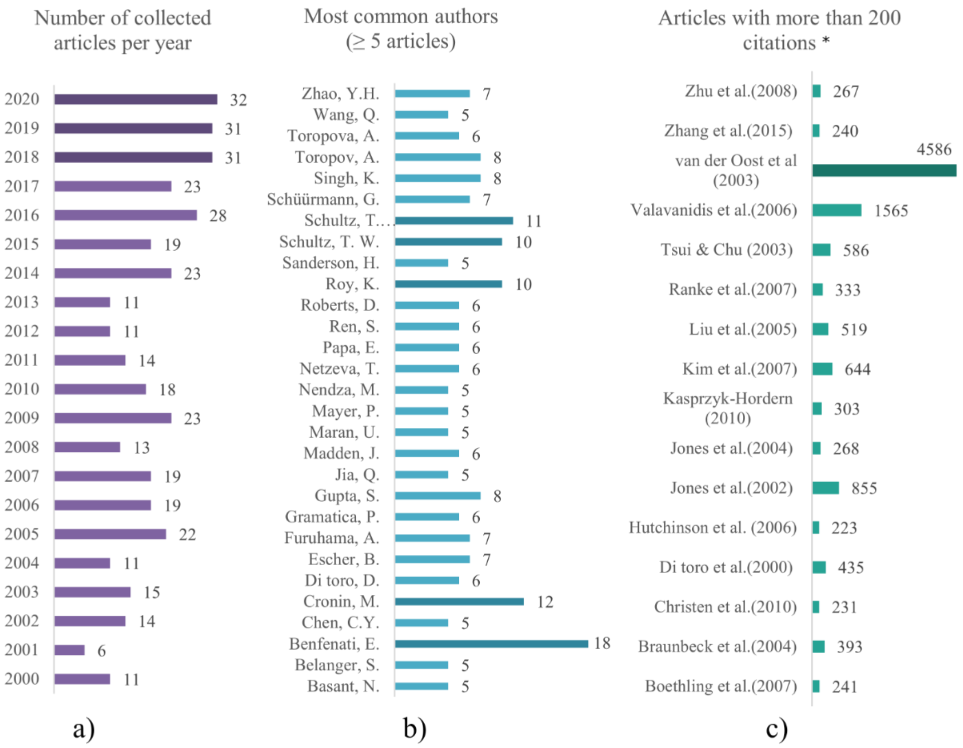

Figure S1. Analysis of articles' bibliometric information. *-number of citations at the time of the article collection, retrieved from Google scholar.

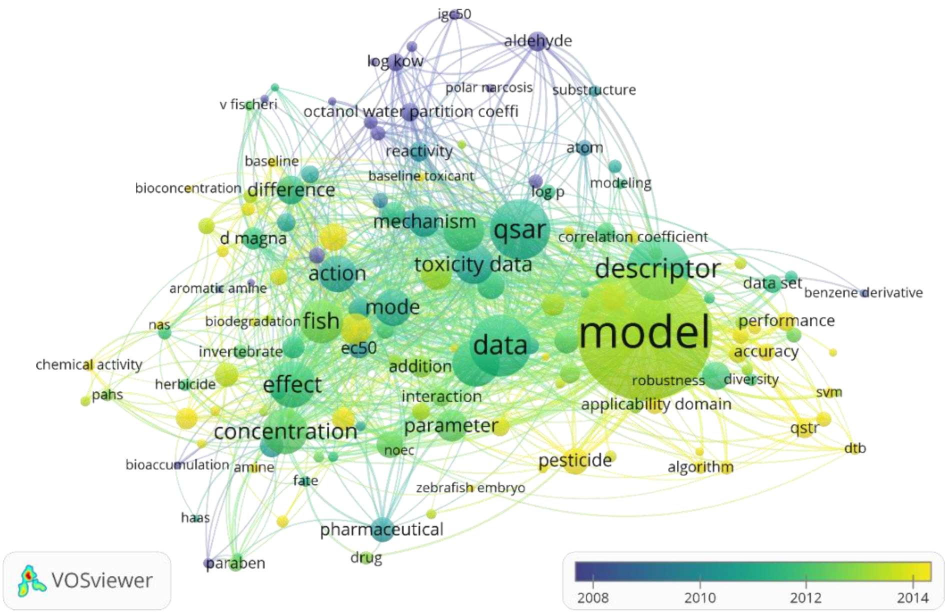

Figure S2. Term co-occurrence map (minimum co-occurrence = 10)

Text mining

A python-based package "*knowmine*" was developed containing several modules that automate text mining. The automated text mining consisted of three main parts: extraction of article texts and single sentences, key phrase extraction, and extraction of the relevant sentences[1]. First, the text of the articles was recognized and pre-cleaned: the title, abstract, and references were removed, as well as extra spaces appearing during the text recognition and e-mails. After the text precleaning, single sentences were identified based on sentence terminators (dot, exclamation and question marks, quotation marks, brackets). The identified single sentences were then checked for completeness using the library for natural language processing spaCy[4]. Sentences containing less than two nouns and one verb were discarded as incomplete. The complete sentences proceeded to the extraction of relevant sentences step. Relevant sentences identification was performed during this step based on the reader-provided input, namely the presence of preselected "main terms", and "connection words". First, main terms were used to reduce the number of sentences to those that

include any main terms (e.g., toxicity, acute, LC50, EC50). Then key phrases were extracted from this reduced set of sentences using the open-source python-based "pke"[5] package with the implemented graph-based keyphrase extraction model SingleRank[6]. Then the indicated sentences (i.e.: those with the main terms used to extract the key phrases) whose key phrases contain the main words, and the connection words were extracted as relevant sentences. The main terms included words "toxicity", "acute", "LC50", "EC50". The following words served as the connection words (as complete words or lemmas): "increase", "decreas", "relat", "correlate", "structure", "fragment", "class", "significant", "high", "affect", "low", "link", "reason", "determin", "predict", "influence", "severe", "depend". The text mining resulted in a list of relevant sentences for every article[1]. A more detailed description of the text mining and *knowmine* package can be found in chapter S2.

Analysis of results and article screening

The extracted set of the relevant sentences were then manually evaluated to identify useful sentences[1]. The potential use of the extracted knowledge defines the usefulness of the sentence. In this study, a sentence was considered useful if it contained information that could be used in hybrid predictive modeling (i.e., the sentence refers to aspects influencing acute aquatic toxicity values). The useful sentences were collected either as directly extracted knowledge or used to identify articles and parts of the text for additional manual screening. The extra screening was needed if the extracted sentence's information was insufficient or required clarification for future use (i.e., hybrid modeling). The article screening was also performed to extract tables, figures, and equations since it was not yet possible to retrieve all of them automatically in a readable format – however, only articles identified by the analysis of the results as relevant (those that contained relevant sentences) underwent the screening. The analysis of the extracted sentences and/or article

screening can be iterated based on altered input parameters (i.e., main terms and connection words, extraction model) and addition of articles. The information retrieved in this step in the form of useful sentences, models, figures, and tables was used for structuring knowledge via developing a classification scheme in the next step[1].

Knowledge collection

The information extracted from the articles published in 2000-2014 (225 articles) was used to develop the initial classification scheme. The information extracted from the rest of the articles (165 articles) was used to update the classification scheme and demonstrate a procedure to combine the classification scheme with information acquired in the future. The mechanism for developing and updating the classifications scheme is presented in Figure S3.

The update mechanism outlines a set of actions taken when the newly extracted information competes or complements the previously classified information. The newly extracted information is considered to compete with the previously classified if it provides the same type of information, for instance, a QSAR for the same species and class of chemicals using the same molecular descriptors as predictor variables. When this kind of information improves the results of the previous studies (e.g., the QSAR model showed better performance and developed based on a larger dataset), then the newly acquired knowledge replaces the previously collected one; otherwise, it can be discarded or stored depending on the purpose of the classification. The new information could also contradict the previously extracted one, for instance, when the QSAR model descriptors are reported to have a positive correlation with the toxicity endpoint instead of the negative as reported by the information analyzed earlier. In that case, the decision can be to either replace the previously collected knowledge, not to replace it but store the new information

or completely discard it. The previously collected knowledge is replaced if the new information is supported by sufficient evidence, e.g., extensive experimental work. Suppose there is insufficient evidence supporting the new knowledge, but a new and sufficiently diverse argument is provided. In that case, the new information is stored until the claim is supported by more evidence (experiments, more studies). For instance, in the example mentioned before, where new knowledge claims that the presence of a particular structural feature can increase the toxicity of a molecule, which is against what had been previously reported. A new suggestion could be that this behavior is better explained by considering a combination of structural features instead of depending only on a single feature. In case of poor argumentation, the new information could be stored or discarded based on user preferences regarding the size of the knowledge structure and the future use of this structure. It is also possible that this step requires an in-depth metadata analysis by the user to reveal the causes of potential discrepancies between the compiled facts to make a more informed decision. If the new information complements the previously extracted information by providing additional depth (e.g., QSAR for the same class of chemicals but based on a different set of molecular descriptors or species), the new information is added to the previously collected information. If the new information does not satisfy the criteria to be considered competing or complementary knowledge, then the information is collected under a new classification category.

The knowledge classification scheme is generic, but in the present study, it fits the purpose of identifying and understanding various types of knowledge existing in the aquatic toxicity field. It should also be remembered that the useful sentences, and thus the article screening and the knowledge collection, were set to contain information describing aspects influencing acute aquatic toxicity value. This means that the obtained knowledge classification scheme may assist future

efforts in developing predictive models for the aquatic toxicity of chemicals, where previous knowledge is combined with machine learning towards superior hybrid predictive models.

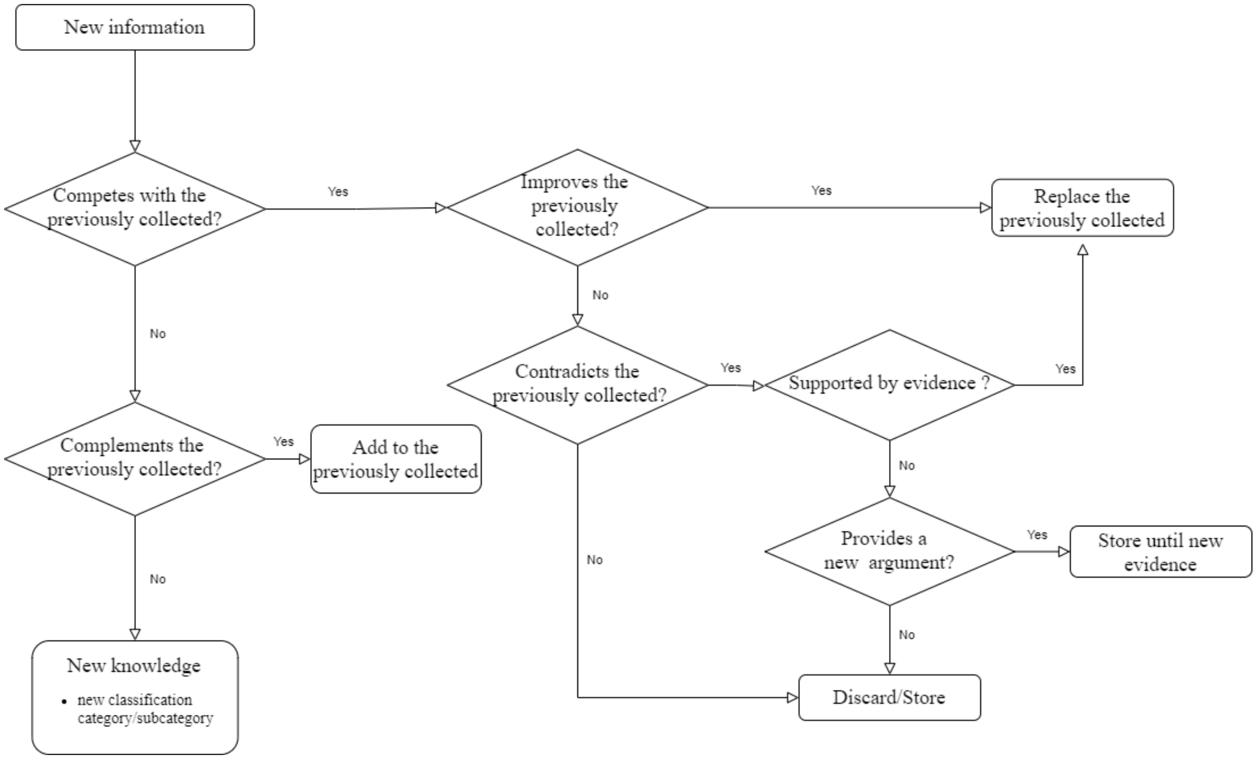

Figure S3. Update mechanism for knowledge collection and classification

Knowledge extraction

Table S1 shows the results of the knowledge extraction procedure in quantitative terms. The main advantage of the partly automated literature review was a significant reduction of text for initial reading (>85%)[1]. The analysis of the results also led to the identification of three extra articles (Ellison et al.(2008)[7], Tan et al. (2010)[8], Alves et al. (2016)[9], Gini et al.(2019)[10]), which were used for an additional iteration.

The knowledge was classified into two main categories: quantitative and qualitative information. Table S2 presents examples of the qualitative information. The extracted quantitative information

in the form of quantitative structure-activity (property) and structure-activity-activity relationships (QSA(A)Rs) can be found in Tables S3-S7.

Table S1. Results of knowledge extraction in quantitative terms.

**Text mining (based on 2000-2020 articles)**	
Number of sentences (excluding abstract, acknowledgments, and references) in articles, per article on average	~165
Number of extracted sentences as relevant, per article on average	~20
**Update mechanism (based on 2015-2020 articles)**	
**Quantitative knowledge**	
Number of competing QSA(A)R models*	-
Number of complementing QSA(A)R models*	~240
Number of new categories/subcategories	~15
**Qualitative knowledge (Qualitative modeling)**	
Number of competing pieces of information	
- Added	~20
- Discarded	~10
- Contradicting/ stored	~3
Number of complementing pieces of information	~380
Number of new categories/subcategories	~20
**Collected knowledge (based on 2000-2020 articles)**	
Number of QSA(A)R models*:	

Linear	~430
Nonlinear	~180
Interspecies correlation models	~50
Number of different descriptors	more than 600
Number of different species	~50
Number of alerts	~240
Number of key aspects/pattern/trends	~650
Number of general statements	~20
Number of different classes/subclasses	~40
Number of Tables	~280
Number of Figures	~40

*Number of QSA(A)R models for the same subclass

Table S2. Examples of molecular features reported to increase or decrease toxicity.

	Toxicity increase	Toxicity decrease
Property	Lipophilicity, hydrophobicity, electrophilicity increase[11–14]	LogPo/w < 2 and ΔE (LUMO-HOMO) > 9 eV[15]
Structure	Cyano, isothiocyanate, halogens (enhanced by activation (e. g., adjacent to an ester or other unsaturation)), amino group, nitro group, nitrile, disulfide, phosphoric acid derivatives, pyrazolyl group, formamide groups, ring aromaticity, sulfur, aromatic esters vinyl moiety, double and triple bonds, acrylate, carbamate groups[16]<br>Molecular bulk (size) increase[17,18]<br>Increase in unsaturation[19]<br>N-N bond[20]	Higher polarity substitution of a hydrophobic group[21]<br>Presence of nitrogen in sp2 state[22]<br>Simultaneous presence of Sulfur together with double bond[22]
Position	Additional (1 or more) aromatic rings with highly electronegative substituents close to each other (5–7 Å apart)[23]	Branching at the α- or β-carbon[26–28]

	Aromatic ring:<br>- Two hydroxyl and/or amino groups in ortho and para orientation[24]<br>- NO_2 groups in ortho position to the -OH group[18]<br>Bulky substituents around positions 3 and 4 and near the heteroatoms of the side chain[25]	Fluoro and ether functionality in benzenes[29]

Table S3. QSAR models for different chemical classes/subclasses.

Applicability Domain	Models	Endpoint, species	Descriptors	Performance	Reference
			Aliphatic compounds		
Nonspecific aliphatic toxicity	L	log (1/IGC50), T. pyriformis	logKow, -Elumo	n=353, R^2_{adj} = 0.859	Schultz et al.(2002)[30]
	L	-log(LC50), fathead minnow	logP, -(E_{HOMO}-E_{LUMO}), Vm/XYZ	n =106, R^2 = 0.832, R^2_{cv} = 0.812	Colombo et al.(2008)[31]
			Halogenated aliphatic		
Halogenated + alkanones, alkanals, and alkenals	L	pT_{15}, V.fischeri	logP, -E_{LUMO}	n = 63, r^2 = 0.846,	Cronin et al.(2000)[26]
Halogenated esters, alcohols, nitriles	L	log(1/IGC50), T.pyriformis	logKow, -E_{LUMO}	n=18, R^2=0.740,	DeWeese& Schultz (2001)[27]
α- haloactivated compounds [RC(X)C(=O)R or RC(X)C(#N)R]	L	log(1/IGC50), T.pyriformis	-E_{LUMO}, A_{max}, ElipVol	n=30, r^2 adj.=0.831, r^2 pred.=0.792	Schultz et al.(2002)[30]
Chlorinated alkanes	L	Fish P. reticulata log LC50, correlation with in vivo data	logKow	n=18, R^2=0.883	Zvinavashe et al.(2008)[32]
			Compounds with carbonyl group		
Compounds containing a carboxylic acid moiety [RC(=O)O]	L	log(1/IGC50), T. pyriformis	logKow, -E_{LUMO}	n=35, r^2adj.=0.873, r^2 pred.=0.838.	Schultz et al.(2002)[30]
Carboxylic acids and their derivatives	L	-log(LC50), fathead minnow	$^3\chi$,-^{0}CIC, FNSA3PM3, minEstate(C), logD6.5	n =28, R^2=0.935, R^2cv=0.906	Colombo et al.(2008)[31]
Carboxylic acids	L(11)	pIGC50, T.pyriformis	electrophilicity index (ω, ω^2, ω^3), logP, $logP^2$	n=28 R^2=0.750-0.937	Jana et al. (2020)[33]
Simple aldehydes	L	log(1/IGC50) T. pyriformis	log Kow, -E_{LUMO} (not significant)	n=17, r^2=0.898	Schultz et al. (2002)[30]
Aliphatic and aromatic aldehydes	L	-logLC50, fathead minnow	Best model: ClogP, -FNSA-3 fractional PNSA (PNSA-3/TMSA) [Zefirov's PC], -HA dependent HDCA-1 [Gaussian NBO PC], ΔQ_{CO} NBO	n=50, R^2=0.868, R^2_{CV}=0.840	Smiesko&Benfenati (2004)[34]

Aliphatic and aromatic aldehydes	L	pLC50, mix of fishes	MlogP2, B08[C-C], B02[C-C], -B05[C-C], Fr5(elm)/C_C_C_H_O/1_2s, 2_3a, 3_5s, 4_5s/, F04[O-O]	n_{tr}=39, n_{test}= 13, R^2=0.840, R^2_{pred}=0.860 CCC=0.920	Khan et al.(2019)[35]
Aliphatic and aromatic esters	L	log1/LC50, Pimephales Promelas<br><br>log1/EC50 Daphnia<br><br><br><br>log1/EC50 in algae	DRAGON descriptors MATS4v, -REIG<br><br><br><br>-TIC0, -nCp, n=CH2<br><br><br><br>DISPp, H8u<br><br><br><br>ESter Aquatic Toxicity INdex (ESATIN): -SHP2, n=CH2, DISPp	n_{tr}=24, n_{test}=6, R^2_{adj}=0.823, Q^2_{LOO} =0.785 Q^2_{EXT}=0.715<br>n_{tr}=24, n_{test}=5, R^2_{adj}=0.860, Q^2_{LOO} =0.831 Q^2_{EXT}=0.790<br>n_{obj}=11, R^2_{adj}=0.949, Q^2_{LOO} =0.923<br>n_{tr}=31, n_{test}=30, R^2_{adj}=0.898, Q^2_{LOO} =0.873 Q^2_{EXT}=0.866	Papa et al.(2005)[36]
Esters	L	pLC50, P. promelas	VP-2, maxHdsCH	n=30, R^2=0.88, CCC_{est}=0.95	Gramatica et al.(2014)[37]
Esters	L	pEC50, D.magna	VP-2, - nsCH3, minHdCH2	n=29, R^2=0.86, CCC_{est}=0.87	Gramatica et al.(2014)[37]
Monoesters	L(11)	pIGC50, T.pyriformis,	electrophilicity index (ω, ω^2, ω^3), logP, $logP^2$	n=31 R^2=0.756-0.933	Jana et al(2020) [33]
Diesters	L(11)	pIGC50, T.pyriformis,	electrophilicity index (ω, ω^2, ω^3), logP, $logP^2$	n=20 R^2=0.739-0.912	Jana et al(2020) [33]
Ketones	L(11)	pIGC50, T.pyriformis,	electrophilicity index (ω, ω^2, ω^3), logP, $logP^2$	n=15 R^2=0.779-0.975	Jana et al(2020) [33]
Compounds with hydroxyl group					
Long chain alcohols C6-22	L	Log EC50, D.magna	-logKow	R^2 = 0.981	Fisk et al. (2009)[38]
Linear alcohols C2-12	L	log(LC50), zebrafish Danio rerio and fathead minnow (juvenile and embryo)	-logKow	R^2 = 0.954-0.990	Belanger et al.(2018)[39]
Saturated alcohols	L(11)	pIGC50,	electrophilicity index (ω, ω^2, ω^3), logP, $logP^2$	n=32	Jana et al(2020) [33]

		T.pyriformis,		R^2=0.715-0.983	
Amino alcohols	L	log(1/IGC50)<br>T. pyriformis	$-E_{HOMO}$, $^3\chi^{vp}$,<br>-logH (Henry's law constant)	n=16, r^2adj.= 0.841,<br>r^2pred.=0.788	Schultz et al.(2002)[30]
Amino alcohols	L(11)	pIGC50,<br>T.pyriformis,	electrophilicity index (ω, ω^2, ω^3), logP, $logP^2$	n=18<br>R^2=0.387-0.879	Jana et al. (2020)[33]
Compounds with amino group					
Amines [RCN]	L	log(1/IGC50)<br>T. pyriformis	logKow	n=30, r^2adj.=0.873,<br>r^2pred.=0.848.	Schultz et al.(2002)[30]
Amines	L	pLC50,<br>mix of fishes	-BLTD48, ALogP2, F03[C-S],<br>S_A(chg)/A_C_C_D/1_2s, 1_4s, 3_4s/6,<br>Fr5(d_a)/A_A_A_I_I/1_4s, 2_5s, 3_5d,<br>4_5s/ , -S_A(rep)<br>/B_C_C_C/1_3s, 1_4s/4,	n_{tr}=69,<br>n_{test}= 23,<br>R^2=0.800, R^2_{pred}=0.820<br>CCC=0.890	Khan et al.(2019)[35]
Amides	L	pLC50,<br>mix of fishes	X1 sol, B07[C-N], F02[C-S], -F03[C-S],<br>H-047	n_{tr}= 24, n_{test}=8,<br>R^2=0.940, R^2_{pred}=0.950<br>CCC=0.970	Khan et al.(2019)[35]
Unsaturated compounds/Compounds with double and triple bonds					
α-unsaturated (triple bond) alcohols (i.e., proelectrophiles)	L	log(1/IGC50), T. pyriformis	log Kow, Elumo, $^3\chi^C$	n=20, r^2adj.=0.842,<br>r^2pred.=0.803	Schultz et al.(2002)[30]
Vinylene-containing-α,β unsaturated esters	L	log(1/IGC50), T. pyriformis	logKow, AC_2	n=15, r^2adj.=0.823,<br>r^2pred.=0.885	Schultz et al. (2005)[40]
Ethynylene-containing α,β unsaturated esters	L	log(1/IGC50)<br>T. pyriformis	logKow	n=10, r^2adj=0.932,<br>r^2pred=0.919	Schultz et al. (2005)[40]
α,β-Unsaturated Carbonyls	L	log(1/IGC50),<br>T. pyriformis	log (1/RC50) (reactivity)	n=41, r^2=0.846	Yarbrough &Schultz(2007)[28]
α,β-Unsaturated Carbonyls	L	log EC50<br>T. pyriformis	-logKow, -logkGSH	n=57, R^2=0.85	Böhme et al (2016)[41]
Nitriles	L	log(1/EC50),<br>P. subcapitata	logKow,<br>$-E_{LUMO}$	n=9, R^2=0:92; Q^2 =0.51	Huang et al.(2007)[42]
Compounds with a triple bond, and specifically, propargyl alcohols and nitriles	L	-log(LC50),<br>fathead minnow	logD7.4, -maxEen(C–H), Q_{max},<br>-minEexc(C–H), -^{0}BIC	n=22, R^2=0.972,<br>R^2cv=0.954	Colombo et al.(2008)[31]
Propargylic alcohols	L	log(1/EC50),<br>P. subcapitata	Primary: logKow<br>Tertiary: logKow<br>Secondary: logKow, -ELUMO	n=15, r^2=0.76,Q^2 =0.69<br>n=8, r^2=0.97, Q^2=0.94<br>n=7, r^2=0.85	Chen et al.(2012)[43]

Unsaturated alcohols	L(11)	pIGC50, T.pyriformis,	electrophilicity index (ω, ω^2, ω^3), logP, logP^2	n=25<br>R^2=0.296-0.890	Jana et al.(2020)[33]
Vinyl/Allyl/Propargyl moiety containing chemicals	L	pLC50, mix of fishes	X0 sol, S_A(lip)/B_B_C_C/2_4s,3_4s/4, O-057, Fr5(en)/B_B_C_C_D/1_4s, 2_4s, 3_4s, 3_5s/, nOHt, B03[N-Cl], B04[N-O]	n_{tr}=56, n_{test}=15, R^2=0.740, R^2_{pred}=0.790<br>CCC=0.860	Khan et al.(2019)[35]
α,β-unsaturated aldehydes	L	log(1/IGC50)<br>T. pyriformis	logKow, -E_{LUMO}, -(QC4 + QC3)	n=14, r^2adj=0.966, r^2pred=0.934	Schultz et al. (2005)[40]
α,β-unsaturated ketones	L	log(1/IGC50)<br>T. pyriformis	-(QC4 + QC3), -E_{LUMO}	n=16, r^2adj=0.917, r^2(pred)=0.860	Schultz et al. (2005)[40]
Alkenes and poly-alkenes with isolated double bonds, acrylates, saturated and α,β-unsaturated aldehydes and ketones.	L	-log(LC50), fathead minnow	ZX, maxEc(C-H), minEtot(C–C), -maxNC, maxEstate(C)	n=50, R^2=0.896, R^2cv=0.872	Colombo et al. (2008)[31]
Aromatic compounds					
	L	log(1/IGC50),<br>T. pyriformis	logKow, Amax	n=385, r^2adj=0.859	Schultz et al. (2003)[44]
	L	1/EC50, C.vulgaris	logKow, -ELUMO, Amax, ${}^0\chi^V$	n=65, r^2=0.86, q^2=0.84,	Netzeva et al. (2004)[45]
	NL	Algae Scenedesmus quadricauda, Daphnia spinulata, Bryconamericus iheringii, EC/LC50	1/exp(logKow)	r=0.999-0.897	Marzio&Saenz(2006)[46]
	L	log(1/IGC50),<br>T. pyriformis	topological index/heavy atoms, logKow, net charge of C atoms, surface charge/surface area,mass of fragments, Bonds number/energy	n=200, r^2=0.756, r^2cv=0.739	Laszlo&Beteringhe (2006)[47]
	L	log(1/IGC50),<br>T. pyriformis	-contain Y-H (Y=O, N) bonds "able to form H bonds":<br>N or arom bonds/N bonds, moment of inertia, logKow, mass of fragments, reaction index<br><br>-without Y-H bonds "unable to form H bonds":<br>net charge of C atoms,Cl atoms, Beteringhe descriptor, Lumo-Humo gap/ mol volume, Bonds number/energy	n=87, r^2=0.796, r^2cv=0.761<br><br>n=113, r^2=0.895, r^2cv=0.882	Laszlo&Beteringhe (2006)[47]

	L,<br><br><br>NL	-log(IGC50),<br>T. pyriformis	TPSA(NO), log P, ME2	n=288,<br>R^2_{adj}=0.851,<br>R^2_{CV}=0.846<br>R^2_{tr}=0.909, R^2_{test}=0.927	Lei et al.(2008)[48]
	NL<br>(SVM)	log(1/IGC50),<br>T. pyriformis	HOMO, LUMO energy, ΔE, the total molecular energy (ETot), the minimum (QNmax) and the maximum (QPmax) atomic partial charge, dipole moment (μ),polarizability (α); Heat of formation (HF), molecular surface area (MSA), molecular volume (MVol), logP, hydration energy (HE), molecular refractivity (MR), MW, Kier and Hall simple and valence-corrected molecular connectivity indices (χ); Kappa shape indices (κ); shape flexibility (Φ); Wiener, Randic and Balaban topological indices; E-state indice (S); the number of H-bond donors (NHdon) and acceptors (NHacc); atom counts (oxygen, nitrogen, fluorine, chlorine, bromine, iodine, halogen atoms, heteroatoms); group counts (hydroxyl, amino, aldehyde, nitro, cyano, acid anhydride, methyl) (Table 4)	n=81, R^2=0.84	Su et al. (2017)[49]
	L<br>(MLR,<br>PCA)	-logEC50,<br>C. vulgaris	E, E_{HOMO}, -E_{LUMO}, -qH+, q-, $C_{v\theta}$, S_θ, V_i,	n=20, R^2=0.95 n_{test}=5,<br>$R^2_{MLR\ test}$=0.5689<br>$R^2_{PCR\ test}$=0.678	Yang&Wang (2017)[50]
Substituted benzene & derivatives					
	L, NL<br>(NN)	-logLC50,<br>fathead minnow	Best LR: topostructural indices ($^0\chi$, P_9, IC), topochemical index ($^5\chi^v$), geometrical index ($^{3D}W_H$), quantum chemical descriptors (ΔH_f, μ).<br>NN: 95 parameters	LR: Explained variance R^2 = 86.1%<br>NN: R^2 = 0.868	Basak et al.(2000)[51]
	L	log(1/IGC50),<br>T. pyriformis	Optimization of Correlation Weights of Local Graph Invariants	n =157, r^2=0.883	Toropov&<br>Shultz (2003)[52]
	L	logLC50,<br>fathead minnow	Optimization of correlation weights of Morgan extended connectivity	n=44, r^2=0.89,<br>r^2tr=0.90	Toropov&<br>Toropova(2002)[53]
	L	pC, fish guppy	ETA parameters: ∑α, $[\eta'_F]_{Cl}$, $-[\eta'_F]_{N\text{-}UNS}$,<br>$-[\eta'_F]_{NO2\text{-}O\text{-}CL}$, $-[\eta'_F]_{OEt}$, $[\eta'_F]_{OH}$, $[\eta'_F]_{C\text{-}SP3\text{-}NO2}$	n=92, Q^2=0.865,<br>R_a^2=0.876, R^2=0.885,<br>R=0.941	Roy&Gosh(2004)[29]

	L	-log(1/LC50),<br>fathead minnow	Spectral moments μ^{D}_{5}, $\mu_1\mu_1{}^{H}$, -$\mu^{Dist}{}_{1}$<br><br>DRAGON descriptors: nX, SCBO,-Me	n=50, R^2=0.888<br><br>n=50, R^2 =0.8095<br>Q^2=0.7692	Gonzalez et al.(2005)[54]
	L	log(1/IGC50),<br>T. pyriformis	Atom-based non-stochastic linear indices<br>Atom-based stochastic linear indices	n=307, R^2 = 0.791,<br>R^2_{pred} =0.762<br>n=308, R^2 = 0.799,<br>R^2_{pred}=0.797	Castillo-Garit et al. (2008)[55]
	L, NL (RBFNNs)	pC, P.reticulata (fish)	nCl, -FPSA3, TMSA, HASA2/TMSA, SIGMA-PIMax BO	n=92(74+18)<br>L:<br>R^2_{tr}=0.835,R^2_{test}=0.867<br>NL:<br>R^2_{tr}=0.893,R^2_{test}=0.876	Gong et al. (2008)[56]
Substituents do not have multiple bonds involving carbon atoms	L	-log(LC50),<br>fathead minnow	logP,EC(tot)/#atoms, WPSA3PM3, min Vc, Hacc	n=108, R^2=0.855,<br>R^2cv=0.831	Colombo et al.(2008)[31]
Benzenes substituted by conjugated alkenes, acids and their derivatives	L	-log(LC50),<br>fathead minnow	α, maxV_o, $RNCG_{PM3}$, -I_B	n=39; R^2=0.862,<br>R^2cv =0.825	Colombo et al.(2008)[31]
	L	-log(1/EC50),<br>D.magna	count of H-acceptor sites [Zefirov's PC], -N of aromatic bonds, -Balaban index, logP, -HA dependent HDCA-2/TMSA [Semi-MO PC]	n=130, R^2 =0.759,<br>R^2_{cv} = 0.728	Katritzky et al.(2009)[57]
	NL (DTB, SVM)	multiple species (T. pyriformis,<br>P. promelas,<br>P. reticulata, and<br>R. japonica),	SHdsCH, lipoaffinity index, TopoPSA, MW,nAtomP, ALogP2, CrippenLogP, XLogP	T. pyriformis, n=392,<br>SVM L-QSTR: R^2_{train} =0.897, R^2_{test}=0.896;<br>DTB L-QSTR: R^2_{train} =0.978, R^2_{test}=0.951;<br>All species:<br>G-QSTR models<br>SVM-QSTR R^2_{train} =0.791, R^2_{test}=0.846;<br>DTB-QSTR R^2_{train} =0.965, R^2_{test}=0.946;	Gupta et al.(2015)[58]
	L<br>Monte Carlo based	pIGC50,<br>T.pyriformis	Descriptors of Correlation Weights calculated with molecular features extracted from the SMILES	R^2 = 0.8179-0.8682,<br>n=286-299	Toropova et al. (2016)[59]

	L (MLR +GA)	log(1/IGC50), T. pyriformis	Descriptors from atom weighted vectors	n=392, R^2=0.837	Martinez-Lopez et al. (2017)[60]
Benzonitriles	L	-log(LC50), fathead minnow	φ, Qmin, $FNSA3_{PM3}$	n=10; R^2=0.994, R^2_{cv}=0.984;	Colombo et al.(2008)[31]
(Benzo)triazoles	L	pEC50, D.magna<br><br>pLC50, O.mykiss	-TPSA(NO), Aeigm, nCar, nHDon, H-052 (Dragon descriptors);<br><br>-TopoPSA, WPATH, C2SP2, - maxHaaCH, -nT9Ring (PaDEL-Descriptors)<br><br>CIC[1], Mp, H-052, -TPSA(tot) (Dragon descriptors);<br><br>VP-1, -SHBint2, - maxHaaCH (PaDEL-Descriptors)	Ntr=97, R^2=0.77, CCC_{ext}= 0.85–0.89<br>R^2=0.73, CCC_{ext}= 0.85–0.89<br><br>Ntr=75, R^2=0.79, CCC_{ext}= 0.92<br><br>R^2=0.76, CCC_{ext}= 0.82	Cassani et al.(2013)[61]
Triazoles and benzotriazoles	L	pEC50, D. magna<br><br>pLC50, O. mykiss<br><br>pEC50, P.subcapitata,	-TopoPSA, WPATH1, C2SP2, -maxHaaCH2, -nT9Ring<br><br>VP-1, nHBAcc, – minHBd,<br><br>SwHBa, WPOL, MDEN-22	n=97, R^2=0.73, CCC_{test}=0.85-0.89<br><br>n=75, R^2=0.76, CCC_{test}=0.86-0.88<br><br>n=35, R^2=0.82, CCC_{test}=0.88-0.89	Gramatica et al.(2014)[37]
Compounds with C=O group					
Benzoic acids & derivatives	L	log(1/EC50), P. subcapitata (microalgae),	logKow, $(N_{OH})^4$	n = 20, r^2 = 0.965, Q^2 = 0.955	Lee&Chen(2009)[62]
Substituted benzoic acids	L(11)	log(1/EC50) Different species	logP, S, I_{NO2} , $logF_0$<br>negative effect $logF_0$ for D.magna and carp, no effect or + for V. fischeri.	R^2=0.73-0.88	Qin et al.(2010)[63]
Aromatic aldehydes	L	log(1/IGC50), T. pyriformis	log Kow and Amax<br>-separetly 2-and or 4-hydroxylated aldehydes<br>-other aldehydes (similar to general benzene model)	n = 25, R^2 = 0.916, $R^2_{CV=}$ 0.896;<br>n=52, R^2=0.864, R^2_{CV}=0.844;	Netzeva& Shultz (2005)[64]
Aromatic aldehydes and ketones	L	-log(LC50), fathead minnow	logD7.4, min Etot(C-H),<br>-minVH, WNSA1Zefirov’s PC, -maxRc	n=44; R^2=0.870, R^2_{cv}=0.824	Colombo et al. (2008)[31]

Aromatic aldehydes	L	pIGC50,<br>T. pyriformis	Best ETA model: $[\eta' F]_8$, $[\eta' F]Cl$, $\sum\beta'$, $\sum\alpha$,<br>$[\eta' F]_{NO2}$, $\sum\varepsilon/N$	Q^2_{int}= 0.709, Q^2_{ext}= 0.744	Roy&Das(2010)[65]
Aromatic aldehydes	L, NL (ANN)	log(1/IGC50),<br>T. pyriformis	log Kow, $-\chi^1$ A, Ip<br>Ip is 1 if it is 2- and/or 4-hydroxylated aldehyde, otherwise 0	n_{tr}= 62, R^2_{adj} =0.891<br>n_{ext}=15, R_{ext}^2 =0.877<br><br>ANN: R^2=0.906,<br>R_{ext}^2 =0.902	Louis & Agrawal (2011)[66]
Aliphatic and aromatic aldehydes	L	-logLC50, fathead minnow	ClogP, -FSNA(Zefirov PC),<br>-HA dependent HDCA-1, ΔQ_{CO} NBO	n=50, R^2 = 0.868,<br>$R^2_{CV=}$ 0.840	Smiesko&Benfenati (2004)[34]
Aliphatic and aromatic aldehydes	L	pLC50,<br>mix of fishes	MlogP2, B08[C-C],<br>B02[C-C], -B05[C-C],<br>Fr5(elm)/C_C_C_H_O/1_2s, 2_3a, 3_5s, 4_5s/, F04[O-O]	n_{tr}=39,<br>n_{test}= 13,<br>R^2=0.840,<br>R^2_{pred}=0.860<br>CCC=0.920	Khan et al.(2019)[35]
Aliphatic and aromatic esters	L	log1/LC50, Pimephales Promelas<br><br>log1/EC50 Daphnia<br><br>log1/EC50 in algae	DRAGON descriptors<br>MAST4v, -REIG<br><br>-TIC0, -nCp, n=CH2<br><br>DISPp, H8u<br><br>ESter Aquatic Toxicity INdex (ESATIN):<br>-SHP2, n=CH2, DISPp	n_{tr}=24, n_{test}=6,<br>R^2_{adj}=0.823,<br>Q^2_{LOO} =0.785<br>Q^2_{EXT}=0.715<br>n_{tr}=24, n_{test}=5,<br>R^2_{adj}=0.860,<br>Q^2_{LOO} =0.831<br>Q^2_{EXT}=0.790<br>n_{obj}=11, R^2_{adj}=0.949,<br>Q^2_{LOO} =0.923<br>n_{tr}=31, n_{test}=30,<br>R^2_{adj}=0.898,<br>Q^2_{LOO} =0.873<br>Q^2_{EXT}=0.866	Papa et al.(2005)[36]
Nitrobenzene and aniline derivatives					
Nitrobenzenes	L	pC, T. pyriformis	AlogP98, $[\eta' F]_{NO2}$, $-[\eta' F]_{CH3}$, $[\eta' F]_{Br/I}$	n=42, Q2=0.892,<br>R^2_a=0.911	Roy&Ghosh(2004)[67]
Nitroaromatics	L	-lgEC50, algae S. obliguus	mononitro derivatives: logKow;<br>All: $-E_{LUMO}$, Q_{NO2} (the charge of the nitro group)	n=18, R=0.9044<br>n=22, R=0.926	Yan et al (2005)[68]
Substituted nitrobenzene and aniline compounds	L	logLC50<br>daphnia,carp,	daphnia: $-{}^3\chi_p$, ${}^5\chi_{pc}$, $-{}^4\chi^V_{pc}$<br><br>Carp: $-{}^7\chi_{p}$, $-{}^4\chi^V_{pc}$	n=18; r=0.856,<br>Q=0.757;	Lin et al.(2009)[69]

				n=16; r=0.898, Q=0.785	
Nitrobenzenes	L	log(1/IGC50), T. pyriformis	ω (Parr's electrophilicity index), Elumo, logP	n=50, R^2_{adj}=0.87.	Bellifa & Mekelleche(2016)[70]
Chlorinated anilines	L(3)	log EC50 P. subcapitata, D. magna, log LC50 D. rerio	logKow	n=4, R^2=0.932-0.998	Dom et al. (2010)[71]
Anilines	L	pLC50, mixture of fishes (Brachdanio rerio, Pimephales promelas, Cyprinus carpio, Oryzias latipes, Poecilia reticulata, Lepomis macrochirus, and Oncorhyncus mykiss)	ALogP2, MLOGP2, - T(N. .F), S_A(att)/E_E_E_F/1_4s, 2_3a/3, -H-051, Fr5(lip)/B_B_B_C_C/1_2s, 2_4a, 3_5a, 4_5a/, F02[C-N], F07[O-O]	n_{tr}= 70, n_{test}= 23, R^2= 0.700, R^2_{pred}=0.670	Khan et al.(2019)[35]
Phenols and phenol derivatives					
Phenol derivatives	L	LC50, R.japonica (tadpole)	logKow, -Elumo, HOF(heat of formation), $^1\chi^p$ (first order simple molecule connectivity index)	n=31, r^2=0.85	Wang et al. (2001)[72]
Phenols, MOA classification discrimi nant functions	NL	MOA, Tetrahymena pyriformis	pKa, -pKa2, logKow, -logD, logD2, -ELUMO, -ELUMO2	Accuracy 71-92%	Ren(2003)[73]
Substituted phenols (no other known reactive functional groups (e.g. carboxyl acids, aldehydes))	L	-logLC50 Pimephales, Tetrahymena	ClogP, -ΔH298gas	n=53, R^2=0.853, R^2_{CV}=0.834<br><br>n=45, R^2=0.864, R^2_{CV}=0.833	Smiesko&Benfenati (2005)[74]
Phenols	L, NL(CP ANN)	pT48, D. tertiolecta	MLR consensus model of 3: Mor24m+C_{ortho}, Mor24p+HATS7e, Mor18u+ C_{ortho} -k1max[a]<br>ANN model: C_{ortho}, Mor24p, Mor18u, $k_1$max[a]	N=24, MLR consensus: Q^2_{test}=0.94<br>ANN: Q^2_{test}=0.93	Ertürk et al.(2012)[75]
Phenol derivatives	L	log(1/IGC50), Tetrahymena pyriformis	MWC02, -MWC09, MWC10, -piPC02, piPC03, -piPC08, -TPC, X3A, -nCconj, nR = Cs, -nRCN, -nCXr, -O-059, -BLTD48	n=240, R^2=0.74	Dieguez-Santana et al. (2016)[76]

Phenols	NL (GA+ CART tree) +MLR	Tetrahymena pyriformis	Descriptors in Table 9<br>(MLR models at the leaves of the tree constructed using the GA-CART algorithm)	n=163, R^2_{tr} = 0.91, R^2_{pred}=0.93, q^2=0.89	Abbasitabar & Zare-Shahabadi(2017)[77]
Substituted phenols	L(4)	pT, C.vulgaris	pT; ATS2m,<br>pT: Mor09m, TDB04e,<br>pT: logP, -Hardness, Hardness is calculated as half of the ELUMO− EHOMOgap.<br>pT: logD, nHBonds, Tm,	n_{tr}=35, n_{pr}=11,<br>R^2_{tr}=0.67, R^2_{pr}=0.79 (2)<br>R^2_{tr}=0.79, R^2_{pr}=0.80 (3)<br>R^2_{tr}=0.61, R^2_{pr}=0.79(4).<br>R^2_{tr}=0.86, R^2_{pr}=0.94. (5)	Tugcu et al.(2017)[78]
Phenols	L	pLC50, mixture of fishes	-BLTA96, nCrs, -Fr5(lip)/B_C_C_C_C/1_3a, 2_3s, 3_5a, 4_5a/, nArNO2, N-069, nArCHO	n_{tr}=107, n_{test}=36,<br>R^2=0.760, R^2_{pred}=0.900	Khan et al.(2019)[35]
Phenol and aniline derivatives					
Phenols and anilines derivatives	L(4)	Several endpoints, C.vulgaris	pNOEC: SM04_EA(bo), E1m,<br>pNOEC: pIC50,<br>pIC20: T_Grav3, Mor09m,<br>pIC20: pIC50	n=48, n_{test}=12<br>R^2=0.634, , R^2_{test}=0.756<br>R^2=0.914, R^2_{test}=0.958<br>R^2=0.653, R^2_{test}=0.849<br>R^2=0.979, R^2_{test}=0.991	Tugcu &Saçan(2018)[79]
Substituted phenols and anilines	L	Several endpoints C.vulgaris	Uniform norm indexes	IC50: n=67, R^2_{tr}=0.885, R^2_{test}=0.955<br>IC20: n=67, R^2_{tr} =0.857, R^2_{test} =0.946<br>LOEC: n=67, R^2_{tr} =0.836, R^2_{test} =0.872<br>NOEC: n=60, R^2_{tr} =0.856, R^2_{test} =0.859	Yan et al.(2019)[80]
Phenol and aniline derivatives	L (4)	Several endpoints, algae C. vulgaris	pIC20: X2sol, B01[C-Cl], B03[N-O]<br>pIC50: ε2, -SaaCH, -T(Cl..Cl), MLOGP<br>pLOEC: X2sol, B01[C-Cl], B03[N-O]	n_{tr}=54, R^2_{adj}=0.625, n_{test}=13, Q^2_{F1-F2}=0.784-0.783<br>n_{train}=52, R^2_{adj}=0.681, n_{test}=15, Q^2_{F1-F2}=0.699-0.696<br>n_{tr}=54, R^2_{adj}=0.629, n_{test}=13, Q^2_{F1-F2}=0.753-0.752	Seth &Roy(2020)[18]

			pNOEC: Σα/Nv, ε1, η/Nv, -F03[O-CL], -F04[Cl-Cl],	n_{tr}=45, R^2_{adj}=0.686, n_{test}=15, Q^2_{F1-F2}=0.724-0.724	
Aniline (n=28) and phenol (n=30) derivatives	L	pEC50, P. subcapitata, V. fischeri	Tchebichef image moments	N_{tr}=45, N_{test}=13, Q^2>0.79, R^2_p>0.75	Muhire et al(2020)[81]
Aliphatic Cyclic/Polyaromatic					
Polyaromatic compounds	L	-log(LC50), fathead minnow	-(eHOMO-eLUMO), logP, W(size)	n=37, R^2=0.887, R^2cv=0.829	Colombo et al.(2008)[31]
Compounds containing at least one aliphatic ring	L	-log(LC50), fathead minnow	logP, XY, relative N of rings, -polarity parameter (the difference of the maximum and minimum atomic partial charge divided by the square of the distance between these two atoms ((q_{max} -q_{min})/r^2)	n=116, R^2=0.809, R^2cv=0.790	Colombo et al.(2008)[31]
Cyclic +polyaromatic compounds	L	-log(LC50), fathead minnow	logP, relative N of rings,XY	n=153, R^2=0.830, R^2cv=0.819	Colombo et al.(2008)[31]
Substituted arenes (halogenated aromatics)	L	-lgEC50, photoluminescent bacterium<br><br>-lgLC50, fathead minnow	D, logKow,<br>D, logKow, (D-connectivity index, based on Randic's branching degree index (mχ))	n=30, R^2=0.881(0.774)<br>n=20, R^2=0.939(0.903)	Feng et al.(2018)[82]
Other					
Aromatic compounds containing a nitro or cyano group	NL(6)	log(1/IGC50), T.pyriformis,	logKow, Amax	n=203, R^2=0.0.73-0.80<br>R^2pred=0.61-0.74	Ren(2003)[83]
Quaternary ammonium compounds					
	L	log(1/EC50), C. vulgaris	CL, αzz (polarizability tensor),- q+H (the most positive net atomic charges on a hydrogen atom), S°(entropy), MW, Qxx (values of quadrupole moment tensors in the x-coordinates), Eth (internal energy), ZPVE(zero-point vibrational energy), -V(molecular volume), logKow, C°V	n=11, R =0.975	Zhu et al. (2010)[84]
	L	-log(1/EC50), C. pyrenoidosa S. quadricauda	-chain lengths (CL),<br>-total connectivity (TCon)	n=13, R=0.877<br>n=12, R=0.949	Jing et al.(2012)[85]

Table S4. Application-based QSAR models

Applicability Domain	Models	Endpoint, species	Descriptors	Performance	Reference
			Pesticides		
	NL (GA/ CPANN)	LC50, rainbow trout	logP, $^{3}\chi^{V}_{p}$, BETA polarizability, HOMO-LUMO gap, HACA-2, HA dependent HDSA-1, FHBCA fractional HBSA	n=274, R^2=0.79	Mazzatorta et al.(2005)[86]
Aromatic pesticides	L	log (1/LC50), rainbow trout	CODE_MID (areas of the Van der Waals surface), MW, Heat of formation	n=96, R2 = 0.70	Slavov et al.(2008)[25]
	L	log (1/LC50), Daphnia magna	Combinations of topological parameters of molecular structures	n=220, R^2=0.78, n_{test} =42, R^2_{test}=0.74	Toropov&Benfenati (2006)[87]
	L (GA+ LDA classification)	LC50, several aquatic organism groups	Topological structural indices:<br>Slightly toxic vs highly toxic: MW, SddssS, -SHBd, Snitro, -Scarbony, SsSH,<br><br>Slightly toxic vs very highly toxic: MW, Xvp4, -ka1, -Xvp7, Snitroso, SddssS, SsSH, -SsBr, SdssS<br><br>Nontoxic vs highly toxic: MW, -sumI, -SssssNp, - SHCsats, - SHBint7, SddssS, -SHBint8, Hsulfuricacid<br><br>Toxic vs very highly toxic: MW, Xvpc4, sumI, SsCH3, SsOm, SHCsats	 n=392, Q_{cv}=71.4, Q_{ex}=66.3<br><br>n=389, Q_{cv}=79.1, Q_{ex}=79.8<br><br>n=573, Q_{cv}=79.7, Q_{ex}=80.6<br><br>n=423, Q_{cv}=72.7, Q_{ex}=76.7	Wang et al.(2010)[88]
Organothiophosphate pesticides	L	EC50, D.magna LC50, C.carpio	-log Kow, -Elumo(-Ehomo),	n=10, R^2=0.80-0.82, R^2_{ext} = 0.61-0.71	Zvinavashe et al.(2009)[89]
	NL, DTF (decision tree forest) DTB (decision tree boost))	-logEC50 (pEC50), algae S. capricornutum to build the model, external validation on other species (n=116), 33 pesticides in P. subcapitata, 12 in	six different descriptors (TPSA, dchi4, dchi0, GATSm8, Aweight, WPSA1)	DTF:<br>n = 116, R^2=0.895, Q, CCC validation: 0.833-0.921, global multispecies data n=253, R^2=0.890, R^2_{test}=0.841	Basant et al.(2015)[90]

		S. subspicatus, 19 in A. flos-aquae, 21 in N. pelliculosa, 17 in S. costatum, and 35 in L. gibba.		DTB: R^2=0.977, Q, CCC validation: 0.925-0.958, global: R^2=0.921, R^2_{test}=0.9	
	NL(6 ML algorithms, global and local models)	Fish rainbow trout (RT) and bluegill sunfish (LP, L. macrochirus) for local model Global models: 2 species fish +other fish species	Molecular fingerprints: Extended fingerprint (Ext), Estate fingerprint (Est), MACCS fingerprint (Maccs), PubChem fingerprint (Pub), Substructure fingerprint (Sub) Graphonly fingerprint (Graph), AP2D fingerprint (AP2D) and Klekota-Roth fingerprint (KR).	RT: n=829, Best local Maccs_ANN (accuracy=0.90) LP: n=151, Maccs_SVM (accuracy=0.90) Global: n=1258, Best: Graph_SVM (accuracy=0.89)	Li et al.(2017)[16]
	NL(classification, various ML models)	EC50, D.magna,	Molecular descriptors and fingerprints	n=639, best model Ext-SVM (Extended fingerprint - support vector machine) Q_{high}=0.807,$Q_{moderate}$=0.806, Q_{low}=0.755, Q_{total}=0.794), test set verification: Q_{high}=0.865, $Q_{moderate}$=0.783, Q_{low}=0.931, Q_{total}=0.848	He et al.(2019)[91]
	L	log(EC50), algae<br><br>log(EC50), D..magna<br><br>log(EC50), aquatic plants	- PubchemFP645 (O=C-N-C-C), - MATS4e, - PubchemFP346 (C (~C) (~H) (~O))<br><br>BCUTp-1h, - PubchemFP12(number of C atoms equal to or greater than 16).), - MIC0,<br><br>BCUTc1l, - maxwHBa<br><br>PubchemFP613 (C-N-C-C-C), - minaaCH, - SpMax5 Bhi,	n=28, R^2=0.82<br><br>n=29, R^2=0.75<br><br>n=13, R^2=0.94<br><br>n=12, R^2=0.96	Galimberti et al.(2020)[92]

		log(EC50), fish Pimephales promelas,<br><br>log(EC50), fish O. mykiss,	GATS6e, SRW5, - PubchemFP179(at least one or more saturated or aromatic carbon-only ring of size 6)	n=12, R^2=0.96	
	L	pLC50, rainbow trout (O.mykiss)	Norm descriptors, MW	n=311, R^2=0.8053, Q^2_{LOO}=0.7606	Jia et al. (2020)[93]
Biocides	L, NL	EC50, D.magna,	Linear regression: - piPC04, WiA_Dz(p), SpMax_B(p), - ATSC7m, ATSC6s, P_VSA_p_3, -P_VSA_ppp_D, Eig03_AEA(dm), C-005, - CATS2D 03 DA, - CATS2D 06 DA, - CATS2D 09 AA, - CATS2D 02 AN, - CATS2D 05 NL, CATS2D 06 NL, T(N..N), -B01[N-N], B02[N-S], B05[N-S], B09[C-C], -F05[C-N], -F05[N-S], VE1_D (Table 2)<br><br>Random forest descriptors: B01[C–S], B02[C–S], B09[N–O], F02[N–N], F05[C–N], F09[C–F], J_D/Dt, C-026, SpMax3_Bh(v), SpMax5_Bh(s), CATS2D_06_DA, CATS2D_04_AA, CATS2D_04_DA, CATS2D_05_DA, SpMax_EA(dm), nRCOOH, nROH, P_VSA_ppp_D, P_VSA_ppp_N, P_VSA_v_3, DBI<br>Table 3	linear regression (r^2_{tr}=0.83, r^2_{valid}=0.75)<br><br>Random forest (best, r^2_{tr}=0.97, r^2_{valid}=0.89, Monte Carlo (CORAL) (r^2_{tr}=0.74, r^2_{valid}=0.75), n=133	Marzo et al.(2020)[94]
	L	pLC50, rainbow trout	Monte Carlo method, model is improved by the index of ideality of correlation	n=311, R^2=0.81-0.86	Toropov et al.(2020)[95]
	L	log1/EC50, D.magna	Inert compounds(Baselines): logKow<br>Pesticides: logKow<br>Herbicides: logKow,<br>Fungicides: logKow,<br>Insecticides: logKow,<br>Baselines +Herbicides: logKow, F+<br>Baselines+Fungisides: logKow,Hf , PSA<br>Baselines+insecticides: logKow,-S,V,B,CV	n=25, R^2=0.90<br>n=57, R^2=0.35<br>n=9, R^2=0.88<br>n=8, R^2=0.61<br>n=40, R^2=0.28<br>n=34, R^2=0.92<br>n=33, R^2=0.82<br>n=65, R^2=0.80	Wang et al.(2020)[96]
	L	pLC50, Sheepshead minnow	Insecticides: GATS3e, - AATSC8v, MDEC, - n6HeteroRing, CrippenLogP, ns,<br>- mmUBint3	n_{tr}=81, n_{test}=26, R^2=0.817	Yang et al. (2020)[97]

			Herbicides:, - ndssC, - AATSC7m, ETA_Beta_ns_d, CrippenLogP, MDEN, AATSC3c, VPC–6<br><br>Fungicide: XLogP, AATS1m, - maxHCsats, VE2_D, JG17, - VE3_Dzs<br><br>Pesticides: ETA.dBeta, - nHBAcc, MATS2e, CrippenLogP, - ndO, ALogpz, VP–z, MDEO −12	n_{tr}=80, n_{test}=27, R^2=0.772<br><br>n_{tr}=58, n_{test}=20, R^2=0.813<br><br>n_{tr}=235, n_{test}=78, R^2=0.696	
	L	pLC50, Americamysis bahia	Herbicides: CrippenLogP, - maxHBint2, - MDEC – 13, MATS6e, - ETA_ Shape_X, VC – 3<br><br>Insecticides: -nssCH2, - n6HeteroRing, - minHBint2, ATSC6e, CrippenLogP , - 47J43VE2_DzZ, KE1 Dt.<br><br>Fungicides: -ZMIC4, - BCUTw – 1h, 47J43VE2_DzZ, KE1 Dt.C1, -minHBint2, SpMaxl_Bhp<br><br>Agrochemicals: XLogP,-ATS3e, -BCUTp – 11, VP – 2, MDEO -22, SCH – 6, -maxHBint2, - nHeteroRing	n_{tr}=77, n_{test}=25, R^2=0.821<br><br>n_{tr}=12, n_{test}=24, R^2=0.800<br><br>n_{tr}=44, n_{test}=15, R^2=0.838<br><br>n_{tr}=217, n_{test}=72, R^2=0.688,	Yang et al.(2020)[98]
Pharmaceuticals					
	L	log(1/EC50), V. fishery, D. magna	- logDow, -ΔE (EHOMO−ELUMO gap)<br><br>logDow		Kim et al. (2007)[99]
	NL (DTB, DTF)	pLC50, daphnia, algae, and fish.	7 descriptors for classification, and 9 for regression:<br>-LogS aqueous solubility<br>-chi0C Carbon connectivity index order 0<br>-SP-4 Chi simple path descriptor of order 4<br>-VP-0 Chi valence path descriptor of order 0<br>-VP-5 Chi valence path descriptor of order 5<br>-VPC-4 Chi valence path cluster descriptor of order 4	Classification DTB and DTF Accuracy training 99.22% n =129, test DTB 96.55% , DTF 89.66%<br>External validation on algae (n=64) and fish (n=130): 84.38%, 85.94% accuracy of	Singh et al. (2015)[100]

			-MDEC-23 Molecular distance edge between all secondary and tertiary carbon<br>-MDEO-11 Molecular distance edge between all primary oxygen<br>-ATSm2 The Moreau–Broto autocorrelation descriptor weighted by scaled atomic mass<br>-RPCS Relative positive charge surface area<br>-MOMI-X Moment of inertia along X axis<br>-XLogP Logarithmic form of octanol water partition coefficient based on the atom-type method<br>-nRotB Number of rotatable bonds<br>-nAP Number of atom in largest pi system<br>-nAA Number of aromatic atoms	classification in algae and 78.46% and 79.23% classification accuracy in fish<br>Regression DTB training R^2 = 0.865, test R^2=0.797,<br>DTF training R^2 = 0.895, test R^2=0.720<br>Validation : R2 of 0.534, 0.556 (algae), 0.620.637 (fish)	
	L	pEC50 (72h), P. subcapitata<br><br>pEC50(48h), D.magna<br><br>pLC50(96h), O. mykiss<br><br>pLC50(96h), P.promelas	- minHother, -VCH-6, piPC6, - VE3 Dt,<br><br>CrippenLogKow, −minHBint2, −SpMAD Dzs, −AATSC4i, C2SP3<br><br>ZMIC2, −maxHBint2, −HybRatio, AATSC0v<br><br>Kier2, AATS3v, −nHBAcc, SpMin7 Bhp	n=45, R^2=0.78<br><br>n=125, R^2 = 0.75<br><br>n= 55, R^2 = 0.78<br><br>n = 62, R^2 =0.80	Sangion&Gramatica (2016)[101]
	L	ATI (Aquatic toxicity index)<br>P. subcapitata<br>D.magna<br>P. promelas or O.mykiss	Crippen log P(logKow), SaaCH, - SHBint2,	n=706, R^2=0.81	Gramatica et al.(2018)[102]
	L, (single models and consensus modeling, 5 models)<br>PLS	P. subcapitata<br><br>D.magna	F08[O-O], -UNIP, B08[C-C], PW4, ALOGP2, -nR=Cs, F06[C-Cl], CATS2D_04_DD, piPC1, piPC4, piPC8<br><br>CrippenLogP, - RotBtFrac, ASP, F03[C-F], gmin, -CATS2D_04_NL, AVS_B(v), -	n_{tr}=53, n_{test}=16, R^2_{tr}=0.74, Q^2_t=0.85 (best single equation), Q^2=0.85 (best consensus)<br><br>n_{tr}=124, n_{test}=51, R^2_{tr}=0.75,	Khan et al.(2019)[103]

			B03[C -N], MLOGP2, - CATS2D_02_AA, - ETA_Shape_X, - ETA_EtaP_B, -hmax,	Q^2_t=0.71(single model), Q^2=0.72 (consensus)	
		O.mykiss	AATS2m, -maxHBd, CrippenlogP, -minsF, AATS2p, ATS0m, hmax, HyWi_B(m), -SM1_Dz(i), P_VSA_m_4, -Eig07_AEA(dm), MlogP, -SHBa, -SdsN, B01[C-X], Mp, RBN, NCL	n_{tr}=46, n_{test}=25, R^2_{tr}=0.81, Q^2_t=0.87(single models), Q^2=0.79-0.92(consensus test)	
		P. promelas:	S2K, B02[C-X], C%, AlogP, AlogP2, MlogP,	n_{tr}=80, n_{test}=28, R^2_{tr}=0.78, Q^2_t=0.80 (single models) Q^2=0.81 (consensus)	
	L	pLC50, Algae	nRNR2, F08(O-O), Fr5(chg)/B_C_C_D_D/1_2s; 1_5s; 3_4s; 3_5s/, Fr(rf)=B_B_B_B_C= 1_3s; 2_3s; 2_5s; 3_4a/, S_A(type)=/C.3 C.AR_ H_ N.3= 1 4s; 3 4s/4, MLogP2	n_{tr}=89, n_{test}=26, r^2=0.805, r^2_{pred}=0.690	Khan et al.(2019)[21]
		pLC50, D.magna	CrippenLogP, GATS1i, MLogP2, -minHBint2, -S_A(elm)/C_Cl_H_O/1_3s; 1_4s/4, CATS2D_06_AP, -Fr5 (chg)/A_A_B_B_D/1_5s,2_5s, 3_4s,4_5s/	n_{tr}=133, n_{test}=34, r^2=0.62, r^2_{pred}=0.72	
		pLC50, Fish	MlogP, -minHBd, Fr5(chg)=B_B_C_C_C/1_2s;2_3s; 2_4s;2_5s/, nR=Ct, S_A(chg)/B_B_D_D/1_2a;3_4a/3, Fr5(chg)/A_B_B_C_C/1_5s; 2_3a; 2_4s; 2_5s/, -DLS_05	n_{tr}=135, n_{test}=43, r^2=0.64, r^2_{pred}=0.70	
Drugs	L(5), NL(SVM, kNN)	pLC50, P.promelas	MDs selected by more than one model: Mor12s, nN, P_VSA_logP_3 and Ui. Mor12s is the signal-12 3D-MoRSE descriptor weighted by the intrinsic state	n=288 R^2_{tr}=0.42-0.92, R^2_{test}=0.48-0.91	Serra et al.(2020)[104]
Solvents					
Chemically heterogeneous	L	log(LC50), fish P.promelas,	-logP,-γ, ε, LUMO	n=141 R^2_p >0.6	Levet et al.(2013)[105]

solvents of different classes		Brachydanio rerio and Cyprinus carpio	-1.8 <logP<4.3, -2.7<LUMO<0.8 eV, 15<γ<50 dyn/cm and ε<45.		
Organic solvents	L	pEC50(48h), D.magna<br><br>pEC50(72h), algae	-logP, -γ (surface tension), ε (dielectric constant), -qmin (minimal atomic Mulliken charges),<br><br>-logP, LUMO,	n=115, R^2 val=0.689-0.752<br><br>n=51, R^2 val = 0.706-0.744	Levet et al.(2016)[106]
Biomass solvents	L	logLC50, Danio rerio zebrafish	-logP, -CV	n_{tr}=27, n_{test}=7, R^2_{adj}=0.948	Zuriaga et al.(2019)[107]
Surfactants					
Ethoxylated and propoxylated alcohols	L	D.magna, log(1/EC50)	logP, general narcosis model	n = 8, $r^2_{adj.}$=0.996	Roberts et al(2007)[108]
Various surfactants	L	log(EC50), V.fisheri, D.magna, S. capricornutum	-R(alkyl chain length), EO (degree of ethoxylation)	-	Lechuga et al(2016)[109]
Amine surfactants	NL(genetic function approximat ion (GFA))	D. magna, logEC50	N, -CL, η, ZPVE, $-{}^0\chi$, ${}^2\chi$, $-{}^2\delta^v$	n_{tr}=18, n_{ext}=2, R^2_{tr}=0.962, R^2_{cv}=0.794, R^2_{ext}=0.942	Liu et al.(2020)[110]
Other					
Personal care products	L	ATI (Aquatic toxicity index)	XlogP, Mp, TIC1	n=484, R^2=0.93	Gramatica et al.(2018)[102]
Contaminants of Emerging Concern (CECs)	L(6 +1 consensus best)	pLC50, D. japonica	5 models with descriptors: nArCO, -B03[C-C], -H-046, nBnz, MlogP,F05[O-Cl], O-059, AlogP, -N%, -F03[O-O]	n_{tr}=56, n_{test}=19, R^2_{adj} = 0.705-0.769, R^2_{pred} = 0.723-0.798, Consensus models performed better	Hossain&Roy(2018)[111]
CECs	L	pLC50, D. japonica	logKow, GATS7p, SpMaxA_G/D, CATS2D_08_DL, Mor31s	n_{tr}=47, n_{test}=8, R^2_{adj} = 0.787, R^2_{pred} = 0.891	Önlü &Saçan (2018)[112]

Table S5. MoA based QSAR classification or regression models

Applicability Domain	Models	Endpoint, species	Descriptors	Performance	Reference
Narcotic vs reactive	NL (Logistic regression, discriminant analysis)	fathead minnow	logKow, – S^{N}_{AV}, EHOMO	Error rate:<br>LR: 10.2%,<br>DA: 11.4%	Ren&Schultz (2002)[113]
	NL (SVM)	LC50 fathead minnow	12 autoMEP vectors, 5 Sterimol descriptors, logP(o/w), HDon, HAcc, TPSA, ASA, HOMO(PM3), LUMO-HOMO(PM3)	n=296<br>Precision around 70% CV	Michielan et al (2010)[114]
	NL(Bayesian classifier)	logLC50 fathead minnow	ECFP6 Fingerprints,<br>Descriptors:<br>Hydrophobic (3),<br>Structural (7),<br>Spatial (50),<br>Electronic (25)<br>Max in 1 model:17	Global: n=425, r^2=0.70, n_{test}=165, r^2=0.57<br>Consensus model by MOA (nonspecific MOA) n=425, r^2=0.81, n_{test}=165, r^2=0.67	Lozano et al.(2010)[115]
Non-polar and polar compounds<br><br>Non-polar, polar, ionizable, nitro, α, β-unsaturated carbonyl compounds	L	log(1/IGC50)<br>T. pyriformis	logP, S(Abraham polarity/polarizability descriptor)<br><br>logP, S, F_i (fraction of ionized form),log F_0(fraction of neutral form), INO2, I(indicator variable for α, β-unsaturated ketones and aldehydes)	n=428, r^2=0.90<br><br>n=925, r^2 =0.78	Su et al.(2012)[116]
6 MoA groups	NL (LDA, RF)	fathead minnow	LDA (TEST: most common: molecular fragment counts,autocorrelation, molecular distance edge, Burden eigenvalue, and walk and path count descriptors)<br>RF (Dragon: primarily fragment counts, autocorrelation	n=924<br>LDA: 75% tr, 25% valid<br>RF: 84.5 and 87.7%	Martin et al. (2013)[20]

			descriptors, and Burden eigenvalues		
Various MOA	NL (Bayesian network model)	aquatic invertebrates and fish	Most important for AChEI: SdsssP (phosphate group), J, GATS1 v; ETI(electron transport inhibition): SdssNp(N groups), Hmax, Qv; Iono/osmoregulatory/circulatory: Hmax, BELe3, SRW10, Qv. Narcosis: MAXDN, DELS, SRW10, xv2, xc4, SdsssP, SsCL, and SdssNP. Neurotoxicity: SsCl, MDEC34, SRW10, SsssCH acnt, BELe3. Reactivity: SdssNp	Accuracy (model precision) of 80%	Carriger et al. (2016)[117]
Non-polar vs other<br><br>Less reactive vs more reactive	NL (logistic regression (LR), Linear discriminant analysis(LDA))	fathead minnow	LR: logKow, log (1/LC50), IC1, MATS1s, SAacc<br><br>LR: P_VSA_MR3, nArOH, NdsCH, RPCG	n=220+109<br>LDA: 85.40% excess toxicity<br>LR: 95.60%<br><br>n=139+66<br>LDA: 80.65%<br>LR: 88.17%.	Ren et al.(2016)[118]
MOA+toxicity value	NL (MoA kNN (k=3), appropriate polyparameter target site models)	log LC50	Abraham solvation parameters for MOA prediction	MOA: precision 0.50-0.99<br>Toxicity prediction: RMSE=0.752	Boone&Toro(2019)[119]<br>Boone&Toro(2019)[120]
Verhaar schema MOA	unsupervised machine learning and graph theory (improved Louvain method), prediction by ensemble learning	LC50/EC50 (daphnia, algae, fish)	MACCS keys	n=155<br>Accuracy this study vs Ecosar<br>Class 1: 76% vs 96%,<br>Class 2:87%vs 100%,<br>Class 3: 70% vs 64%,<br>Class 4: 55% vs 47%,<br>Other: 60%,67%	Takata et al.(2020)[121]
Excess toxicity	L	-logLC50 fathead minnow	logKow, X1v, -R2e	n_{tr}=6,n_{valid}=18, r^2_{adj}=0.850	Wu et al.(2016)[122]

Narcosis					
	L	log(1/LC50), fathead minnow	ClogP, -LUMO, -RARS	n_{tr}=147, n_{test}=116, R^2 =0.95, Q^2_{LOO}=0.95, Q^2_{EXT}=0.93	Papa et al. (2005)[123]
	L	-logLC50 fathead minnow	logKow, HyWi_B(m)	n_{tr}=696,n_{valid}=173, r^2_{adj}=0.762	Wu et al.(2016)[122]
Non-polar					
	L	log(1/IC50), D. magna<br><br>log(1/LC50), V. fscheri<br>log(1/LC50), P. promelas	α, -Ca<br><br>α, -Ca<br><br>α, -Ca	n=23 r^2=0.866<br><br>n=33 r^2=0.925<br><br>n =23 r^2=0.965	Dearden et al.(2000)[124]
	L	logLC50, guppy	-α ,∑Ca	n=90; r^2=0.953;	Raevsky&Dearden (2004)[125]
	L	logLC50, Poecilia reticulata (fish, guppy)	-logP, -DPSA-3 difference in CPSAs (PPSA3-PNSA3) [Zefirov's PC]	R^2_{cv}=0.9520<br>R^2_{valid}=0.9552	Katritzky et al. (2001)[126]
	L	log(1/EC50), V. fischeri	logKow	n=179,<br>R^2=0.94	Klopman&<br>Stuart (2003)[127]
	L	log(1/IGC50), T. pyriformis<br><br>log(1/LC50), P. promelas	logKow, -ELUMO	n=411, R^2=0.890<br><br>n=213, R^2=0.906	Dimitrov et al.(2003)[128]
	L, NL<br>GC+classficiation<br>PLS+NN	log (1/LC50), Pimephales promelas	diatomic fragments, LUMO like, H+	n=114<br>PLS: r^2=0.98<br>NN: r^2=0.97	Casalegno et al.(2005)[129]
	L	log(1/LC50), fathead minnow	ClogP, -LUMO, -RARS	$n_{training}$ =147, n_{test}=116, R^2=0.95, Q^2_{LOO}=0.95, Q^2_{BOOT} =0.94, Q^2_{EXT}=0.93	Papa et a.(2005)[123]
	L	log1/EC50, P. subcapitata	logKow	DO: n=26, r^2=0.94<br>GR: n=26, r^2=0.943	Hsieh et al.(2006)[130]
	L	log(1/IGC50) T. pyriformis	logP<br>0.92<logP<4.50	n=87, r^2= 0.96,	Ellison et al.(2008)[7]

	L	log(1/LC50), fathead minnow<br><br><br>log(1/LC50), rainbow trout	log Kow based models<br><br>molecular polarisability α (as a volume-related term) and the H-bond acceptor factor (∑Ca) model	n=53, n_{test}=13, R^2=0.927, R^2_{test}=0.937<br>n=53, n_{test}=13, R^2=0.929, R^2_{test}=0.958<br>n=25, n_{test}=6, R^2=0.925, R^2_{test}=0.949<br>n=25, n_{test}=6, R^2=0.949, R^2_{test}=0.923	Raevsky et al.(2008)[131]
	L	logLC50, guppy	log Kow based model<br><br>molecular polarisability α (as a volume-related term) and the H-bond acceptor factor (∑Ca) model	n=72, n_{test}=18, R^2=0.947, R^2_{test}=0.954<br><br>n=90, R^2=0.953	Raevsky et al.(2008)[131]<br><br>Raevsky&Dearden (2004)[125]
	L	log(1/LC50), Guppy+Fathead minnow+Rainbow trout	log Kow based<br><br><br>models with α, -∑Ca	n=153, n_{test}=33, R^2=0.929, R^2_{test}=0.968<br><br>n=153, n_{test}=33, R^2=0.943, R^2_{test}=0.964	Raevsky et al.(2008)[131]
	L	log(1/LC50), guppy	$logP_{ex}$ or<br>-∑Ca, chi1v ,<br>Hn =(HOMO-LUMO)/2	n=90, R^2=0.957<br>n=90, R^2=0.967	Raevsky et al.(2009)[132]
	L	log(1/EC50), different species	logP	D.magna:<br>n=25, r^2=0.90<br>Carp: n=7, r^2=0.92	Qin et al.(2010)[63]
	L	logLC50, D.magna	-logKow	n=60, r^2=0.93, q^2_{cv}=0,92	Kuhne et al (2013)[133]
	L	log(1/EC50),algae (P. subcapitata)	logKow-mix	n=50, R^2=0.9469, R^2_{cv}=0.9426	Aruoja et al. (2014)[134]
	L	-log(LC50),<br>P. promelas	E (nAB, -nH, nHdon, -CEE1, -ELUMO, Mw)<br>-S(-nAB, nHacc, μ, Mν, -nF,-WNSA, Nν)<br>-B-basicity (nHacc, nN, nH,-Mlogp, Mν,α, ELUMO)<br>V-McGowan volume.	n_{train} =107, n_{EXT}=26,, R^2_{adj} = 0.902; R^2_{EXT} = 0.854	Lyakurwa et al.(2014)[135]

	L	logLC50, mixed species, majority O.mykiss	-logKow	n=107, r^2_{adj}=0.91, r^2_{ext}=0.90	Austin et al.(2015)[136]
Baseline compounds	L	log 1/LC50(AFT)<br>log 1/LC50(ZFET)	logKow,	n = 147 R^2 =0.95<br>n = 25 R^2 =0.91	Zhu et al.(2018)[137]
	L	log(1/EC50), algae P.subcapitata	logKow, - MATS7i, - τ x 103 , - MATS3p, TPSA(Tot), -Vs	n=67, R^2=0.829	Bakire et al.(2018)[138]
	L<br>(PLS)	-logEC50, fish	19 Volsurf descriptors: D1, D2, HAS, R, FLEX, V, S, POL, G, MW, D5, W1, CD6, D6, CD7, CD8, -D7, D8, ID3<br>(Table S4)	n=25, R^2 = 0.823, Q^2cv = 0.793, Q^2ext = 0.87	de Morais e Silva et al(2018)[139]
	L	log(1/HC) hazardous concentration for aquatic communities (5%)	logKow	n=28, R^2adj =0.97	Finizio et al.(2020)[14]
	L, NL (SVM)	Vibrio fischeri, log1/IBC50 (50% inhibition concentration),	dragon descriptors<br>logKow, SpDiam AEA(ed), N%,<br>-O−057, - B09[C−Cl], Eig04_AEA(ed), - GGI3	Linear models: n_{tr}=172, R^2_{adj}=0.778, n_{ext}=43, R^2_{ext}=0.788.<br>Nonlinear models: R^2=0.814, R^2e_{xt}=0.792	Zhang et al.(2020)[140]
Polar					
	L	log(1/LC50), P. promelas<br><br>log(1/IC50), D. magna<br><br>log(1/LC50), V. fscheri	α, -Ca<br><br>logP<br><br>logP, -Vx or α and Ca	n =10, r^2 = 0.877<br><br>n =12, r^2 = 0.627<br><br>n =15, r^2 = 0.791	Dearden et al.(2000)[124]
	L	log(1/LC50), guppy<br>log(1/LC50), fathead minnow	α, -∑Ca, -∑Cd	n=119, R^2=0.895,<br>n=50, R^2=0.883,	Raevsky et al.(2009)[132]
	L	log LC50, Poecilia reticulata (fish, guppy)	-logPc, - RNCG relative negative charge	R^2_{cv}=0.9083<br>R^2_{valid}=0.9083	Katritzky et al.(2001)[126]

			(QMNEG/QTMINUS) [semi-MO PC],<br>-FHDCA fractional HDCA (HDCA/TMSA) [semi-MO PC],<br>- XY shadow, YZ shadow		
Di<br>and Trihydroxybenzenes: -OH group in meta position, oxidizing to electrophilic quinones or quinone methides	L	pIGC50,<br>T.pyriformis	log D	n=10, r^2_{adj}=0.981,<br>q^2=0.974	Aptula et al.(2005)[141]
	L	log(1/LC50),<br>fathead minnow	AlogP, BEHv3, nHDon,<br>-C-029<br><br><br>logPfree:<br>C-002, BEHm3, nBnz, -nN	$n_{training}$ =57, n_{test}=29,<br>R^2=0.90, Q^2_{LOO}=0.88,<br>Q^2_{BOOT} =0.80,<br>Q^2_{EXT}=0.89<br>$n_{training}$ =57, n_{test}=29,<br>R^2=0.84, Q^2_{LOO}=0.81,<br>Q^2_{BOOT} =0.88,<br>Q^2_{EXT}=0.84	Papa et a.(2005)[123]
	L, NL<br>(GC, PLS, NN)	log (1/LC50),<br>Pimephales promelas,	diatomic fragments, LUMO like, H+	n=76<br>PLS: r^2=0.86<br>NN: r^2=0.84	Casalegno et al.(2005)[129]
Based on substituted anilines	L	log 1/EC50, P. subcapitata	-ELUMO, log Kow	r^2=0.88, Q^2=0.817	Chen et al.(2007)[142]
	L	log(1/LC50),<br>guppy	logPex, -ESC<br><br>-Nv1, ESM, logPex	n=121, R^2=0.895<br><br>n=121, R^2=0.904	Raevsky et al.(2009)[132]
Phenols and anilines based	L	log(1/EC50),<br>different species,	log P	V. fischeri:<br>n=15, r^2=0.84<br>D.magna:<br>n=25, r^2=0.58<br>Algae:<br>n=13, r^2=0.76<br>Fathead minnow: n=16, r^2=0.64<br>Guppy:<br>n=20, r^2=0.81	Qin et al.(2010)[63]
	L	-log(LC50),<br>P. promelas	-S(-nAB, nHacc, μ, Mv, -nF,-WNSA-3, Nv)	n_{train} =75, n_{EXT}=19, R^2_{adj} = 0.880; R^2_{EXT} = 0.861	Lyakurwa et al.(2014)[135]

			-B-basicity (nHacc, nN, nH,-Mlogp, Mν,α, ELUMO)<br>A-acidity (-SAdon, -nAdon,-nHdon,Mν,μ, -HACA-1, -nO)<br>V-McGowan volume		
	L	log(1/EC50),algae (P. subcapitata)	logKow, ΔHf/#atoms, MW	n=87, n_v=21, R^2=0.9149, R^2_{cv}=0.9061<br>R^2_{ext}=0.9241	Aruoja et al. (2014)[134]
	L	log(1/EC50), P.subcapitata	logKow, -Vsmin, q-C , G3p, α, - Mor32i, Vsmax	n=61, R^2=0.827	Bakire et al.(2018)[138]
	L, NL (SVM)	log1/IBC50 (50% inhibition concentration), Vibrio fischeri	EE_B(p), logKow, −X2v, −Eig03_EA(dm), - JGI3, CATS2D_05_DP, GATS7e	n_{tr}=133, R^2_{adj}=0.723, n_{ext}=33, R^2_{ext}=0.758<br>Nonlinear models: R^2=0.785, R^2_{ext}=0.705	Zhang et al.(2020)[140]
Less inert compounds	L	log 1/LC50(AFT)<br>log /LC50(ZFET)	logKow	n = 84 R^2 =0.84<br>n = 24 R^2 =0.73	Zhu et al.(2018)[137]
Non-polar + polar					
	L	logLC50, guppy Poecilia reticulata	-logPc, max sigma-sigma bond order, -average information content (order 1), - HA dependent HDSA-2 [semi-MO PC], - molecular volume/XYZ box, HACA-1/TMSA [Zefirov's PC]	R^2_{cv}=0.9340<br>R^2_{valid}=0.9362	Katritzky et al. (2001)[126]
	L	log(1/LC50), fathead minnow	logBCF, ELUMO	-	Dimitrov et al.(2002)[143]
	L	log(1/EC50), 5 min, V. fischeri	Vx, - $\sum\beta^H$	n=39, r_{adj}=0.91	Ren&Frymier (2002)[144]
	L	logLC50, guppy	L: -α, ∑Ca, ∑Cd (H-bond donor factor)	n=211; r^2=0.873	Raevsky&Dearden (2004)[125]
Non-polar+polar	L, NL<br>(GC, PLS, NN)	log (1/LC50), Pimephales promelas,	diatomic fragments, LUMO like, H+	n=190<br>PLS: r^2=0.95<br>NN: r^2=0.96	Casalegno et al.(2005)[129]

	L	log(1/EC50), V. fischeri, algae<br>log 1/IC50 D.magna<br>log 1/LC50, fish	log P, S(polarity)	V. fischeri:<br>n=48, r^2=0.86<br>D.magna:<br>n=50, r^2=0.73<br>Fathead minnow: n=44, r^2=0.87<br>Guppy:<br>n=45, r^2=0.90	Qin et al.(2010)[63]
Narcotic, reactive and ionizable compounds.	L	log(1/EC50),<br>V. fischeri,<br>log 1/IC50 bacteria,<br>D.magna, algae<br>log 1/LC50, fish	V. fischeri, bacteria, algae, fathead minnow, guppy : logP, S, I_{NO2}<br><br>D.magna, carp: logP, S, I_{NO2}, log Fo(fraction of neutral form)	V. fischeri: n=9, r^2=0.81<br>Bacteria: n=24, r^2=0.74<br>Algae: n=40, r^2=0.85<br>D.magna: n=100, r^2=0.73<br>Carp: n=46, r^2=0.88<br>F.minnow: n=82, r^2=0.84<br>Guppy: n=72, r2=0.82<br>Fish (carp, minnow, guppy): n=188, r^2=0.82	Qin et al.(2010)[63]
Nonpolar, polar and ionisable chemicals and their mixtures	L	log(1/EC50),<br>Aliivibrio fischeri	logKlipw<br>(0.5 < log Klipw < 4.3)	n=19, r^2=0.893	Escher et al. (2017)[145]
	L	log (1/IGC50),<br>T. pyriformis	logP, S (Abraham polarity/polarizability descriptor),	n=530, r^2=0.86	Su et al.(2012)[116]
	L	logLC50,<br>D.magna	B°, -E, -V	n=169, r=0.88, q^2_{cv}=0.87	Kuhne et al. (2013)[133]
	L	log(1/EC50),<br>algae P. subcapitata	logKow, -ELUMO	n=87, n_v=21, R^2=0.8532, R^2_{cv}=0.8449, R^2_{ext}=0.9241	Aruoja et al. (2014)[134]
Non-polar and polar compounds that were neutral at pH7	L	log (1/LC50), fish embryo acute toxicity	log Klipw	n=14, R^2=0.97	Klüver et al.(2016)[146]
Reactive					
	L	logLC50, Poecilia reticulata (fish, guppy)	FPSA-1 fractional PPSA (PPSA-1/TMSA) [Zefirov's PC], -number of single bonds, -final heat of formation/# of atoms, -	R^2_{cv}=0.8201<br>R^2_{valid}=0.8286	Katritzky et al. (2001)[126]

			average information content (order 0),<br>-min partial charge (Qmin) [Zefirov's PC]		
	L	log(1/LC50), fathead minnow	AlogP, -H6v, L1m, BEHm7, -R2u+<br><br>logP-free: Ss,-nHAcc, Tm, -GGI8,-HATS6u, ROR	$n_{training}$ =62, n_{test}=19, R^2=0.76, Q^2_{LOO}=0.70, Q^2_{BOOT} =0.69, Q^2_{EXT}=0.75<br>$n_{training}$ =62, n_{test}=19, R^2=0.82, Q^2_{LOO}=0.78, Q^2_{BOOT} =0.77, Q^2_{EXT}=0.77	Papa et a.(2005)[123]
	NL evolutionary algorithms (EAs) for optimizing neural and rule-based classifiers	P. promelas<br>T.pyriformis	logKow, Ehomo, Elumo, average acceptor superdelocalizability S'av	n=88, mean performance NN:80-83%, Rules: 90-93%	Fogel &Cheung (2005)[147]
	L	log(1/LC50), guppy	-CICO, IDWav, logPcalc	n=90, R^2=0.765	Raevsky et al.(2009)[132]
	L	-log(LC50),<br>P. promelas	nN=0, nK=0:<br>-A-acidity (-SAdon, -nAdon,-nHdon,Mν,μ, -HACA-1, -nO)<br>-B-basicity (nHacc, nN, nH,-Mlogp, Mν,α, ELUMO)<br>V-McGowan volume.<br><br>nN>0:<br>E (nAB, -nH, nHdon, -CEE1, -ELUMO, Mw)<br>V-McGowan volume.	n_{train}=35, n_{EXT}=9, R^2_{adj} = 0.843,<br>R^2_{EXT} = 0.835<br><br>n_{train} =33, n_{EXT}=7, R^2_{adj} = 0.803, R^2_{EXT} = 0.719	Lyakurwa et al.(2014)[135]
	L	log(1/EC50), P.subcapitata	nN=0, n(C=O)=0: logKow<br><br>nN>0 : GATS1e, SpMin1_Bh(p),<br>-V+s	n=9, R^2=0.798<br><br>n=10, R^2=0.891	Bakire et al.(2018)[138]

	L, NL (SVM)	log1/IBC50,<br>V. fischeri	nN = 0, n(C=O) = 0: B03[C−C], -Hy, B06[C-Cl], - B06[O−F]<br><br>nN > 0: X5Av, - MATS3s, GATS8m, - Eig11_EA(dm) -F06[C-O], MlogP<br><br>nN = 0, n(C=O) > 0: nRCO, -nArOR, SpPosA A, - Eta_F_A, F02[O-Cl], Eig02_EA(dm), Eig06_EA(dm),	n_{tr}=29, R^2_{adj}=0.841, n_{ext}=7, R^2_{ext}=0.833<br>Nonlinear models:<br>R^2=0.866, R^2_{ext}=0.790<br><br>L: n_{tr}=57, R^2_{adj}=0.777, n_{ext}=14, R^2_{ext}=0.738<br>NL:R^2=0.816, R^2_{ext}=0.801<br><br>L: n_{tr}=69, R^2_{adj}=0.704, n_{ext}=18, R^2_{ext}=0.447,<br>Nonlinear models:<br>R^2=0.736, R^2_{ext}=0.445	Zhang et al.(2020)[140]
Specifically acting					
	L	logLC50, guppy<br>P. reticulata	- ALFA polarizability (DIP) - FNSA-3 fractional PNSA (PNSA-3/TMSA) [semi-MO PC] + count of H-donors sites [Zefirov's PC] + number of benzene rings	R^2_{cv}=0.7745<br>R^2_{valid}=0.6740	Katritzky et al. (2001)[126]
	L	log(1/LC50), fathead minnow	ClogP, N-074, MPC09<br><br>lopP -free:<br>S1K, -MAXDN, -O-058, R2p	$n_{training}$ =29, n_{test}=7, R^2=0.78, Q^2_{LOO}=0.73, Q^2_{BOOT} =0.63, Q^2_{EXT}=0.91<br><br>$n_{training}$ =29, n_{test}=7, R^2=0.82, Q^2_{LOO}=0.72, Q^2_{BOOT} =0.67, Q^2_{EXT}=0.75	Papa et al.(2005)[123]
	L	log(1/LC50), guppy	Eamax, -∑Ca, Nv1	n=31, R^2=0.771	Raevsky et al.(2009)[132]
	L	log(1/EC50), P.subcapitata	- R6v, GATS3i	n=8, R^2=0.925	Bakire et al.(2018)[138]
	L, NL (SVM)	log1/IBC50, Vibrio fischeri	dragon descriptors: SpMAD_AEA(dm), -F03[C-N], SpMAD_B(p),	L: n_{tr}=25, R^2_{adj}=0.733, n_{ext}=6, R^2_{ext}=0.749	Zhang et al.(2020)[140]

				NL: R^2=0.799, R^2_{ext}=0.767	
	L,NL (LDA(2) kNN(4), SVM(4), ANN(4))	LC50, D.magna,	HYBOT (26) and DNESTR (19)	n=443<br>LDA: 0.864-0.886<br>kNN; 0.886 to 0.920,<br>SVM; 0.875 to 0.920,<br>for ANN, respectively (descriptors from 19 to 77)<br>Consensus kNN+SVM, ANN 77 descriptors<br>Acc_{tr}=0.915,<br>Acc_{test}=0.932	Grigorev et al (2014)[148]
Inhibitors of the Hill reaction of chloroplasts (phenylureas, triazines)	L	log(1/EC50) Chlorella	Phenylureas: η_2m, -As, logKow<br>Best 3 dimensional<br>Other models: η2m, As, logKow, nCl, Ku, IED, η2v, η2p, W,Tu,ZM2, MAXDN, $^1\chi$<br><br>Triazines: ^{2}k, η_1v, η_1p	n=15, R^2= 89.3%<br>Q^2_{LOO}=80.6%	Gramatica et al.(2001)[149]
pro-Michael acceptor electrophiles: Di and Trihydroxybenzenes, hydroxy groups oriented ortho or para to one another	L	pIGC50, T.pyriformis	-AEI (activation energy index)	n=18, r^2=0.821, r^2_{adj}=0.810, q^2=0.774	Aptula et al.(2005)[141]
Michael acceptors (α,β-unsaturated compounds)	L	log (1/IGC50), T. pyriformis	Max (Baseline toxicity BT; Reactive toxicity RT),<br>BT: 0.78logP-2.01<br>RT: log k (kinetic rate), logP	n = 94, r^2_{adj} = 0.85, r^2_{CV} = 0.83	Schwöbel et al. (2011)[150]
Endocrine disruptor chemicals (EDCs)	L	pEC50, P. subcapitata<br><br>pEC50, D. magna<br><br><br>pLC50, O. mykiss<br><br>pEC50, P. promelas	NaasC, -GD, - B06[C-O], -F-084, - ETA_Eta_ B, Cl-087<br><br>AlogP2, nCIC, P-117, -X1A, D/Dtr03,<br><br>-GD, XlogP, AlogP2, - SssCH2, B05[C-P], nCrt, - B09[O-Cl],<br><br>-MW, - ETA_BetaP_s	n_{tr}=61, n_{test}=16, Q^2=0.67<br><br>n_{tr} =81, n_{test} =24, Q^2=0.87<br><br>n_{tr} =83, n_{test} =36, Q^2=0.55<br><br><br>n_{tr} =12, n_{test}=4, Q^2=0.96	Khan et al.(2019)[151]

Unknown MoA					
	L	-log(LC50),<br>P. promelas	nN=0, nK=0:<br>E (nAB, -nH, nHdon, -CEE1, -ELUMO, Mw)<br>-B-basicity (nHacc, nN, nH,-Mlogp, Mν,α, ELUMO)<br>V-McGowan volume.<br><br>nN>0:<br>E (nAB, -nH, nHdon, -CEE1, -ELUMO, Mw)<br>-A-acidity (-SAdon, -nAdon,-nHdon,Mν,μ, -HACA-1, -nO)<br>-B-basicity (nHacc, nN, nH,-Mlogp, Mν,α, ELUMO)<br>V-McGowan volume	n_{train} =96, n_{EXT}=21, R^2_{adj} = 0.800; R^2_{EXT} = 0.836<br><br>n_{train} =114, n_{EXT}=27, R^2_{adj} = 0.748; R^2_{EXT} = 0.741	Lyakurwa et al.(2014)[135]
	L	log(1/EC50),<br>P.subcapitata	nN>0: α, logKow, -GGI9, DLS_02, - SpMAD_AEA(dm), - Mor10e, -Gli<br><br>nN=0, n(C=O)=0: EHOMO, - τx 103, -Vsmax, - HATS1i,	n=73, R^2=0.613<br><br>n=20, R^2=0.818	Bakire et al.(2018)[138]
Ester narcosis					
	L	log(1/IGC50),<br>T. pyriformis	logKow, -ELUMO (85% significance)	n = 93; R^2 = 0.878;	Dimitrov et al.(2003)[128]
	L	log(1/LC50),<br>P. promelas	logKow, A_{Ester}	n=34; R^2 =0.811;	Dimitrov et al.(2003)[128]
Amine narcosis					
Aliphatic amines	L	log(1/IGC50),<br>T. pyriformis	logKow	n=51; R^2 =0.854;	Dimitrov et al.(2003)[128]
Aliphatic amines	L	log(1/LC50),<br>P. promelas	logKow	n=88; R^2=0.853;	Dimitrov et al.(2003)[128]

Table S6. Global QSAR models

Applicability Domain	Models	Endpoint, species	Descriptors	Performance	Reference
	L	1/EC50 D.magna	logPow, Ha(hardness)	n=61, r2=0.54	Faucon et al. (2001)[152]
	L	log(1/EC50)<br>D. magna	atomic or group fragments and structural features	n=217, R^2=0.969	Tao et al. (2002)[153]
Narcotic chemicals	L	log(1/LC50), fish	logKow, SASurf PNSA1	n=216, r^2=0.892	Stanton et al. (2002)[13]
	NL (unsupervised for clustering + supervised ANN for model creation)	logLC50 P. promelas, logIGC50 T.pyriformis	156 descriptors	n=568, R_{2test} =0.872<br><br>n=724, R_{2test}=0.846	Gini et al.(2004)[154]
	NL (hybrid :linear (logP)+SANN)	LC50 fathead minnow	Autocorrelation descriptors	n=569(484+85), $RMSR_{tr}$=0.818, $RMST_{test}$=0.664	Devillers(2005)[155]
General,independent of MOA	L	log(1/LC50), fathead minnow	AlogP, DP03, H8m, -GATS1v, -R1v<br><br>logPfree: WA, Mv, H-046, nCb, MAXDP, -nN	$n_{training}$ =249, n_{test}=200, R^2=0.81, Q^2_{LOO}=0.80, Q^2_{BOOT} =0.80, Q^2_{EXT}=0.72<br>$n_{training}$ =249, n_{test}=200, R^2=0.79, Q^2_{LOO}=0.78, Q^2_{BOOT} =0.78, Q^2_{EXT}=0.71	Papa et al. (2005)[123]
Diverse chemicals	NL (Support vector inductive logic programming (SVILP))	pLC50, fathead minnow	Chemical fragments, logP, LUMO	n =576<br>R^2_{CV}=0.66, classification 73% accuracy,<br>R^2_{test}=0.57 on 165 unseen molecules	Amini et al (2007)[156]
	L (Abraham model, PCA)	-logIGC50 different protozoa	E,S,A,B,V	E. sulcantum n=51, R^2 =0.919,<br>U. parduczi n=59, R^2 =0.923,<br>C. paramecium: n=55, R^2 =0.887	Bowen et al.(2006)[157]
	L	-log(LC50) fathead minnow	AlogP, -ELUMO, S2K, nRNH2	n = 408 r^2 = 80.3 Q^2_{LOO} = 80.1 $Q^2_{Boostrap}$ = 80.0 Q^2_{ext} = 72.1	Pavan et al.(2006)[158]

	NL(LR, DT, k-NN,PNN,SVM)	log(1/IGC50), Tetrahymena pyriformis	Descriptors in Table 5 (48 descriptors)	n=841 TPT and 288 non-TPT, overall accuracies in the range of 85.3%~90.4% with SVM, k-NN, and PNN giving better performance.	Xue et al(2006)[159]
	L(6) , NL(13)	pIGC50, T.pyriformis	Various descriptors	n_tr = 644, n_val = 339 SVMDragon and ASNN approaches (0.83 and 0.75, respectively), ASNN had a better balance between the space coverage and accuracy Consensus models superior R^2_{val}=0.68-0.88, coverage=20-99%	Zhu et al.(2008)[160]
	L	-log(LC50) fathead minnow	first principle descriptors $-S_{tr}$, ω_H, $-\omega_L$, $-I_A$, -ClogP	n=45, R^2=0.85, R^2_{CV}=0.79	Eroglu et al(2007)[161]
	L, NL (Ensemble of linear and nonlinear)	logLC50 fathead minnow	Dragon descriptors	Regression nonlinear 8 descriptors r_{tr}^2 = 0.82 clustered ensembles, N_{tr}=560, N_{test}= 144, ensembles of linear models (e.g. 200 10-descriptor models in ensemble: r^2 =0.87, or for 200 simpler models having 7-descriptor models in ensemble r^2 = 0.83) clustered ensembles outperform linear and nonlinear MR ensembles. R^2=0.83-0.87	Basic et al.(2009)[162]
	L	logLC50 fathead minnow	-logPow, -MW, Elumo	n=566, r^2=0.65 Consensus 10 linear models: n=557, r^2=0.74, n_{test}=201, r^2=0.60	Lozano et al.(2010)[115]

	NL (GA-SVR)	-log LC50 fathead minnow	ALogP, $N_{N,rel}$, N_{ring}, φ, ε_{HOMO}, ε_{HOMO} , $E_{A,C,avg}$, $R_{A,C,avg}$	n=457, r^2_{tr}=0.826, r^2_{test}=0.802	Wang et al.(2010)[88]
	NL (SVM and ANN classification models)	LC50, fathead minnow (FMT),	60 selected descriptors, 6 most important: λ^{VDW}_{H5}, MB-ATS_2(ALOGP), Iα (4.0), H3, λ^{α}_{H5}, $ATS_{2,W}$	n =442(FMT), 169 (non-FMT) External validation set: prediction accuracy SVM: 90% for FMT, 100% non-FMT, 91.6% all ; ANN: 90,100 and 91.6%	Tan et al.(2010)[8]
	L	LC50 D. magna	-$logP_{mix}$, -HOMO energy, -WNSA-1,- BIC	n=118, r^2=0.7396, r^2_{cv} =0.7138, r^2_{scr} =0.0342	Moosus&Maran(2011)[163]
	NL, NL+L MLR , ANN, recursive partitioning (RP, grouping reactive or narcosis)+MLR	LC50 fathead minnow	8 constitutional descriptors, 12 geometrical descriptors, 1 physicochemical descriptor, and 207 topological descriptors.	n=555 (445+110) MLR, ANN, and two RP-MLR models possessed correlation coefficients (R^2) as 0.553, 0.618, 0.632, and 0.605 on test set<br>Consensus model of ANN and two RP-MLR models R^2=0.663	In et al.(2012)[164]
	L(6) , NL (3,spline)	-log(LC50) fathead minnow	ETA indices($\Delta\varepsilon_A$, -$\sum\alpha_X/\sum\alpha$, Δβ', -Δβ'$_S$, ∑α, -$\Delta\varepsilon_A$) + AlogP98<br><br>Non-ETA (CHI-V-0, SdO, (3.68239-SsCH3),(0.97222-SdsCH) +AlogP98<br><br>ETA+Non-ETA+AlogP98	Linear: R^2=0.764-0.790, R^2_{pred}=0.746-0.787<br><br>Spline: R^2=0.763-0.774, R^2_{pred}=0.777-783	Roy&Das(2012)[165]
	NL(MLPN, PNN, GRNN RBFN,SVM,GEP, DT)	−log LC50 fathead minnow	PNN: PD physico-chemical descriptor, CD constitutional descriptors,TD topological descriptor<br>GRNN: PD,CD,GD geometrical descriptor,TD	Best PNN and GRNN PNN: Train-95.85,Val-91.30,Complete-94.94 GRNN: Train-0.929,Val-0.910,Complete – 0.926	Singh et al.(2013)[166]
	NL(GA+kNN model),	-logLC50 D.magna	MLOgP, RDCHI, SAacc, TPSA (tot), H-050, nN, C-040,GATS1p	With average distance limit 1.26: n=436+110,	Cassoti et al.(2014)[12]

			Fingerprints-based	R^2= 0.78, Q^2cv=0.78<br>Q^2ext=0.72<br><br>R^2= 0.67, Q^2cv=0.67<br>Q^2ext=0.59<br><br>Consensus: R^2= 0.78,<br>Q^2cv=0.78 Q^2ext=0.73	
	L	pLC50 Pimephales promelas	VP-1, MFLER_BH, nAtomLAC,<br>- HybRatio, naasC, -nN	n=449, R^2=0.75, CCC_{ext}=0.84	Gramatica et al.(2014)[37]
	NL (ensemble models, classification decision treeboost, regression decision tree forest)	EC50/LC50, multispecies: Algae P. subcapitata (model building) (n=505)<br>Test species: algae S. obliguue , daphnia(n=547), fish(n=505), and bacteria.	Classification: VP-2, MDEC-22, BCUTp-1l, WL-2U, WL-3U, PPSA-1, PNSA-1, XLogP, nAtomP<br><br>Regression: SP-1, MDEC-23, MDEC-33, ATSp5, ATSm5, TopoPSA, XLogP, nHBDon	Classification: Model building (algae) species DTB:97.82%, DTF: 99.01%<br>Test species: 92.50%−94.26% and 92.14%−94.12% in four test species<br>Regression: DTB: 0.918, DTF: 0.905 (algae)<br>0.575 - 0.672, and 0.605−0.689 test species	Singh et al.(2014)[167]
Non-congeneric industrial chemicals	NL (DTB, DTF)	−log IGC50 T.pyriformis (n=1450)	Classification: CPSA.22, CIOO, ECI, Log P, MW, NALC, NALPS<br><br>Regression: CIOO, logP, MSA, MW, NALPS	Accuracies optimal models: optimal DTB 98.90%, DTF 98.83% (two-category) and 98.14%, 98.14% (four-category)<br><br>DTB: R^2=0.945, R^2_{test}=0.637(bacteria), R^2_{test}=0.741(algae)<br>DTF: R^2=0.944 R^2_{test}=0.655(bacteria), R^2_{test}=0.691(algae)	Singh &Gupta (2014)[168]
	L	pT algae C.vulgaris pT=1/log (EC50)	MLR model logP, H_Dz(Z)<br>Kring model (complex): VR1_B(s), R4i	n=73, r^2_{adj} = 0.922, r^2_{test} = 0.809	Tugcu et al.(2014)[169]

Various narcotic pollutants	L	logLC50, Poecilia reticulata	norm indices: -exp(1/MW), exp(-1/N), Emin, norm(MD, 2), norm(MD, fro). Parameters in Table 1.	n=190 R^2_{tr} = 0.9376, R^2_{test}=0.9264	Wang et al.(2014)[170]
	NL (GA+kNN k=6)	LC50 fathead minnow	MLOGP, CIC0, NdssC, NdsCH, SM1_Dz(Z), GATS1i	n=726+182, Q^2_{cv} = 0.61-0.89, Q^2_{ext} = 0.61-0.77	Cassotti et al.(2015)[171]
	L	D.magna	quantum chemical descriptors	n=252 (n_{tr}=113, n_{ts} =111) R^2=0.600-0.677	Vikas (2015)[172]
	L (MOA+MLR, single MLR), NL (hierarchical clustering HC)	LC50 fathead minnow	2D descriptors selected include autocorrelation, molecular fragment and E-state descriptors.10 out of 14 models contained some form of log K_{ow} descriptor	r^2 = 0.529–0.632 single MLR: r^2 = 0.551–0.562 HC: r^2 = 0.572, coverage = 99.3%	Martin et al.(2015)[173]
	L (Monte Carlo method)	pLC50 D.magna	Presence or absence of double (=), triple (#), and stereochemical (@) bonds; presence or absence of the chemical elements nitrogen (N), oxygen (O), sulfur (S), and phosphorus (P); presence or absence of the chemical elements fluorine (F), chlorine (Cl), bromine (Br), and iodine (I) (i.e.,halogens); and PAIR represents the simultaneous presence of pairs of the above-mentioned (in parentheses) SMILES elements.	n_{tr}=758, n_{valid} =87, r^2 = 0.8377	Toropova et al.(2016)[22]
	L	-logLC50 fathead minnow	Global model; log Kow, SM6B(p), − GATS1p , − SpMADEA, − HOMA, − SddsN, − NssCH2, B10[C − N]	n=963, (ntr=771, nvalid=192), GA-MLR,r^2_{adj}=0.701	Wu et al.(2016)[122]
	L, NL	pLC50 D.magna	AlogP, AATSC0p, Crippen logP, -minsOH, MLFERBH,XlogP	ACO+SVM r^2_{tr} =0.92, r^2_{tst}=0.83	Aalizadeh et al.(2017)[174]

				ACO+MLR r^2_{fit}=0.607, r^2_{tst}=0.733	
	NL (kNN)		Morgan, PaDEL, SiRMS, and DRAGON descriptors	n_{model}=644, n_{ext}=339, CCR: 86−88%.	Alves et al.(2018)[175]
	NL(6)	EC50, D. magna	CDK fngerprint (CDK, 1024 bits), Extended fngerprint (Ext, 1024 bits), Estate fngerprint (Est, 79 bits),MACCS fngerprint (Mac, 166 bits), PubChem fngerprint (Pub,881 bits), Substructure fngerprint (Sub, 307 bits), and Graph-Only fngerprint (Gra, 1024 bits).	Local (only D. magna, n=709) and global models (all kinds of crustacean data, n=115), different methods,EC50, Mac-SVM outperformed others in both local and global models (MACCS fingerprint -SVM) Train/test =80/20% data	Cao et al.(2018)[176]
	L	QSARINS PBT index	nX, nBondsM, -nHBDonLipinski, - MAXDP2	n=180, R^2=0.89	Gramatica et al.(2018)[102]
	L	pLC50 fathead minnow	norm descriptors	n=685, R^2 =0.8174, Q^2 = 0.7923	Jia et al (2018)[177]
		pLC50 D.magna,	calculations by PROGROC program	n_{tr}=376, n_{test}=170, R^2=0.971, R^2_{test}=0.952	Vazhev et al(2018)[178]
	L (5 models, consensus modeling)	pEC50 P. subcapitata	CrippenMR, LogKow, MLOGP, B06[C-N], B05[C-Cl], F02[N-S], H-051 and nSO2OH	n_{train} = 251, n_{test} = 83, R^2=0.71-0.72, Q^2=0.70	Khan&Roy (2019)[19]
	NL (classification ensemble models (RF to select most relevant features, SVM, extreme gradient boosting (XGBoost)) (6)	LC50, fathead minnow	Padel 2D molecular descriptors and molecular fingerprints	Best ensemble-SVM: Q values for the training set, validation set, and complete dataset were 92.2%, 87.3%, and 96.0%. AUC internal (92.2 and 0.965) AUC external validation (87.3 and 0.940) needs to be improved, overfitted model	Ai et al.(2019)[179]
Diverse organic chemicals including	NL (ML methods (RF, naïve Bayes, kNN, C4.5	mysid shrimp (local)	mindssC, CrippenLogP, maxHBint2, maxwHBa, GATS1i, hmin, SwHBa,	local, n_{tr}=309, n_{test}=77, Q_{tr}=0.7603-0.8171,	Liu et al.(2019)[180]

pesticides and industrial chemicals	decision tree, SVM, ANN)) (12)	marine crustaceans	nT6HeteroRing, MDEO-11,GATS4c.<br><br>XLogP, SHBd, maxHBint2, maxwHBa, mindssC, ALogP, SwHBa, GATS1i, GATS4c, AATSC1s, and MDEC-23.	Q_{test}=0.779-0.9032, Q_{valid}= 0.700-0.851<br><br>global models, n_{tr}=326, n_{test}=82, Q_{tr}=0.7397-0.82, Q_{test}=0.756-0.927, Q_{valid}= 0.728-0.824,	
	NL	Tetrahymena pyriformis	Counts of fragments having 2–4 heavy atoms (sirms tool of spci Software)	RF (Q^2=0.76), SVM(Q^2=0.73), GBM gradient boosting machine (Q^2=0.77), Consensus (RF,SVM, GBM=0.75), n=1984	Matveieva et al.(2019)[181]
Miscellaneous chemicals	L	pLC50 mixture of fishes (Brachdanio rerio, Pimephales promelas, Cyprinus carpio, Oryzias latipes, Poecilia reticulata, Lepomis macrochirus, and Oncorhyncus mykiss)	-BLTF96, X5 sol, X1 A, D/Dtr03, - F02[N-N], O-060, -B01[C-O], -S_A(type)/C. 1_C.1_C. 3_H/2_3s, 3_4s/4, NssS, -H%	n_{tr}=208, n_{test}=69, R^2=0.650, R^2_{pred}=0.610	Khan et al.(2019)[35]
	L	pLC50 mixture of fishes (Brachdanio rerio, Pimephales promelas, Cyprinus carpio, Oryzias latipes, Poecilia reticulata, Lepomis macrochirus, and	ALOGP, XMOD, Mv, -S_A(type)/C. 3_C. 3_H_O. 3/1_2s, 2_4s, 3_4s/6, B10[C-N], Fr3(rf)/A_B_B/1_2s, 2_3d/, -Fr3(type)/C. 3_C. 3_O. 3/1_3s, 2_3s/, F01[C-X], -Fr3(rep)/B_D_E/1_3s, 2_3d/ , -Fr3(att)/D_D_E/1_3s, 2_3s/,	n_{tr}=841, n_{test}=280, R^2=0.610, R^2_{pred}=0.630	Khan et al.(2019)[35]

		Oncorhyncus mykiss)			
	NL (RF, GBT, SVR)	LC50, NOEC different species of fish	PaDEL descriptors	LC50: R^2=0.59-0.64, R^2ev=0.57-0.66 NOEC: R^2=0.60-0.62, R^2ev=0.59	Sheffield &Judson(2019)[182]
	L (Monte carlo improved by index of ideality of correlation)	pLC50 Zebrafish (Danio rerio) Embryo	Dragon 7 descriptors	n=411, R^2=0.77(best)	Toropov et al.(2019)[183]
	NL (ANN)	log(1/LC50) fathead minnow	mined structural alerts	n=568 VEGA qsar, average accuracy training set >88%, average accuracy on the trout test set 69%	Gini et al.(2019)[10]
	L	pLC50 zebrafish embryo different exposure times (48(n=194), 96(n=68), 120(n=149) and 132h(n=143))	norm descriptors	R^2 = 0.8549, 0.9162, 0.8335 and 0.8119	Liu et al.(2020)[184]
	NL (ensemble modeling SVM)	pLC50/ pEC50 Algae(n=1440), daphnia(n=2120), fish(n=2110),	ISIDA Property-Label Molecular descriptors	CV r^2 values of 0.60, 0.72, 0.71.	Lunghini et al(2020)[185]
	NL (Random Forest (RF) and Gradient Boosting Machine (GBM), consensus models)	log(LC50) fathead minnow and Daphnia magna log(IGC50) Tetrahymena pyriformis	Structural fragments (sirms tool of spci Software)	Fish (n_{tr}=642, n_{test}=161): R^2_{cv}=0.65, R^2_{test}=0.59. $R^2_{AD\text{-}only}$=0.49 (GBM), R^2_{cv}=0.66, R^2_{test}=0.56. $R^2_{AD\text{-}only}$=0.56 (RF), R^2_{cv}=0.68, R^2_{test}=0.60. $R^2_{AD\text{-}only}$=0.54 (Consensus model)<br><br>Daphnia (n_{tr}=268, n_{test}=67):R^2_{cv}=0.52, R^2test=0.70. $R^2_{AD\text{-}only}$=0.52 (GBM),	Tinkov et al(2020)[186]

				R^2_{cv}=0.50, R^2_{test}=0.70. $R^2_{AD-only}$=0.53 (RF), R^2_{cv}=0.53, $R2_{test}$=0.71. $R^2_{AD-only}$=0.53 (Consensus model),<br><br>Algae (n_{tr}=1424, n_{test}=356): R^2_{cv}=0.77, R^2_{test}=0.77. $R^2_{AD-only}$=0.76 (GBM), R^2_{cv}=0.75, R^2_{test}=0.76. $R^2_{AD-only}$=0.73 (RF), R^2_{cv}=0.78, R^2_{test}=0.78. $R^2_{AD-only}$=0.79 (Consensus model)	
	NL (10 radial basis function neural network and its consensus modeling)	-log LC50, fathead minnow	PaDEl descriptors XlogP, Crippen log P, AMR, AATS4v, GATS1i, GATS1v, khs.dsch, MATS1c, AATS4v, GATS6i, GATS1m, MlogP, and nN	n=955, R^2_{cv10}>0.7, R^2_{adj}>0.8, Q^2_{ext}=0.6480-0.7317, R^2_{ext}=0.6563-0.7318 Consensus model: R^2 =0.9118, R^2_{cv10} =0.7632, and Q^2_{ext}=0.7430.	Wang&Chen(2020)[187]
	NL(SVM+GA)	pEC10 Pseudokirchneriella subcapitata	CrippenMR, MHYD, F02[N-S], B05[C-Cl], CCCN and R3m	n_{tr}=167, R^2_{tr}=0.76, n_{test}=167R^2_{test}=0.75	Yu(2020)[188]
	NL (general regression neural network (GRNN))	pIGC50 Tetrahymena pyriformis	ALOGP2, GATS1p, MLIP and MW MLIP = nRNCS + nDB – nROH,	n=1163, R^2=0.85	Yu(2020)[189]

Table S7. Interspecies QSAAR models

Applicability Domain	Models	Endpoint, species	Descriptors	Performance	Reference
Aliphatic compounds	L	log(1/IGC50) T. pyriformis	pT15 V. fischeri	n = 64, r^2 = 0.850	Cronin et al. (2000)[26]
C=O: Aldehydes	L	log(1/IGC50) T. pyriformis or	log(1/IGC50) T. pyriformis or LC50 fathead minnow,	n=143, r^2=0.698	Dimitrov et al.(2004)[190]

		LC50 fathead minnow	logKow, $D_{O\text{-}atom}$ (reactivity)		
Global: Diverse chemicals	L	log(1/LC50), P. promelas	log(1/IGC50), T.pyriformis, P_C^{avg} , -#Nrel, -HACA2	n = 362, R^2 = 0.851, R^2_{CV} = 0.846	Kahn et al.(2007)[191]
Different chemicals	L	log1/IC50 Bacteria<br>log1/IC50 Algae<br>log1/IC50 Algae<br>log1/IGC50 T. pyriformis<br>log1/IGC50 T. pyriformis<br>log1/IC50 D. magna<br>log1/IC50 Fathead<br>log1/IC50 Guppy	log1/EC50 V. fischeri<br>log1/EC50 V. fischeri, -ELUMO<br>log1/IC50 D.magna, -ELUMO<br>log1/EC50 V. fischeri, -ELUMO<br>log1/EC50 D.magna, -ELUMO<br>log1/EC50 V. fischeri, log P<br>log1/EC50 V. fischeri, log P<br>log1/EC50 V. fischeri, log P	n=23, R^2=0.84<br>n=30, R^2=0.84<br>n=37, R^2=0.83<br>n=49, R^2=0.62<br>n=56, R^2=0.73<br>n=71, R^2=0.80<br>n=56, R^2=0.89<br>n=56, R^2=0.87	Zhang et al.(2010)[192]
Different chemicals	L	log (1/EC50) algae	log (1/EC50) daphnid toxicity	n=103, r^2_{adj}=0.81, q^2= 0.80	Furuhama et al.(2016)[193]
Different chemicals	L	lg(LD50 rats/LC^e50 rainbow trout)	lgP.	R^2=0.962	Zolotarev et al.(2016)[194]
Overall compounds	L	log 1/EC50 D.magna<br>log1/IGC50 T. pyriformis<br>log1/IBC50 V. fischeri<br>log1/IGC50 T. pyriformis<br>log1/IBC50 V. fischeri<br>log1/IBC50 V. fischeri	log 1/LC50 Fish (mix of species)<br>log 1/LC50 Fish (mix of species)<br>log 1/LC50 Fish (mix of species)<br>log 1/EC50 D.magna<br>log 1/EC50 D.magna<br>log1/IBC50 V. fischeri	n=467, R^2=0.72<br>n=478, R^2=0.72<br>n=304, R^2=0.54<br>n=287, R^2=0.63<br>n=294, R^2=0.45<br>n=556, R^2=0.63	Li et al.(2018)[195]
Different chemicals	L	pIGC50 T. pyriformis<br><br>pIC50-D.magna<br><br>pLC50-fish (mix of species)	pIC50-DM, SM1_B(m), MLOGP<br><br>pLC50-fish, X3v, MATS1s<br><br>pIGC50-TP, SM14_AEA(ri), SAdon	n=310, r^2=0.80, Q^2=0.79, Q^2_{ext}=0.73<br>n=608, r^2=0.70, Q^2=0.69, Q^2_{ext}=0.70<br>n=518, r^2=0.77, Q^2=0.76, Q^2_{ext}=0.61	Bouhedjar et al.(2020)[196]

		pIBC50 V. fischeri	pIC50-DM, MATS1s, ALOGP<br><br>pIBC50 -VF, IDET, SpPosA_B(p)<br><br>pIGC50-TP, Mp, SpMax4_Bh(p)	n=608, r^2=0.68, Q^2=0.67, Q^2_{ext}=0.74<br>n=355, r^2=0.66, Q^2=0.65, Q^2_{ext}=0.61<br>n=570, r^2=0.73, Q^2=0.73, Q^2_{ext}=0.75	
Aliphatic isothiocyanates	L	log(1/IGC50) T. pyriformis	Acute aquatic toxicity vs thiol reactivity 1.33(log(1/EC50)) -0.41	n=23, r^2 = 0.911, q^2= 0.907	Schultz et al.(2007)[197]
Halo-substituted carbonyl compounds (esters, phenones and amides),	L	log(1/IGC50) T. pyriformis	Acute aquatic toxicity - thiol reactivity: log (1/IGC50)=0.848(log(1/EC50)) +1.40	n=19, r^2=0.926, r^2(pred)=0.905,	Schultz et al.(2007)[198]
MOA: Nonpolar narcosis	L	log(1/LC50), guppy (G)<br><br>log(1/LC50), fathead minnow (FHM),	log 1/LC50(FHM)exp<br>log 1/LC50(RT)exp<br><br>log 1/LC50(RT)exp	n=39, R^2=0.988<br>n=27, R^2=0.939<br><br>n=19, R^2=0.957	Raevsky et al.(2008)[131]
Baseline compounds	L	log 1/EC50 D.magna<br>log1/IGC50 T. pyriformis<br>log1/IBC50 V. fischeri<br>log1/IGC50 T. pyriformis<br>log1/IBC50 V. fischeri<br>log1/IBC50 V. fischeri	log 1/LC50 Fish (mix of species)<br>log 1/LC50 Fish (mix of species)<br>log 1/LC50 Fish (mix of species)<br>log 1/EC50 D.magna<br>log 1/EC50 D.magna<br>log1/IBC50 V. fischeri	n=92, R^2=0.83<br>n=71, R^2=0.93<br>n=72, R^2=0.73<br>n=42, R^2=0.81<br>n=63, R^2=0.65<br>n=64, R^2=0.79	Li et al.(2018)[195]
Less inert compounds	L	log 1/EC50 D.magna<br>log1/IGC50 T. pyriformis<br>log1/IBC50 V. fischeri	log 1/LC50 Fish (mix of species)<br>log 1/LC50 Fish (mix of species)<br>log 1/LC50 Fish (mix of species)	n=52, R^2=0.87<br>n=102, R^2=0.69<br>n=75, R^2=0.50<br>n=45, R^2=0.47<br>n=37, R^2=0.58<br>n=112, R^2=0.42	Li et al.(2018)[195]

		log1/IGC50 T. pyriformis<br>log1/IBC50 V. fischeri<br>log1/IBC50 V. fischeri	log 1/EC50 D.magna<br>log 1/EC50 D.magna<br>log1/IBC50 V. fischeri		
Reactive compiunds	L	log 1/EC50 D.magna<br>log1/IGC50 T. pyriformis<br>log1/IBC50 V. fischeri<br>log1/IGC50 T. pyriformis<br>log1/IBC50 V. fischeri<br>log1/IBC50 V. fischeri	log 1/LC50 Fish (mix of species)<br>log 1/LC50 Fish (mix of species)<br>log 1/LC50 Fish (mix of species)<br>log 1/EC50 D.magna<br>log 1/EC50 D.magna<br>log1/IBC50 V. fischeri	n=67, R^2=0.66<br>n=79, R^2=0.59<br>n=26, R^2=0.54<br>n=49, R^2=0.73<br>n=42, R^2=0.40<br>n=69, R^2=0.78	Li et al.(2018)[195]
Organothiophosphate pesticides	L	logEC50, vertebrate carp	logEC50 D.magna	n=9, r^2= 0.94, r^2_{int}=0.90	Zvinavashe et al.(2009)[89]
Benzene derivatives	L	pLC50, P. promelas<br><br>pLC50, P. reticulata<br><br>pLC50, R. japonica	CrippenLogP, pIGC50 T. pyriformis<br><br>ALogP2, pIGC50 T. pyriformis<br><br>XLogP, pIGC50 T. pyriformis	n = 25, R^2=0.783<br><br>n = 46, R^2= 0.611<br><br>n = 21, R^2=0.836	Gupta et al.(2015)[58]
Aromatic amines and phenols	L	log(1/LC50) fish Oryzias latipes<br>log(1/EC50) P. subcapitata	log(1/EC50) daphnia, MW, substructures	n=109, r^2_{adj}=0.73<br><br>n=104, r^2=0.73	Furuhama et al.(2015)[199]
Substituted phenols	L	$pT_{C.vulgaris}$<br><br>$pT_{C.vulgaris}$<br>pTP.subcapitata=	$pT_{T.pyriformis}$ (−log IGC50)<br><br>$pT_{P.subcapitata}$ (−logEC50)	n_{tr}=31 R^2=0.75, n_{pr}=10, R^2=0.82<br><br>n_{tr}=16, R^2=0.93, n_{pr}=6, R^2=0.83	Tugcu et al.(2017)[78]

Pharmaceutical and Personal Care Products	L	pEC50 O.mykiss<br>pEC50 O.mykiss<br>pEC50 P.promelas<br>pEC50 P.promelas	pEC50 D.magna, - GATS1e<br>pEC50 P.promelas, AATSC0v<br>pEC50 D.magna, ATS4s<br>pEC50 O.mykiss, AATS7p	n=50, r=0.88<br>n=34, r=0.95<br>n=42, r=0.86<br>n=34, r=0.95	Sangion&Gramatica (2016)[101]
Contaminants of Emerging Concern (pharmaceuticals and personal care products (PPCPs), UV filters, hormones and endocrine disrupting chemicals (EDCs), pesticides and surfactants etc.)	L	pLC50japonica<br><br>pLC50P.promelas:	pLC50D.magna, B08[C-O], -B09[N-O]<br><br>C-006, -H-052, -pLC50japonica	n_{tr}=36, n_{test}=11, R^2adj=0.66, R^2pred=0.88<br><br>n_{tr}=15,n_{test}=4, R^2_{adj}=0.76, R^2_{pred}=0.84	Hossain&Roy(2018)[111]
CECs	L	pLC50, D. japonica	pEC50 D.magna	n_{tr}=19, n_{test}=7, R^2_{adj} = 0.706, R^2_{pred} = 0.839	Önlü &Saçan (2018)[112]
Pharmaceuticals	L	fish Brachydanio rerio<br><br>Algae Scenedesmus subspicatus<br><br>Daphnia magna<br><br>Algae<br><br>Fish<br><br>Daphnia	-CATS3D_05DP, algae toxicity<br><br>C-019, fish toxicity<br><br>C%, algae toxicity<br><br>SHBint3, daphnia toxicity<br><br>CATS_3D_15DL, daphnia toxicity<br><br>-ATSC7c, Fish toxicity	n_{tr}=70,n_{test}=20, R^2=0.74, R^2_{pred}=0.76,<br>n_{tr}=67,n_{test}=23, R^2=0.725, R^2_{pred}=0.81<br>n_{tr}=76,n_{test}=20, R^2=0.701, R^2_{pred}=0.704<br>n_{tr}=73,n_{test}=23, R^2=0.66, R^2_{pred}=0.81<br>n_{tr}=73,n_{test}=22, R^2=0.76, R^2_{pred}=0.79<br>n_{tr}=71,n_{test}=24, R^2=0.72, R^2_{pred}=0.78	Khan et al.(2019)[21]
Industrial chemicals	L	log LC50 AFT(acute fish toxicity)	log LC50 ZFET(Zebrafish embryo toxicity)	n=258 R^2 = 0.63	Zhu et al.(2018)[137]

S2. Kowmine package and keyphrase extraction

The automated text mining procedure is presented in Figure S4. To perform the procedure, a python-based *knowmine* package was developed. The main idea behind the package is to extract sentences that have the defined main toxicity terms and connection words in key phrases of the sentence. The package consists of several modules: *FilesReader, TextExtractor, AllSentencesExtractor, KeywordsExtractor, RelevantSentencesExtractor* and *Output-fileGenerator*.

The FilesReader module provides a function accessing the files in a User-provided folder and returning the list of file names. The TextExtractor module allows to extract and clean text from the pdf articles. The AllSentencesExtractor module provides functionality to extract sentences from the texts of the articles. The RelevantSentencesExtractor module identifies the sentences containing the provided main terms and connection words as keywords of the sentence. The KeywordsExtractor module performs the keywords extraction. To reduce the computational time of the process, only the sentences containing the main terms are considered for the keyphrase extraction. The KeywordsExtractor utilizes a pke module applying an extraction model. The extraction model used in the current study was a graph-based Single Rank model[6]. However, several unsupervised (statistical and graph-based) and supervised models are available (feature-based models)[5]. The statistical methods are based on term frequency calculation. The graph-based models build a word graph where nodes correspond to words and edges correlate to word association patterns. The highest value nodes are ranked by graph centrality measures and are considered the keyphrases[200]. The benefit of the graph-based models for the current study is the possibility to specify part-of-speech tags (word labels corresponding to a part of speech: noun,

verb, adjective, etc.) for the candidate words. The supervised models have been trained on the SemEval-2010 dataset[201].

The choice of the Single Rank model for the current study was motived by the results of a simple sensitivity analysis (Tables S8 and S9). The analysis was performed on six different models, including the latest statistical model (YAKE[202]), four graph-based models (TopicRank[203], SingleRank[6], PositionRank[200], and MultipartiteRank[204]), and one supervised model (WINGNUS[205]). The default model parameters were used except for the graph-based model, where pronouns were removed, and verbs were included as valid parts of speech. No grammar was defined for the PositionRank model.

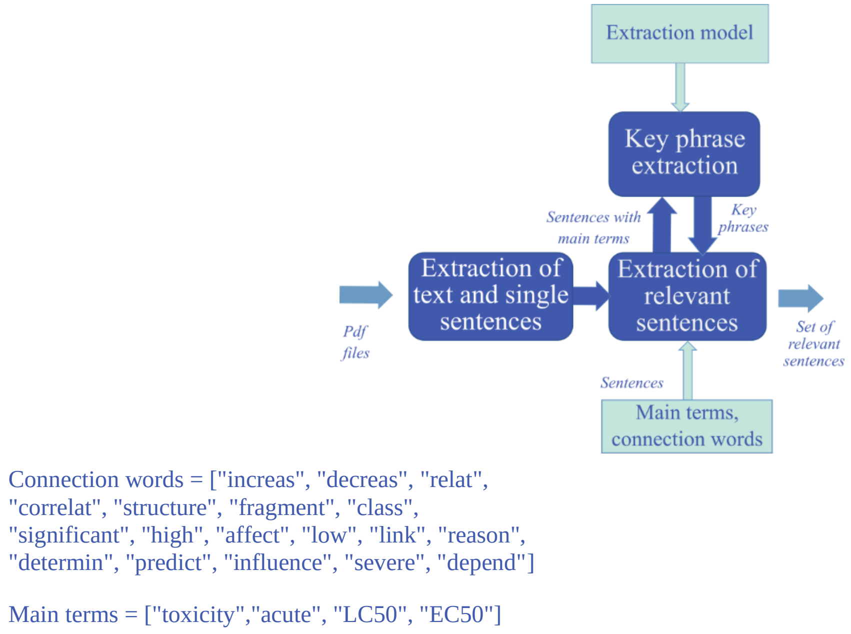

Figure S4. The automated text mining procedure

First, models were applied to extract key phrases from short texts (containing 1-2 sentences). The evaluation of the results (Table S8) showed that the extracted key phrases differ more when the text is longer (2 sentences). It can also be seen that in the case of YAKE, some of the words

are repeated. The key phrases extracted by WINGNUS contain only nouns and adjectives. Since the WINGNUS model is based on the pre-trained model, altering the parameters is not easy. Thus, it was decided to proceed with the graph-based models. The four models were applied to several articles to select one of the graph-based models. The similar number of sentences were extracted by the applied models (Table S9). The manual evaluation of the extracted sentences showed a slightly better performance of the Single Rank model.

OutputfileGenerator helps generate the output file of the desired format (*SQLite* database or excel) containing the path to the file, extracted sentences, number of sentences in the original text (after the cleaning), and number of the extracted sentences. The result "Extracted sentences" file is generated in the User-provided folder containing the files for mining. In case there are no extracted sentences, the article file might be unreadable by the implemented pdf mining packages and require a different solution like image recognition or manual sentence extraction.

The knowmine package is available for installation via *pip*, the source files can be retrieved from https://github.com/GulnaraSh/Knowledge-mining-python

Table S8. Results of the different key phrase extraction models by the pke-package applied to short texts prior to the method presented in Figure S4 (i.e., unsupervised methods in terms of predefined keywords and main terms).

Texts	Yake	TopicRank	SingleRank	PositionRank	MultipartiteRank	WINGNUS
Their concentrations in the environment, particularly highest in towns and urban regions, are highest, where the continuous combustion of fossil fuels and industrial activities take place in dense and confined environments[206].	[‘industrial activities take, activities take place, urban regions, confined environments, particularly highest, continuous combustion, fossil fuels, activities take, take place, highest’]	[‘highest, environment, industrial activities take place, towns, fossil fuels, urban regions, continuous combustion, dense, concentrations’]	[‘industrial activities take place, continuous combustion, fossil fuels, urban regions, confined environments, highest, dense, towns, environment, concentrations’]	[‘urban regions, continuous combustion, fossil fuels, industrial activities, concentrations, towns, environment, place, environments’]	[‘highest, environment, towns, urban regions, industrial activities take place, fossil fuels, continuous combustion, dense, concentrations, confined environments’]	['urban regions', 'continuous combustion', 'fossil fuels', 'industrial activities', 'towns', 'concentrations', 'environment', 'place']
However, the concentration of PAHs may become excessively high upon the occurrence of oil spills, industrial catastrophes, and regular dumping of industrial waste (Jung et al. 2011; Vidal et al. 2010) and lead to the formation of super-agglomerates and contaminated areas, which require decades of transformation and degradation to return to normal and viable condition[206].	[‘pahs may become, may become excessively, become excessively high, excessively high upon, pahs may, industrial catastrophes, industrial waste, oil spills, may become, excessively high’]	[‘industrial catastrophes, degradation, transformation, return, oil spills, require decades, normal, pahs may become, regular dumping, occurrence’]	[‘industrial catastrophes, industrial waste, pahs may become, oil spills, require decades, contaminated areas, regular dumping, -agglomerates, viable condition, transformation’]	[‘oil spills, industrial catastrophes, industrial waste, regular dumping, occurrence, concentration, pahs, formation, viable condition, transformation’]	[‘industrial catastrophes, oil spills, regular dumping, degradation, occurrence, transformation, return, pahs may become, high, require decades’]	[‘oil spills, industrial catastrophes, regular dumping, pahs, industrial waste, viable condition, concentration, occurrence, decades, formation’]

PAHs are also rigorously produced by natural processes such as volcanic activity and generic combustion of biomasses and take their place in the natural chains of chemotransformation, through absorption and biotransformation in microorganisms in aquatic and soil ecosystems. In soil samples for instance, several bacterial species have been found to be able to degrade small and medium-sized PAHs (2,3–5-6) (Zhang et al. 2006; Johnsen et al. 2005; Chaudhary et al. 2011; Song et al. 2011)[206].	[‘also rigorously produced, also rigorously, rigorously produced, volcanic activity, generic combustion, biotransformation in microorganisms, natural processes, natural chains, soil ecosystems, natural’]	[‘soil ecosystems, microorganisms, biotransformation, aquatic, take, absorption, pahs, biomasses, natural chains, generic combustion’]	[‘natural processes such, several bacterial species, soil samples, soil ecosystems, natural chains, generic combustion, volcanic activity, degrade small, sized pahs, able’]	[‘sized pahs, natural processes, pahs, natural chains, volcanic activity, generic combustion, several bacterial species, soil samples, soil ecosystems, biomasses’]	[‘pahs, soil ecosystems, aquatic, microorganisms, take, generic combustion, biomasses, biotransformation, natural chains, place’]	[‘pahs, volcanic activity, generic combustion, natural processes, natural chains, biomasses, chemotransformation, several bacterial species, soil ecosystems, absorption’]
During the aftermath of such disasters, the biotransformation and biodegradation processes that take place are mainly carried out by PAH-degrading fungi and bacteriand chemical processes, which have limitations in context with the damage caused[206].	[‘bacteriand chemical processes, degrading fungi, damage caused, take place, mainly carried, bacteriand chemical, biodegradation processes, chemical processes, pah, processes’]	[‘biodegradation processes, take place, bacteriand chemical processes, context, limitations, degrading fungi, biotransformation, carried, damage caused, aftermath’]	[‘bacteriand chemical processes, biodegradation processes, take place, degrading fungi, such disasters, damage caused, carried, limitations, biotransformation, context’]	[‘bacteriand chemical processes, biodegradation processes, such disasters, biotransformation, aftermath, place, limitations, fungi, context, damage’]	[‘biodegradation processes, take place, bacteriand chemical processes, context, limitations, degrading fungi, biotransformation, carried, damage caused, aftermath’]	[‘such disasters, aftermath, biodegradation processes, biotransformation, bacteriand chemical processes, pah, fungi, damage, place, context’]

For instance, biodegradation of PAHs require specific temperatures, pressures, and chemical (nutritive) environments to give fast-progressing transformation of the hazardous organic matter to carboxylic acids and CO_2 (Pandey et al. 2012)[206].	[‘pahs require specific, require specific temperatures, hazardous organic matter, pahs require, specific temperatures, give fast, progressing transformation, require specific, hazardous organic, organic matter’]	[‘pahs require specific temperatures, environments, chemical, give, pressures, hazardous organic matter, carboxylic acids, biodegradation, progressing transformation, instance’]	[‘pahs require specific temperatures, hazardous organic matter, progressing transformation, carboxylic acids, pressures, chemical, give, environments, biodegradation, instance’]	[‘specific temperatures, hazardous organic matter, instance, pressures, pahs, biodegradation, environments, carboxylic acids, transformation, co2’]	[‘pahs require specific temperatures, environments, chemical, give, pressures, hazardous organic matter, carboxylic acids, biodegradation, progressing transformation, instance’]	[‘specific temperatures, biodegradation, pahs, hazardous organic matter, carboxylic acids, pressures, co2, instance, transformation, environments’]
Degradation of small and medium-sized PAHs has also been successfully carried out by the fungi Phanerochaete and the bacterial Serratistrains (Johnsen et al. 2005; Horel et al. 2012; Yan et al. 2004; Pandey et al. 2012); however, biodegradation of PAHs larger than coronene (more than 7 benzene rings) is difficult if not impossible to degrade by biological processes solely and requires chemical decomposition either by addition of chemicals or oxidation over time from atmospheric or sub-aquatic oxygen[206].	[‘bacterial serratistrains, fungi phanerochaete, sized pahs, successfully carried, serratistrains, phanerochaete, pahs larger, pahs, aquatic oxygen, biological processes solely’]	[‘requires chemical decomposition, degradation, sized pahs, oxidation, biological processes, impossible, time, addition, biodegradation, difficult’]	[‘sub - aquatic oxygen, requires chemical decomposition, sized pahs, pahs larger, biological processes, oxidation, time, chemicals, addition, impossible’]	[‘sized pahs, pahs, degradation, bacterial serratistrains, biological processes, fungi phanerochaete, biodegradation, chemical decomposition, oxidation, addition’]	[‘degradation, sized pahs, requires chemical decomposition, medium, biological processes, small, addition, oxidation, impossible, time’]	[‘sized pahs, fungi phanerochaete, bacterial serratistrains, biodegradation, pahs, coronene, chemical decomposition, - aquatic oxygen, biological processes, chemicals’]

In this context, geological processes represent eventually the full remediator of disasters of such large and persistent scale, whereas examples such as Oil-sand drilling in Canada show no or low remediation of heavy PAHs in the soil and groundwater reserves (Wayland et al. 2008; Deepthike et al. 2009)[206].	[‘geological processes represent, processes represent eventually, heavy pahs, geological processes, persistent scale, whereas examples, sand drilling, groundwater reserves, processes represent, represent eventually’]	[‘full remediator, heavy pahs, geological processes represent, sand drilling, soil, disasters, oil, groundwater reserves, context, persistent scale’]	[‘such large, examples such, geological processes represent, persistent scale, full remediator, low remediation, sand drilling, heavy pahs, groundwater reserves, disasters’]	[‘full remediator, geological processes, persistent scale, sand drilling, context, disasters, low remediation, heavy pahs, examples, oil’]	[‘full remediator, disasters, geological processes represent, heavy pahs, sand drilling, context, oil, persistent scale, soil, low remediation’]	
A complete removal of the most resistant organic species is often not feasible without damaging the environment to such an extent that the local biotis affected from the DNA level for generations to come[206].	[‘resistant organic species, feasible without damaging, local biotis affected, dna level, complete removal, resistant organic, organic species, feasible without, without damaging, local biotis’]	[‘damaging, environment, generations, dna level, feasible, local biotis affected, extent, come, resistant organic species, complete removal’]	[‘local biotis affected, resistant organic species, dna level, complete removal, environment, extent, damaging, feasible, generations, come’]	[‘resistant organic species, complete removal, local biotis, dna level, environment, extent, generations’]	[‘damaging, environment, generations, dna level, feasible, local biotis affected, extent, come, resistant organic species, complete removal’]	[‘resistant organic species, complete removal, local biotis, dna level, extent, environment, generations’]
Given that the direct interaction with DNA is specifically related to the type of PAH (nitro, oxy, bay-region, acene, etc.), the complete profiling of types of	[‘direct interaction, specifically related, also electronic properties, pah,	[‘type, chemical properties, pahs, assessed, measured, important, complete	[‘such degradation processes, chemical properties, complete	[‘direct interaction, complete profiling, such degradation processes, type,	[‘type, chemical properties, measured, pahs, assessed, important, structure,	[‘pah, direct interaction, dna, complete profiling, such degradation processes, metabolites,

PAHs and metabolites from such degradation processes and from the nondegraded fractions are important to be assessed and measured against their chemical properties, structure, and also electronic properties[206].	nitro, oxy, bay, region, acene, etc’]	profiling, metabolites, nondegraded fractions, structure’]	profiling, nondegraded fractions, electronic properties, direct interaction, metabolites, important, types, related’]	types, metabolites, chemical properties, pahs, electronic properties, fractions’]	complete profiling, nondegraded fractions, metabolites’]	chemical properties, electronic properties, type, fractions’]
This review adds information to this quest, by introducing crucial electronic aspects of aromatic compounds frequently found in regions and areas exposed to such disasters[206].	[‘review adds information, introducing crucial electronic, crucial electronic aspects, aromatic compounds frequently, compounds frequently found, review adds, adds information, introducing crucial, crucial electronic, electronic aspects’]	[‘aromatic compounds, found, regions, introducing crucial electronic aspects, areas exposed, quest, review adds information’]	[‘introducing crucial electronic aspects, aromatic compounds, review adds information, areas exposed, such disasters, found, regions, quest’]	[‘crucial electronic aspects, aromatic compounds, quest, review, information, such disasters, regions, areas’]	[‘aromatic compounds, found, regions, introducing crucial electronic aspects, areas exposed, quest, review adds information’]	[‘crucial electronic aspects, quest, aromatic compounds, such disasters, review, information, regions, areas’]
The results of the deposition of PAHs in the environment in such extraordinary cases have been found to give devastating damages to fish, bird, and wild life in the affected regions for decades to last (Neff et al. 2011)[206].	[‘give devastating damages, extraordinary cases, give devastating, devastating damages, wild life, affected	[‘give devastating damages, fish, bird, affected regions, decades, wild life, pahs, deposition, found, last’]	[‘give devastating damages, such extraordinary cases, wild life, affected regions, bird, fish, found, environment, pahs, deposition’]	[‘such extraordinary cases, devastating damages, environment, wild life, deposition, results, pahs, affected regions, bird, decades’]	[‘give devastating damages, fish, bird, affected regions, decades, wild life, pahs, deposition, found, last’]	[‘such extraordinary cases, deposition, pahs, devastating damages, wild life, affected regions, bird, results, environment, decades’]

	regions, neff, bird, pahs, fish’]					
Short to long chain alcohols have a range of ecotoxicity to aquatic life driven by hydrophobic interactions with biological membranes[207].	[’long chain alcohols, aquatic life driven, biological membranes, long chain, chain alcohols, aquatic life, life driven, hydrophobic interactions, short, membranes’]	[‘aquatic life driven, ecotoxicity, hydrophobic interactions, range, long chain alcohols, biological membranes, short’]	[‘aquatic life driven, long chain alcohols, hydrophobic interactions, biological membranes, ecotoxicity, range, short’]	[’long chain alcohols, aquatic life, hydrophobic interactions, ecotoxicity, range, biological membranes’]	[‘aquatic life driven, ecotoxicity, hydrophobic interactions, range, long chain alcohols, biological membranes, short’]	[’long chain alcohols, aquatic life, ecotoxicity, hydrophobic interactions, biological membranes, range’]
Furthermore, there is a linear relationship between both acute and chronic toxicities and LogKow, suggesting that with the increase of hydrophobicity the aquatic toxicity increases[208].	[‘aquatic toxicity increases, toxicity increases, linear relationship, acute and chronic, chronic toxicities, aquatic toxicity, furthermore, logkow, toxicities and logkow, suggesting’]	[‘increase, hydrophobicity, chronic toxicities, acute, suggesting, linear relationship’]	[‘aquatic toxicity increases, chronic toxicities, linear relationship, increase, suggesting, acute, hydrophobicity’]	[‘chronic toxicities, linear relationship, aquatic toxicity increases, increase, hydrophobicity, logkow’]	[‘increase, hydrophobicity, chronic toxicities, acute, suggesting, linear relationship, aquatic toxicity increases’]	[‘linear relationship, chronic toxicities, logkow, aquatic toxicity increases, hydrophobicity, increase’]

Aldehydes, alcohols and acids, as well as the parabens used as preservatives, are readily biodegradable and present moderate toxicity to aquatic life[209].	[‘present moderate toxicity, aquatic life, parabens used, readily biodegradable, present moderate, moderate toxicity, aldehydes, alcohols, acids, preservatives’]	[‘present moderate toxicity, alcohols, preservatives, biodegradable, parabens used, acids, aldehydes, aquatic life’]	[‘present moderate toxicity, parabens used, aquatic life, biodegradable, preservatives, acids, alcohols, aldehydes’]	[‘present moderate toxicity, acids, alcohols, aldehydes, parabens, preservatives, aquatic life’]	[‘present moderate toxicity, alcohols, preservatives, biodegradable, parabens used, acids, aldehydes, aquatic life’]	[‘aldehydes, acids, alcohols, parabens, present moderate toxicity, aquatic life, preservatives’]
This paper reveals that the fish embryo toxicity can be used to predict whole fish toxicity for most of compounds[137].	[‘predict whole fish, fish embryo toxicity, whole fish toxicity, paper reveals, predict whole, fish embryo, embryo toxicity, whole fish, fish toxicity, compounds’]	[‘predict whole fish toxicity, used, compounds, paper reveals’]	[‘fish embryo toxicity can, predict whole fish toxicity, paper reveals, used, most, compounds’]	[‘fish embryo toxicity, whole fish toxicity, paper, compounds’]	[‘predict whole fish toxicity, used, compounds, paper reveals’]	[‘fish embryo toxicity, whole fish toxicity, paper, compounds’]
The aim of this study was to build a QSAR model-based set of theoretical molecular descriptors using acute fish toxicity values for compounds defined as MoA 1 to identify the molecular properties related to this mechanism and predict the fish toxicity of untested compounds[139].	[‘untested com- pounds, oretical molecular descriptors, descriptors using acute, using acute fish, acute fish toxicity, fish toxicity values, molecular descriptors using, molecular properties lated,	[‘compounds defined, predict, fish toxicity, molecular properties related, mechanism, model, build, identify, based set, study’]	[‘theoretical molecular descriptors using acute fish toxicity values, molecular properties related, fish toxicity, compounds defined, based set, untested compounds, model, predict,	[‘theoretical molecular descriptors, fish toxicity, molecular properties, untested compounds, qsar model, aim, set, study, compounds, mechanism’]	[‘compounds defined, molecular properties related, fish toxicity, predict, model, mechanism, identify, build, based set, study’]	[‘theoretical molecular descriptors, qsar model, acute fish toxicity, molecular properties, moa, fish toxicity, untested compounds, compounds, aim, study’]

	qsar model, fish toxicity’]		mechanism, identify’]			
Furthermore, ionized compounds may exhibit stronger interactions with biological membranes than other narcotics, or cause toxicity by an entirely different mode of action than their neutral counterparts[210].	[‘ionized compounds may, compounds may exhibit, may exhibit stronger, exhibit stronger interactions, entirely different mode, neutral counterparts, compounds may, may exhibit, exhibit stronger, stronger interactions’]	[‘action, different mode, neutral counterparts, cause toxicity, biological membranes’]	[‘ionized compounds may exhibit stronger interactions, other narcotics, biological membranes, cause toxicity, different mode, neutral counterparts, action’]	[‘biological membranes, ionized compounds, stronger interactions, other narcotics, different mode, toxicity, neutral counterparts, action’]	[‘action, different mode, neutral counterparts, cause toxicity, biological membranes’]	[‘ionized compounds, stronger interactions, biological membranes, other narcotics, neutral counterparts, different mode, toxicity, action’]
Among standalone, user-operated tools ECOSAR outperforms the other algorithms. When using any of these tools, users must be careful to consider the applicability domains and solubility warnings, which are not always available in batch mode[210].	[‘operated tools ecosar, tools ecosar outperforms, among standalone, ecosar outperforms, tools ecosar, operated tools, user, ecosar, among, standalone’]	[‘user, operated tools, consider, careful, applicability domains, solubility warnings, outperforms, available, using, standalone’]	[‘operated tools, solubility warnings, applicability domains, users must, other algorithms, tools, batch mode, careful, using, consider’]	[‘other algorithms, tools ecosar, tools, user, applicability domains, solubility warnings, users, batch mode’]	[‘user, operated tools, outperforms, standalone, careful, consider, applicability domains, users must, using, tools’]	[‘tools ecosar, solubility warnings, other algorithms, applicability domains, batch mode, user, tools’]

Table S9. Number of relevant sentences obtained from articles by different key phrase extraction models.

	TopicRank	SingleRank	PositionRank	MultipartiteRank
Abe et al. (2001)[211]	16	16	7	15
Ahlers et al. (2019)[212]	17	17	10	18
Cassotti et al. (2014)[213]	24	24	11	23
Dimitrov et al. (2003)[128]	15	17	12	16
Manzetti (2012)[206]	2	2	2	2

S3. Models

The nearest neighbors were identified in two ways: a Tanimoto similarity[214] between molecular fingerprints and a Manhattan distance[214] between the molecular descriptor vectors representing the molecules. The optimal number of neighbors was obtained via a cross-validation procedure (CV)[1]. The CV for the standard models was performed as follows: the dataset was divided into an external test and training data (according to an external set ratio). The training data were then further divided into the test and the training set (according to the CV ratio). The test set was used to find the number of neighbors giving to the highest value of Spearman rank-order correlation coefficient Spr (Spearman-based) or the coefficient of determination R^2 (R^2-based). The number of neighbors tested was: 2, 3, 4, 5, 6, 7, 8, 10, 12, and 14. The kNN model was then applied to the left-out external test to obtain the toxicity predictions. The procedure was applied 100 times for various external set and CV ratios. The ratios used were the same for the External set and CV: 0.05, 0.1, 0.15, 0.2, 0.25, 0.3. The CV for the hybrid model followed the same procedure but with the reduced variation in external set and CV ratios to decrease the computational time. The best and worst-performing ratios in the CV procedure for the standard models were selected as the external set ratios (0.1 and 0.2 for the descriptor-based and 0.2 and 0.3 for the fingerprint-based models. The middle values were considered for the CV, leading to 0.2 as the CV ratio.

Tables S10 and S12 summarize the cross-validation (CV) results of the standard kNN models: the most frequently appearing number of neighbors giving the highest score (R^2 or Spr_m) and the average values of R^2 and Spr coefficients. Tables S11 and S13 show the performance of the models when they are applied to the external left-out test set, which was not used for the CV procedure.

The results led to selection of the number of neighbors for the final models. The predictions made by the final models were used for evaluation by the knowledge rule metric and comparison with the performance of the hybrid models using the prior knowledge (See Tables S14 and S15).

The descriptors, fingerprints and Tanimoto similarity were calculated with the help of the open-source cheminformatics tool RDKit[215]. The RDKit Descriptors module calculated 200 descriptors, the removal of descriptors having zero values for all the molecules resulted in 93 descriptors used as molecular vectors. The Fingerprints used in this work were 1024-bit Morgan fingerprints with a radius of 2. For the hybrid model H1 with the variable selection as well as for the prior rules-based metric H4 additional descriptors not calculated by the RDKit (GATS1p, AATSC0p, SHBd, maxHBint2, ETA_dEpsilon_A, Mi, GATS1i, ETA_Alpha, ETA_EtaP_B) were computed with the help of PaDELPy[216]. Table S16 gives the full names of the descriptors used for the rules-based metric H4.

The estimation of toxicity was performed by Eq. (1)[217,218] for the case of the standard fingerprint-based models with the Tanimoto similarity and as an average of the neighbors' toxicity values for the final standard descriptor-based models and all the hybrid models. In the context of this work, hybrid models are the kNN models developed using prior knowledge[1]. No significant difference was observed if the average or similarity weighted approach was used; thus, the average approach was used for most of the models.

$$y_{pre,i} = \sum_{l=1}^{k} \frac{S_{i,l}}{\sum_{j=1}^{k} S_{i,j}} * y_{l,db} \quad (1)$$

where $y_{pre,i}$ is the prediction of the property of molecule *I*, $S_{i,l}$ is a similarity value between molecule *i* for which property predictions are sought and a molecule *l*, found in

a database, for which the desired property value $y_{l,db}$ is available, and k is the number of the similar neighbors used in the prediction.

Manhattan distance was computed by Eq.(2)[1]:

$$d_M = \sum_{i=1}^{n} |x_i - y_i| \qquad (2)$$

where x_i, y_i – i -th descriptors of molecular vectors x and y.

Use of prior knowledge (general schemes)

The prior knowledge could be applied before, during, and after the actual kNN (or other ML data science) approach[1]. For example, knowledge can be used to preprocess the data or select the model predictor variables before the modeling. During the data preprocessing, a rule identified in the literature can be applied to remove some molecules exhibiting deviance from the rule behavior The predictor (variable) selection can be performed based on the importance of molecular indicators and properties learned from the knowledge extraction. The initial set of descriptors can be reduced to a few more strongly correlated with the toxicity, for example. Another option is to cluster the data following the knowledge rules (e.g., based on the MoA or chemical class) and build a model for every cluster.

Different schemes presenting examples of how the knowledge can be incorporated into the predictive models are shown in Figure S5. Scheme 1 (Figure S5a) depicts an example where the prior knowledge model's (PKM), e.g., QSAR, error information, is used as an input for the data science model. Scheme 1 is a suitable form when a relatively good PKM exists but needs to be fine-tuned; its linear form is not good for all types of estimates and/or it does not consider some important parameters.

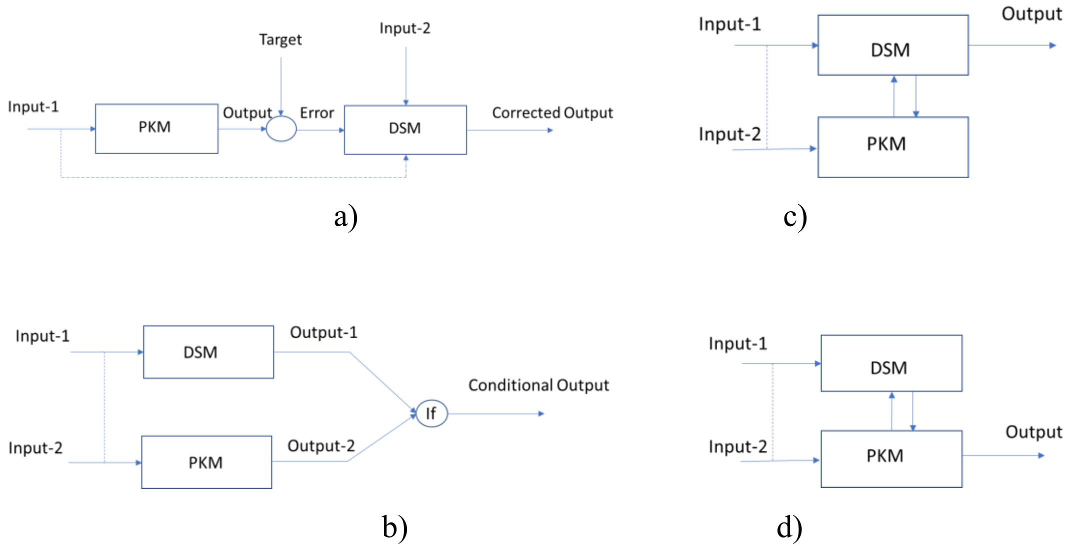

Figure S5. Examples of knowledge incorporation schemes. DSM: data science model, PKM: prior knowledge model

In the scheme 2 (Figure S5b), the predictions computed by either standard (DSM) or PKM are selected as final toxicity predicted values based on a conditional rule or rules retrieved from the knowledge base. Scheme 2 might be relevant when a good PKM model exists; however, it is weak in some areas of the variable domain. Another alternative is that PKM safeguards for alerts, etc., a good DSM (standard data science model) (this can also be categorized as post-processing).

Scheme 3 implies the use of the PKM to guide the DSM prediction or vice versa. Scheme 3 (Figure S5c) is a suitable form when the DSM uses PKM results to impose monotonicity, alerts, exceptional cases, etc. Scheme 4 (Figure S5d) could be used when a good PKM model exists, but some of its parameters (e.g., constants) can be fine-tuned.

The knowledge can also be used after the results of the models are obtained. Among the possible options are model validation/selection/adjustment based on a prior knowledge-based set of rules, extraction of new rules to complement the prior knowledge,

and interpretation of the results. The set of rules established from prior knowledge can provide a new dimension for the evaluation of the model performance, additionally to standard metrics of model accuracy[1].

Table S10. Average results of cross validation procedure of the descriptors-based models. N: most frequently appearing number of the neighbors giving the best Spearman rank-order correlation coefficient Spr (Spearman-based) or coefficient of determination R^2 (R^2-based). MARE: Mean Absolute Relative Error. Standard deviation is given in parenthesis.

**CV ratio**	**0.05**	**0.1**	**0.15**	**0.2**	**0.25**	**0.3**
Spearman-based						
N	6	8	5	5	7	6
Spr	0.954 (0.005)	0.955 (0.005)	0.955 (0.006)	0.955 (0.005)	0.957 (0.008)	0.955 (0.005)
MARE	0.955 (0.465)	0.933 (0.455)	0.960 (0.503)	0.946 (0.490)	0.973 (0.527)	0.933 (0.464)
R^2-based						
N	6	2	5	5	5	8
R^2	0.873 (0.016)	0.874 (0.015)	0.875 (0.015)	0.874 (0.016)	0.880 (0.019)	0.875 (0.016)
MARE	0.942 (0.444)	0.925 (0.440)	0.964 (0.485)	0.940 (0.471)	0.983 (0.524)	0.946 (0.451)

Table S11. Results of predictions for external test set made by the descriptors-based models (average values for different CV ratios are shown). N: most frequently appearing number of the neighbors giving the best Spearman rank-order correlation coefficient Spr (Spearman-based) or coefficient of determination R2 (R2-based). MARE: Mean Absolute Relative Error.

**External set ratio**	**0.05**	**0.1**	**0.15**	**0.2**	**0.25**	**0.3**
Spearman-based						
N	5	5	4	4	3	4
Spr	0.947	0.950	0.948	0.953	0.942	0.946
R^2	0.825	0.840	0.860	0.857	0.848	0.834
MARE	1.426	0.619	0.917	0.784	0.809	1.089
R^2-based						
N	5	2	5	4	5	6
R^2	0.830	0.815	0.855	0.857	0.850	0.845
Spr	0.947	0.937	0.947	0.953	0.945	0.948
MARE	1.577	0.656	0.831	0.755	0.777	1.073

Table S12. Average results of cross validation procedure of fingerprint-based models N: most frequently appearing number of the neighbors giving the best Spearman rank-order correlation coefficient Spr (Spearman-based) or coefficient of determination R2 (R2-based). MARE: Mean Absolute Relative Error. Standard deviation is given in parenthesis.

**CV ratio**	**0.05**	**0.1**	**0.15**	**0.2**	**0.25**	**0.3**
Spearman-based						
N	12	12	12	14	14	12
Spr	0.873 (0.011)	0.880 (0.012)	0.873 (0.011)	0.861 (0.008)	0.860 (0.009)	0.860 (0.008)
MARE	3.054 (2.458)	3.085 (3.083)	3.059 (2.411)	3.088 (1.606)	3.135 (1.662)	3.264 (1.631)
R^2-based						
N	2	5	7	8	7	7
R^2	0.755 (0.018)	0.767 (0.020)	0.755 (0.018)	0.730 (0.013)	0.731 (0.014)	0.731 (0.014)
MARE	3.339 (2.679)	3.085 (3.083)	3.086 (2.376)	3.145 (1.584)	3.234 (1.659)	3.174 (1.567)

Table S13. Results of predictions for external test set by fingerprint-based models (average values for different CV ratios are shown) N: most frequently appearing number of the neighbors giving the best Spearman rank-order correlation coefficient Spr (Spearman-based) or coefficient of determination R2 (R2-based). MARE: Mean Absolute Relative Error.

**External set ratio**	**0.05**	**0.1**	**0.15**	**0.2**	**0.25**	**0.3**
Spearman-based						
N	14	14	12	12	14	12
Spr	0.863	0.854	0.854	0.855	0.850	0.841
R^2	0.718	0.732	0.717	0.731	0.715	0.696
MARE	2.476	4.726	2.272	2.559	3.095	3.452
R^2-based						
N	7	2	5	12	7	6
R^2	0.716	0.707	0.721	**0.732**	0.699	**0.687**
Spr	0.853	0.842	0.853	0.855	0.840	0.834
MARE	2.551	5.832	2.358	2.762	3.089	3.299

Table S14. Average results of cross validation procedure hybrid models. N: most frequently appearing number of the neighbors giving the best Spearman rank-order correlation coefficient Spr (Spearman-based) or coefficient of determination R2 (R2-based). MARE: Mean Absolute Relative Error. Standard deviation is given in parenthesis.

	**Descriptor -based**		**Fingerprints-based**	
	**DESC_H0**	**DESC_H1**	**FPN_H2**	**FPN_H3**
CV ratio	**0.2**	**0.2**	**0.2**	**0.2**
Spearman-based				
N	3	2	2	12
Spr	0.980 (0.002)	0.977 (0.004)	0.836 (0.016)	0.792 (0.019)
MARE	0.877 (0.665)	0.662 (0.459)	3.627 (2.302)	2.996 (1.408)
R^2-based				
N	3	2	7	14
R^2	0.950 (0.005)	0.925 (0.011)	0.582 (0.048)	0.594 (0.033)
MARE	0.768 (0.567)	0.640 (0.440)	3.568 (2.311)	2.893 (1.368)

Table S15. Results of predictions by hybrid models for external test set. N: most frequently appearing number of the neighbors giving the best Spearman rank-order correlation coefficient Spr (Spearman-based) or coefficient of determination R2 (R2-based). MARE: Mean Absolute Relative Error.

	**Descriptor -based**				**Fingerprints-based**			
	**DESC_H0**		**DESC_H1**		**FPN_H2**		**FPN_H3**	
External set ratio	**0.1**	**0.2**	**0.1**	**0.2**	**0.2**	**0.3**	**0.2**	**0.3**
Spearman-based								
N	3	3	2	2	12	2	14	12
Spr	0.982	0.976	0.977	0.982	0.810	0.790	0.757	0.763
R2	0.949	0.949	0.886	0.934	0.486	0.451	0.506	0.528
MARE	0.747	0.697	0.526	0.206	2.027	2.397	2.291	2.979
R^2-based								
N	7	3	2	2	12	7	14	14
R^2	0.939	0.949	0.886	0.934	0.486	0.474	0.506	0.532
Spr	0.976	0.976	0.977	0.982	0.810	0.796	0.757	0.764
MARE	1.28	0.697	0.526	0.206	2.027	2.189	2.291	3.143

Table S16. Names of the descriptors used in hybridization H4.

Descriptor	Full names[215,216]
LogP (rdkit)	MolLogP, octanol−water partition coefficient
MR (rdkit)	MolMR, molar refractivity
GATS1p (padel)	Geary autocorrelation - lag 1 / weighted by polarizabilities
AATSC0p (padel)	Average centered Broto-Moreau autocorrelation - lag 0 / weighted by polarizabilities
TPSA (rdkit)	Topological polar surface area
SHBd	Sum of E-States for (strong) hydrogen bond donors
maxHBint2	Maximum E-State descriptors of strength for potential Hydrogen Bonds of path length 2
ETA_dEpsilon_A	A measure of contribution of unsaturation and electronegative atom count
Mi (padel)	Mean first ionization potentials (scaled on carbon atom)
GATS1i (padel)	Geary autocorrelation - lag 1 / weighted by first ionization potential
MW (rdkit)	ExactMolWt, Molecular weight
ETA_Alpha (padel)	Sum of alpha values of all non-hydrogen vertices of a molecule
ETA_EtaP_B (padel)	Branching index EtaB relative to molecular size

S4. Analysis of the outlier detection for the hybrid model H0.

For the hybrid model H0 an outlier detection hypothesis based on single descriptor MW was tested[1]. The idea was to use the visual (Figure S6) and Spearman coefficient (Spr)-based inspection of molecules which could be potential outliers. According to prior knowledge, the toxicity of a chemical increases with an increase in its MW. As seen in Figure S6, a correlation between the MW and toxicity values can be observed, the Spearman value being 0.26. However, the correlation seems to be stronger for the molecules with MW<300 g/mol. The removal of chemicals with MW>300 increases the Spr to 0.31 (Table S17). The computed Spr value for the 187 removed molecules showed the opposite trend for these compounds (Spr = -0.36). The Spr coefficients between the toxicity and other relevant for the aquatic toxicity descriptor values are presented in Table S17. It can be seen that the MW-based outliers follow some of the identified rules (involving logP, GATS1p, AATSC0p, TPSA, ETA_dEpsilon_A, GATS1i, nC methoxy) better than the rest of the dataset molecules. At the same time, the Spr correlation deteriorates for the descriptors MR, SHBd, maxHBint2, MW, ETA_Alpha, nC amines and nN-CH3. Further examination of the outliers (Table S18) showed that the outlier set consists of highly hydrophobic, toxic (T+PT) compounds (logP > 4) and chemicals with very low toxicity (NT). It should be noted that according to ECHA "Guidance on Information Requirements and Chemical Safety Assessment"[219], for certain lipophilic substances (with a Log Kow > 4) acute toxicity may not occur at the limit of the water solubility of the substance tested leading to measurement problems. This can be a reason for erroneous values in the set of outliers arising from estimations of LC50 values.

The outliers also show the increased MR, TPSA and ETA_Alpha values compared to the remaining molecules of the dataset. The group of toxic compounds consists of high MW amino alcohols, amino ethers and a few diols and triols. The hydrophilic moiety such as functionalities

like esters, aliphatic ethers, branching and higher oxygen content are reported to reduce toxicity[151], which might explain the reduced toxicity values of the nontoxic compounds. Furthermore, non-toxic compounds exhibit larger TPSA, which together with larger size (higher MW and ETA_Alpha) might hinder the ability of molecules to permeate the cells of the living organisms. The analysis could be used for further division of the dataset and construction of more local models (e.g., for high MW amino alcohols, amino ethers, diols and triols).

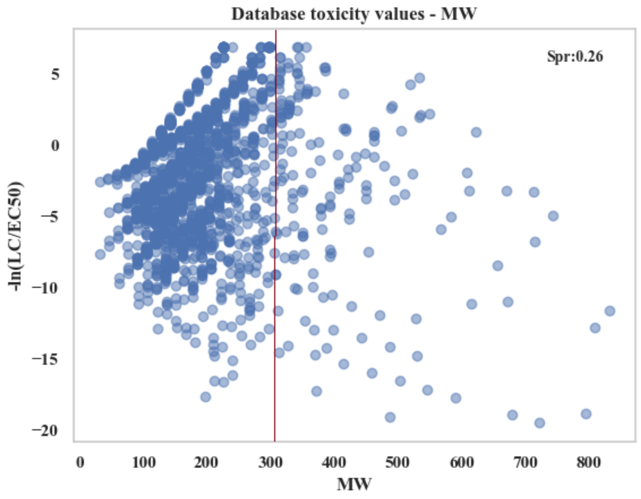

Figure S6. Correlation of dataset toxicity (-ln(LC50/EC50)) values with molecular weight.

Table S17. Correlation between toxicity and relevant descriptors based on Spearman coefficient (Spr) for the original data set, the 187 MW-based outliers and the dataset without the outliers (highlighted values indicate Spr changes higher than +/- 0.05 and descriptors with “*” refer to descriptors applicable only to certain classes of molecules).

Descriptor	Spr of original dataset	Spr for 187 outliers	Spr for the rest of the dataset
LogP (rdkit)	0.86	0.91	0.87
MR (rdkit)	0.46	**0.03**	**0.56**
GATS1p (padel)	-0.54	**-0.78**	-0.52
AATSC0p (padel)	0.81	**0.93**	0.81
TPSA (rdkit)	-0.61	**-0.80**	**-0.67**
SHBd	-0.5	**-0.33**	-0.54
maxHBint2	-0.17	**0.04**	-0.19
ETA_dEpsilon_A	-0.89	**-0.95**	-0.91
Mi (padel)	-0.66	-0.66	-0.67
GATS1i (padel)	-0.54	**-0.77**	-0.53
MW (rdkit)	0.26	**-0.36**	0.31
ETA_Alpha (padel)	0.39	**-0.13**	**0.47**
ETA_EtaP_B (padel)	-0.05	-0.1	-0.03
nC amines primary & secondary* (586 molecules)	0.59	**0.18** (43 molecules)	0.57 (543 molecules)
nC methoxy* (218 molecules)	0.06	**0.19** (17 molecules)	**0.22** (201 molecules)
nN-CH3* (78 molecules)	-0.45	**-0.32** (4 molecules)	-0.47 (74 molecules)

Table S18. Mean descriptor values (with standard deviation) for the set of 187 outliers (divided into T+PT and NT categories) and the rest of the dataset (highlighted values indicate noticeable differences for the descriptors between the investigated subsets of outliers).

Descriptor	Toxic outliers, T+PT (n=49)	Non-toxic outliers NT (n=138)	Rest of the dataset after the removal of outliers
-ln(LC50/EC50) dataset	**3.57 (1.33)**	**-15.75 (5.76)**	**-10.92 (4.13)**
LogP (rdkit)	**5.75 (0.84)**<br>**All >4**	**2.43 (2.26)**	**2.00 (2.04)**
MR (rdkit)	**108.14 (13.96)**	**114.90 (30.21)**	**49.85 (16.94)**
GATS1p (padel)	1.60 (0.05)	1.78 (0.13)	1.70 (0.16)
AATSC0p (padel)	0.21 (0.002)	0.20 (0.006)	0.20 (0.01)
TPSA (rdkit)	**41.11 (15.08)**	**85.95 (38.51)**	**28.82 (22.47)**
SHBd	0.69 (0.41)	1.01 (0.78)	0.56 (0.61)
maxHBint2	0.05 (0.33)	0.02 (0.22)	0.11 (0.63)
ETA_dEpsilon_A	0.03 (0.01)	0.09 (0.04)	0.06 (0.05)
Mi (padel)	7.72 (0.01)	7.74 (0.02)	7.76 (0.05)
GATS1i (padel)	1.66 (0.08)	1.87 (0.15)	1.80 (0.24)
MW (rdkit)	**349.44 (52.60)**	**423.23 (121.50)**	**165.34 (53.52)**
ETA_Alpha (padel)	**11.81 (1.65)**	**13.34 (3.68)**	**5.42 (1.84)**
ETA_EtaP_B (padel)	0.006 (0.007)	0.008 (0.009)	0.02 (0.02)
Representative classes	Amino alcohols, amino ethers, diols and triols	Compounds with amino, alcohol and ether groups in the same molecule	Alcohols, amines, ethers

The classification metrics of the 187 outliers for the selected standard and hybrid models (Table S19) showed that their performance (i.e., based on classification and regression metrics) for the outliers is worse compared to the values presented for the whole dataset (Table 4 in the main text). The hybrid model H1 based on the descriptor selection shows the best results. Moreover, models have a reduced performance with regards to toxic compounds compared to the nontoxic molecules (Table S20). It should, however, be noted, that the performance metrics presented in Tables S19 and S20 (as well as in Table 4) are based on the dataset values, which are subjected to uncertainty.

Table S19. Performance of the selected models for the set of 187 MW-based outliers (highlighted values indicate the best performing model).

Model	Classification metrics			Regression
	Accuracy	Precision	Recall	Spr_m
DESC_5	0.65	0.82	0.82	0.72
DESC_H1_2	**0.68**	0.84	0.85	**0.93**
FPN_2	0.48	0.74	0.78	0.78
FPN_H2_7	0.51	0.74	0.59	0.58
FPN_H3_12	0.32	0.59	0.57	0.28

Table S20. Performance of the selected models for the set of 187 MW-based outliers divided into sets of toxic (T+PT) and nontoxic (NT) molecules (highlighted values indicate the best performing model).

Model	Classification (Accuracy)		Regression (Spr_m)	
	T+PT	NT	T+PT	NT
DESC_5	0.50	0.93	0.35	0.54
DESC_H1_2	**0.53**	**0.96**	**0.71**	**0.85**
FPN_2	0.23	**0.97**	0.28	0.74
FPN_H2_7	0.43	0.67	0.21	0.42
FPN_H3_12	0.13	0.72	0.28	0.31

REFERENCES

(1) Shavalieva, G. Environmental , Health , and Safety Assessment of Chemical Alternatives during Early Process Design : The Role of Predictive Modeling and Streamlined Techniques, PhD thesis, Chalmers University of Technology, Sweden, 2022.

(2) Howard, P. H.; Muir, D. C. G. Identifying New Persistent and Bioaccumulative Organics among Chemicals in Commerce. III: Byproducts, Impurities, and Transformation Products. *Environ. Sci. Technol.* **2013**, *47*, 5259–5266. https://doi.org/10.1021/es4004075.

(3) Van Eck, N. J.; Waltman, L. *VOSviewer Manual*; 2013.

(4) Honnibal, M. Inroducing spaCy https://explosion.ai/blog/introducing-spacy (accessed 2021 -07 -18).

(5) Boudin, F. Pke: An Open Source Python-Based Keyphrase Extraction Toolkit. *COLING 2016 - 26th Int. Conf. Comput. Linguist. Proc. COLING 2016 Syst. Demonstr.* **2016**, 69–73.

(6) Wan, X.; Xiao, J. CollabRank: Towards a Collaborative Approach to Single-Document Keyphrase Extraction. *Coling 2008 - 22nd Int. Conf. Comput. Linguist. Proc. Conf.* **2008**, *1*, 969–976.

(7) Ellison, C. M.; Cronin, M. T. D.; Madden, J. C.; Schultz, T. W. Definition of the Structural Domain of the Baseline Non-Polar Narcosis Model for Tetrahymena Pyriformis. *SAR QSAR Environ. Res.* **2008**, *19*, 751–783. https://doi.org/10.1080/10629360802550366.

(8) Tan, N.-X.; Li, P.; Rao, H.-B.; Li, Z.-R.; Li, X.-Y. Prediction of the Acute Toxicity of Chemical Compounds to the Fathead Minnow by Machine Learning Approaches. *Chemom.*

Intell. Lab. Syst. **2010**, *100*, 66–73. https://doi.org/10.1016/j.chemolab.2009.11.002.

(9) Alves, V. M.; Muratov, E. N.; Capuzzi, S. J.; Politi, R.; Low, Y.; Braga, R. C.; Zakharov, A. V.; Sedykh, A.; Mokshyna, E.; Farag, S.; Andrade, C. H.; Kuz'Min, V. E.; Fourches, D.; Tropsha, A. Alarms about Structural Alerts. *Green Chem.* **2016**, *18*, 4348–4360. https://doi.org/10.1039/c6gc01492e.

(10) Giuseppina, G.; Thomas, F.; Anna, L.; Antonio, C.; Emilio, B. A New QSAR Model for Acute Fish Toxicity Based on Mined Structural Alerts. *J. Toxicol. Risk Assess.* **2019**, *5*, 1–8. https://doi.org/10.23937/2572-4061.1510016.

(11) Morrall, D. D.; Belanger, S. E.; Dunphy, J. C. Acute and Chronic Aquatic Toxicity Structure-Activity Relationships for Alcohol Ethoxylates. *Ecotoxicol. Environ. Saf.* **2003**, *56*, 381–389. https://doi.org/10.1016/S0147-6513(02)00088-X.

(12) Cassotti, M.; Ballabio, D.; Consonni, V.; Mauri, A.; Tetko, I. V.; Todeschini, R. Prediction of Acute Aquatic Toxicity toward Daphnia Magna by Using the GA- k NN Method. *Altern. to Lab. Anim.* **2014**, *42*, 31–41. https://doi.org/10.1177/026119291404200106.

(13) Stanton, D. T.; Dimitrov, S.; Grancharov, V.; Mekenyan, O. G. Charged Partial Surface Area (CPSA) Descriptors QSAR Applications. *SAR QSAR Environ. Res.* **2002**, *13*, 341–351. https://doi.org/10.1080/10629360290002811.

(14) Finizio, A.; Di Nica, V.; Rizzi, C.; Villa, S. A Quantitative Structure-Activity Relationships Approach to Predict the Toxicity of Narcotic Compounds to Aquatic Communities. *Ecotoxicol. Environ. Saf.* **2020**, *190*, 110068. https://doi.org/10.1016/j.ecoenv.2019.110068.

(15) Voutchkova, A. M.; Kostal, J.; Steinfeld, J. B.; Emerson, J. W.; Brooks, B. W.; Anastas, P.; Zimmerman, J. B. Towards Rational Molecular Design: Derivation of Property Guidelines for Reduced Acute Aquatic Toxicity. *Green Chem.* **2011**, *13*, 2373–2379. https://doi.org/10.1039/c1gc15651a.

(16) Li, F.; Fan, D.; Wang, H.; Yang, H.; Li, W.; Tang, Y.; Liu, G. In Silico Prediction of Pesticide Aquatic Toxicity with Chemical Category Approaches. *Toxicol. Res. (Camb).* **2017**, *6*, 831–842. https://doi.org/10.1039/c7tx00144d.

(17) Yen, J.-H.; Lin, K.-H.; Wang, Y.-S. Acute Lethal Toxicity of Environmental Pollutants to Aquatic Organisms. *Ecotoxicol. Environ. Saf.* **2002**, *52*, 113–116. https://doi.org/10.1006/eesa.2002.2167.

(18) Seth, A.; Roy, K. QSAR Modeling of Algal Low Level Toxicity Values of Different Phenol and Aniline Derivatives Using 2D Descriptors. *Aquat. Toxicol.* **2020**, *228*, 105627. https://doi.org/10.1016/j.aquatox.2020.105627.

(19) Khan, K.; Roy, K. Ecotoxicological QSAR Modelling of Organic Chemicals against Pseudokirchneriella Subcapitata Using Consensus Predictions Approach. *SAR QSAR Environ. Res.* **2019**, *30*, 665–681. https://doi.org/10.1080/1062936X.2019.1648315.

(20) Martin, T. M.; Grulke, C. M.; Young, D. M.; Russom, C. L.; Wang, N. Y.; Jackson, C. R.; Barron, M. G. Prediction of Aquatic Toxicity Mode of Action Using Linear Discriminant and Random Forest Models. *J. Chem. Inf. Model.* **2013**, *53*, 2229–2239. https://doi.org/10.1021/ci400267h.

(21) Khan, K.; Kar, S.; Sanderson, H.; Roy, K.; Leszczynski, J. Ecotoxicological Modeling,

Ranking and Prioritization of Pharmaceuticals Using QSTR and i-QSTTR Approaches: Application of 2D and Fragment Based Descriptors. *Mol. Inform.* **2019**, *38*. https://doi.org/10.1002/minf.201800078.

(22) Toropova, A. P.; Toropov, A. A.; Veselinović, A. M.; Veselinović, J. B.; Leszczynska, D.; Leszczynski, J. Monte Carlo–Based Quantitative Structure–Activity Relationship Models for Toxicity of Organic Chemicals to Daphnia Magna. *Environ. Toxicol. Chem.* **2016**, *35*, 2691–2697. https://doi.org/10.1002/etc.3466.

(23) Stoyanova-Slavova, I. B.; Slavov, S. H.; Pearce, B.; Buzatu, D. A.; Beger, R. D.; Wilkes, J. G. Partial Least Square and K-Nearest Neighbor Algorithms for Improved 3D Quantitative Spectral Data-Activity Relationship Consensus Modeling of Acute Toxicity. *Environmental Toxicology and Chemistry*. 2014, pp 1271–1282. https://doi.org/10.1002/etc.2534.

(24) Bajot, F.; Cronin, M. T. D.; Roberts, D. W.; Schultz, T. W. Reactivity and Aquatic Toxicity of Aromatic Compounds Transformable to Quinone-Type Michael Acceptors. *SAR QSAR Environ. Res.* **2011**, *22*, 51–65. https://doi.org/10.1080/1062936X.2010.528449.

(25) Slavov, S.; Gini, G.; Benfenati, E. QSAR Trout Toxicity Models on Aromatic Pesticides. *J. Environ. Sci. Heal. - Part B Pestic. Food Contam. Agric. Wastes* **2008**, *43*, 633–637. https://doi.org/10.1080/03601230802352658.

(26) Cronin, M. T. D.; Bowers, G. S.; Sinks, G. D.; Schultz, T. W. Structure-Toxicity Relationships for Aliphatic Compounds Encompassing a Variety of Mechanisms of Toxic Action to Vibrio Fischeri. *SAR QSAR Environ. Res.* **2000**, *11*, 301–312. https://doi.org/10.1080/10629360008033237.

(27) DeWeese, A. D.; Schultz, T. W. Structure-Activity Relationships for Aquatic Toxicity ToTetrahymena: Halogen-Substituted Aliphatic Esters. *Environ. Toxicol.* **2001**, *16*, 54–60. https://doi.org/10.1002/1522-7278(2001)16:1<54::AID-TOX60>3.0.CO;2-M.

(28) Yarbrough, J. W.; Schultz, T. W. Abiotic Sulfhydryl Reactivity: A Predictor of Aquatic Toxicity for Carbonyl-Containing α,β-Unsaturated Compounds. *Chem. Res. Toxicol.* **2007**, *20*, 558–562. https://doi.org/10.1021/tx600344a.

(29) Roy, K.; Ghosh, G. QSTR with Extended Topochemical Atom Indices. 2. Fish Toxicity of Substituted Benzenes. *J. Chem. Inf. Comput. Sci.* **2004**, *44*, 559–567. https://doi.org/10.1021/ci0342066.

(30) Schultz, T. W.; Cronin, M. T. D.; Netzeva, T. I.; Aptula, A. O. Structure−Toxicity Relationships for Aliphatic Chemicals Evaluated with Tetrahymena Pyriformis. *Chem. Res. Toxicol.* **2002**, *15*, 1602–1609. https://doi.org/10.1021/tx025589p.

(31) Colombo, A.; Benfenati, E.; Karelson, M.; Maran, U. The Proposal of Architecture for Chemical Splitting to Optimize QSAR Models for Aquatic Toxicity. *Chemosphere* **2008**, *72*, 772–780. https://doi.org/10.1016/j.chemosphere.2008.03.016.

(32) Zvinavashe, E.; Van Den Berg, H.; Soffers, A. E. M. F.; Vervoort, J.; Freidig, A.; Murk, A. J.; Rietjens, I. M. C. M. QSAR Models for Predicting in Vivo Aquatic Toxicity of Chlorinated Alkanes to Fish. *Chem. Res. Toxicol.* **2008**, *21*, 739–745. https://doi.org/10.1021/tx700367c.

(33) Jana, G.; Pal, R.; Sural, S.; Chattaraj, P. K. Quantitative Structure-toxicity Relationship: An “in Silico Study” Using Electrophilicity and Hydrophobicity as Descriptors. *Int. J. Quantum*

Chem. **2020**, *120*, 1–12. https://doi.org/10.1002/qua.26097.

(34) Smiesko, M.; Benfenati, E. Predictive Models for Aquatic Toxicity of Aldehydes Designed for Various Model Chemistries. *J. Chem. Inf. Comput. Sci.* **2004**, *44*, 976–984. https://doi.org/10.1021/ci034219j.

(35) Khan, K.; Baderna, D.; Cappelli, C.; Toma, C.; Lombardo, A.; Roy, K.; Benfenati, E. Ecotoxicological QSAR Modeling of Organic Compounds against Fish: Application of Fragment Based Descriptors in Feature Analysis. *Aquat. Toxicol.* **2019**, *212*, 162–174. https://doi.org/10.1016/j.aquatox.2019.05.011.

(36) Papa, E.; Battaini, F.; Gramatica, P. Ranking of Aquatic Toxicity of Esters Modelled by QSAR. *Chemosphere* **2005**, *58*, 559–570. https://doi.org/10.1016/j.chemosphere.2004.08.003.

(37) Gramatica, P.; Cassani, S.; Chirico, N. QSARINS-Chem: Insubria Datasets and New QSAR/QSPR Models for Environmental Pollutants in QSARINS. *J. Comput. Chem.* **2014**, *35*, 1036–1044. https://doi.org/10.1002/jcc.23576.

(38) Fisk, P. R.; Wildey, R. J.; Girling, A. E.; Sanderson, H.; Belanger, S. E.; Veenstra, G.; Nielsen, A.; Kasai, Y.; Willing, A.; Dyer, S. D.; Stanton, K. Environmental Properties of Long Chain Alcohols. Part 1: Physicochemical, Environmental Fate and Acute Aquatic Toxicity Properties. *Ecotoxicol. Environ. Saf.* **2009**, *72*, 980–995. https://doi.org/10.1016/j.ecoenv.2008.09.025.

(39) Belanger, S. E.; Rawlings, J. M.; Stackhouse, R. Advances in Understanding the Response of Fish to Linear Alcohols in the Environment. *Chemosphere* **2018**, *206*, 539–548.

https://doi.org/10.1016/j.chemosphere.2018.04.152.

(40) Schultz, T. W.; Netzeva, T. I.; Roberts, D. W.; Cronin, M. T. D. Structure-Toxicity Relationships for the Effects to Tetrahymena Pyriformis of Aliphatic, Carbonyl-Containing, α,β-Unsaturated Chemicals. *Chem. Res. Toxicol.* **2005**, *18*, 330–341. https://doi.org/10.1021/tx049833j.

(41) Böhme, A.; Laqua, A.; Schüürmann, G. Chemoavailability of Organic Electrophiles: Impact of Hydrophobicity and Reactivity on Their Aquatic Excess Toxicity. *Chem. Res. Toxicol.* **2016**, *29*, 952–962. https://doi.org/10.1021/acs.chemrestox.5b00398.

(42) Huang, C. P.; Wang, Y. J.; Chen, C. Y. Toxicity and Quantitative Structure-Activity Relationships of Nitriles Based on Pseudokirchneriella Subcapitata. *Ecotoxicol. Environ. Saf.* **2007**, *67*, 439–446. https://doi.org/10.1016/j.ecoenv.2006.06.007.

(43) Chen, C. Y.; Kuo, K. L.; Fan, J. W. Toxicity of Propargylic Alcohols on Green Alga - Pseudokirchneriella Subcapitata. *J. Environ. Monit.* **2012**, *14*, 181–186. https://doi.org/10.1039/c1em10552c.

(44) Schultz, T. W.; Netzeva, T. I.; Cronin, M. T. D. Selection of Data Sets for QSARs: Analyses of Tetrahymena Toxicity from Aromatic Compounds. *SAR QSAR Environ. Res.* **2003**, *14*, 59–81. https://doi.org/10.1080/1062936021000058782.

(45) Netzeva, T. I.; Dearden, J. C.; Edwards, R.; Worgan, A. D. P.; Cronin, M. T. D. QSAR Analysis of the Toxicity of Aromatic Compounds to Chlorella Vulgaris in a Novel Short-Term Assay. *J. Chem. Inf. Comput. Sci.* **2004**, *44*, 258–265. https://doi.org/10.1021/ci034195g.

(46) Marzio, W. Di; Saenz, M. E. Quantitative Structure–Activity Relationship for Aromatic Hydrocarbons on Freshwater Fish. *Ecotoxicol. Environ. Saf.* **2004**, *59*, 256–262. https://doi.org/10.1016/j.ecoenv.2003.11.006.

(47) Laszlo, T.; Beteringhe, A. QSAR Studies Related to Toxicity of Aromatic Compounds on Tetrahymena Pyriformis. *QSAR Comb. Sci.* **2006**, *25*, 944–951. https://doi.org/10.1002/qsar.200630030.

(48) Lei, B.; Li, J.; Liu, H.; Yao, X. Accurate Prediction of Aquatic Toxicity of Aromatic Compounds Based on Genetic Algorithm and Least Squares Support Vector Machines. *QSAR Comb. Sci.* **2008**, *27*, 850–865. https://doi.org/10.1002/qsar.200760167.

(49) Su, Q.; Lu, W.; Du, D.; Chen, F.; Niu, B.; Chou, K.-C. Prediction of the Aquatic Toxicity of Aromatic Compounds to Tetrahymena Pyriformis through Support Vector Regression. *Oncotarget* **2017**, *8*, 49359–49369.

(50) Yang, S.; Wang, C. Study on Aromatic Hydrocarbons Toxicity to Chlorella Vulgaris Based on QSAR Model. *Indian J. Geo-Marine Sci.* **2017**, *46*, 678–685.

(51) Basak, S. C.; Grunwald, G. D.; Gute, B. D.; Balasubramanian, K.; Opitz, D. Use of Statistical and Neural Net Approaches in Predicting Toxicity of Chemicals. *J. Chem. Inf. Comput. Sci.* **2000**, *40*, 885–890. https://doi.org/10.1021/ci9901136.

(52) Toropov, A. A.; Schultz, T. W. Prediction of Aquatic Toxicity: Use of Optimization of Correlation Weights of Local Graph Invariants. *J. Chem. Inf. Comput. Sci.* **2003**, *43*, 560–567. https://doi.org/10.1021/ci025555n.

(53) Toropov, A. A.; Toropova, A. P. QSAR Modeling of Toxicity on Optimization of

Correlation Weights of Morgan Extended Connectivity. *J. Mol. Struct. THEOCHEM* **2002**, *578*, 129–134. https://doi.org/10.1016/S0166-1280(01)00695-9.

(54) González, M. P.; Helguera, A. M.; Cabrera, M. A. Quantitative Structure-Activity Relationship to Predict Toxicological Properties of Benzene Derivative Compounds. *Bioorganic Med. Chem.* **2005**, *13*, 1775–1781. https://doi.org/10.1016/j.bmc.2004.11.059.

(55) Castillo-Garit, J. A.; Marrero-Ponce, Y.; Escobar, J.; Torrens, F.; Rotondo, R. A Novel Approach to Predict Aquatic Toxicity from Molecular Structure. *Chemosphere* **2008**, *73*, 415–427. https://doi.org/10.1016/j.chemosphere.2008.05.024.

(56) Gong, Z.; Xia, B.; Zhang, R.; Zhang, X.; Fan, B. Quantitive Structure - Activity Relationship Study on Fish Toxicity of Substituted Benzenes. *QSAR Comb. Sci.* **2008**, *27*, 967–976. https://doi.org/10.1002/qsar.200710096.

(57) Katritzky, A. R.; Slavov, S. H.; Stoyanova-Slavova, I. S.; Kahn, I.; Karelson, M. Quantitative Structure-Activity Relationship (QSAR) Modeling of EC 50 of Aquatic Toxicities for Daphnia Magna. *J. Toxicol. Environ. Heal. - Part A Curr. Issues* **2009**, *72*, 1181–1190. https://doi.org/10.1080/15287390903091863.

(58) Gupta, S.; Basant, N.; Singh, K. P. Predicting Aquatic Toxicities of Benzene Derivatives in Multiple Test Species Using Local, Global and Interspecies QSTR Modeling Approaches. *RSC Adv.* **2015**, *5*, 71153–71163. https://doi.org/10.1039/C5RA12825K.

(59) Toropova, A. P.; Schultz, T. W.; Toropov, A. A. Building up a QSAR Model for Toxicity toward Tetrahymena Pyriformis by the Monte Carlo Method: A Case of Benzene Derivatives. *Environ. Toxicol. Pharmacol.* **2016**, *42*, 135–145.

https://doi.org/10.1016/j.etap.2016.01.010.

(60) Martínez-López, Y.; Barigye, S. J.; Martínez-Santiago, O.; Marrero-Ponce, Y.; Green, J.; Castillo-Garit, J. A. Prediction of Aquatic Toxicity of Benzene Derivatives Using Molecular Descriptor from Atomic Weighted Vectors. *Environ. Toxicol. Pharmacol.* **2017**, *56*, 314–321. https://doi.org/10.1016/j.etap.2017.10.006.

(61) Cassani, S.; Kovarich, S.; Papa, E.; Roy, P. P.; van der Wal, L.; Gramatica, P. Daphnia and Fish Toxicity of (Benzo)Triazoles: Validated QSAR Models, and Interspecies Quantitative Activity-Activity Modelling. *J. Hazard. Mater.* **2013**, *258–259*, 50–60. https://doi.org/10.1016/j.jhazmat.2013.04.025.

(62) Lee, P. Y.; Chen, C. Y. Toxicity and Quantitative Structure-Activity Relationships of Benzoic Acids to Pseudokirchneriella Subcapitata. *J. Hazard. Mater.* **2009**, *165*, 156–161. https://doi.org/10.1016/j.jhazmat.2008.09.086.

(63) Qin, W. C.; Su, L. M.; Zhang, X. J.; Qin, H. W.; Wen, Y.; Guo, Z.; Sun, F. T.; Sheng, L. X.; Zhao, Y. H.; Abraham, M. H. Toxicity of Organic Pollutants to Seven Aquatic Organisms: Effect of Polarity and Ionization. *SAR QSAR Environ. Res.* **2010**, *21*, 389–401. https://doi.org/10.1080/1062936X.2010.501143.

(64) Netzeva, T. I.; Schultz, T. W. QSARs for the Aquatic Toxicity of Aromatic Aldehydes from Tetrahymena Data. *Chemosphere* **2005**, *61*, 1632–1643. https://doi.org/10.1016/j.chemosphere.2005.04.040.

(65) Roy, K.; Das, R. N. QSTR with Extended Topochemical Atom (ETA) Indices 14 QSAR Modeling of Toxicity of Aromatic Aldehydes to Tetrahymena Pyriformis. *J. Hazard. Mater.*

2010, *183*, 913–922. https://doi.org/10.1016/j.jhazmat.2010.07.116.

(66) Louis, B.; Agrawal, V. K. QSAR Modeling of Aquatic Toxicity of Aromatic Aldehydes Using Artificial Neural Network (ANN) and Multiple Linear Regression (MLR). *J. Indian Chem. Soc.* **2011**, *88*, 99–107.

(67) Roy, K.; Ghosh, G. QSTR with Extended Topochemical Atom Indices. 3. Toxicity of Nitrobenzenes ToTetrahymena Pyriformis. *QSAR Comb. Sci.* **2004**, *23*, 99–108. https://doi.org/10.1002/qsar.200330864.

(68) Yan, X. F.; Xiao, H. M.; Gong, X. D.; Ju, X. H. Quantitative Structure-Activity Relationships of Nitroaromatics Toxicity to the Algae (Scenedesmus Obliguus). *Chemosphere* **2005**, *59*, 467–471. https://doi.org/10.1016/j.chemosphere.2005.01.085.

(69) Lin, K. H.; Jaw, C. G.; Yen, J. H.; Wang, Y. S. Molecular Connectivity Indices for Predicting Bioactivities of Substituted Nitrobenzene and Aniline Compounds. *Ecotoxicol. Environ. Saf.* **2009**, *72*, 1942–1949. https://doi.org/10.1016/j.ecoenv.2009.04.007.

(70) Bellifa, K.; Mekelleche, S. M. QSAR Study of the Toxicity of Nitrobenzenes to Tetrahymena Pyriformis Using Quantum Chemical Descriptors. *Arab. J. Chem.* **2016**, *9*, S1683–S1689. https://doi.org/10.1016/j.arabjc.2012.04.031.

(71) Dom, N.; Knapen, D.; Benoot, D.; Nobels, I.; Blust, R. Aquatic Multi-Species Acute Toxicity of (Chlorinated) Anilines: Experimental versus Predicted Data. *Chemosphere* **2010**, *81*, 177–186. https://doi.org/10.1016/j.chemosphere.2010.06.059.

(72) Wang, X.; Dong, Y.; Wang, L.; Han, S. Acute Toxicity of Substituted Phenols to Rana Japonica Tadpoles and Mechanism-Based Quantitative Structure–Activity Relationship

(QSAR) Study. *Chemosphere* **2001**, *44*, 447–455. https://doi.org/10.1016/S0045-6535(00)00198-3.

(73) Ren, S. Phenol Mechanism of Toxic Action Classification and Prediction: A Decision Tree Approach. *Toxicol. Lett.* **2003**, *144*, 313–323. https://doi.org/10.1016/S0378-4274(03)00236-4.

(74) Smieško, M.; Benfenati, E. Thermodynamic Descriptors Derived from Density Functional Theory Calculations in Prediction of Aquatic Toxicity. *J. Chem. Inf. Model.* **2005**, *45*, 379–385. https://doi.org/10.1021/ci049684a.

(75) Ertürk, M. D.; Saçan, M. T.; Novic, M.; Minovski, N. Quantitative Structure-Activity Relationships (QSARs) Using the Novel Marine Algal Toxicity Data of Phenols. *J. Mol. Graph. Model.* **2012**, *38*, 90–100. https://doi.org/10.1016/j.jmgm.2012.06.002.

(76) Dieguez-Santana, K.; Pham-The, H.; Villegas-Aguilar, P. J.; Le-Thi-Thu, H.; Castillo-Garit, J. A.; Casañola-Martin, G. M. Prediction of Acute Toxicity of Phenol Derivatives Using Multiple Linear Regression Approach for Tetrahymena Pyriformis Contaminant Identification in a Median-Size Database. *Chemosphere* **2016**, *165*, 434–441. https://doi.org/10.1016/j.chemosphere.2016.09.041.

(77) Abbasitabar, F.; Zare-Shahabadi, V. In Silico Prediction of Toxicity of Phenols to Tetrahymena Pyriformis by Using Genetic Algorithm and Decision Tree-Based Modeling Approach. *Chemosphere* **2017**, *172*, 249–259. https://doi.org/10.1016/j.chemosphere.2016.12.095.

(78) Tugcu, G.; Ertürk, M. D.; Saçan, M. T. On the Aquatic Toxicity of Substituted Phenols to

Chlorella Vulgaris: QSTR with an Extended Novel Data Set and Interspecies Models. *J. Hazard. Mater.* **2017**, *339*, 122–130. https://doi.org/10.1016/j.jhazmat.2017.06.027.

(79) Tugcu, G.; Saçan, M. T. A Multipronged QSAR Approach to Predict Algal Low-Toxic-Effect Concentrations of Substituted Phenols and Anilines. *J. Hazard. Mater.* **2018**, *344*, 893–901. https://doi.org/10.1016/j.jhazmat.2017.11.033.

(80) Yan, F.; Liu, T.; Jia, Q.; Wang, Q. Multiple Toxicity Endpoint–Structure Relationships for Substituted Phenols and Anilines. *Sci. Total Environ.* **2019**, *663*, 560–567. https://doi.org/10.1016/j.scitotenv.2019.01.362.

(81) Muhire, J.; Li, B. Q.; Zhai, H. L.; Li, S. S.; Mi, J. Y. A Simple Approach to the Toxicity Prediction of Anilines and Phenols Towards Aquatic Organisms. *Arch. Environ. Contam. Toxicol.* **2020**, *78*, 545–554. https://doi.org/10.1007/s00244-019-00703-z.

(82) Feng, H.; Chen, Y.; Yue, W.; Feng, C. The Molecular Topological Research on Acute Toxicities of Substituted Arenes to Aquatic Organisms. *IOP Conf. Ser. Earth Environ. Sci.* **2018**, *153*. https://doi.org/10.1088/1755-1315/153/2/022035.

(83) Ren, S. Modeling the Toxicity of Aromatic Compounds to Tetrahymena Pyriformis: The Response Surface Methodology with Nonlinear Methods. *J. Chem. Inf. Comput. Sci.* **2003**, *43*, 1679–1687. https://doi.org/10.1021/ci034046y.

(84) Zhu, M.; Ge, F.; Zhu, R.; Wang, X.; Zheng, X. A DFT-Based QSAR Study of the Toxicity of Quaternary Ammonium Compounds on Chlorella Vulgaris. *Chemosphere* **2010**, *80*, 46–52. https://doi.org/10.1016/j.chemosphere.2010.03.044.

(85) Jing, G.; Zhou, Z.; Zhuo, J. Quantitative Structure-Activity Relationship (QSAR) Study of

Toxicity of Quaternary Ammonium Compounds on Chlorella Pyrenoidosa and Scenedesmus Quadricauda. *Chemosphere* **2012**, *86*, 76–82. https://doi.org/10.1016/j.chemosphere.2011.09.021.

(86) Mazzatorta, P.; Smiesko, M.; Lo Piparo, E.; Benfenati, E. QSAR Model for Predicting Pesticide Aquatic Toxicity. *J. Chem. Inf. Model.* **2005**, *45*, 1767–1774. https://doi.org/10.1021/ci050247l.

(87) Toropov, A. A.; Benfenati, E. QSAR Models for Daphnia Toxicity of Pesticides Based on Combinations of Topological Parameters of Molecular Structures. *Bioorganic Med. Chem.* **2006**, *14*, 2779–2788. https://doi.org/10.1016/j.bmc.2005.11.060.

(88) Wang, Y.; Zheng, M.; Xiao, J.; Lu, Y.; Wang, F.; Lu, J.; Luo, X.; Zhu, W.; Jiang, H.; Chen, K. Using Support Vector Regression Coupled with the Genetic Algorithm for Predicting Acute Toxicity to the Fathead Minnow. *SAR QSAR Environ. Res.* **2010**, *21*, 559–570. https://doi.org/10.1080/1062936X.2010.502300.

(89) Zvinavashe, E.; Du, T.; Griff, T.; Berg, H. H. J. va. den; Soffers, A. E. M. F.; Vervoort, J.; Murk, A. J.; Rietjens, I. M. C. M. Quantitative Structure-Activity Relationship Modeling of the Toxicity of Organothiophosphate Pesticides to Daphnia Magna and Cyprinus Carpio. *Chemosphere* **2009**, *75*, 1531–1538. https://doi.org/10.1016/j.chemosphere.2009.01.081.

(90) Basant, N.; Gupta, S.; Singh, K. P. Predicting Aquatic Toxicities of Chemical Pesticides in Multiple Test Species Using Nonlinear QSTR Modeling Approaches. *Chemosphere* **2015**, *139*, 246–255. https://doi.org/10.1016/j.chemosphere.2015.06.063.

(91) He, L.; Xiao, K.; Zhou, C.; Li, G.; Yang, H.; Li, Z.; Cheng, J. Insights into Pesticide Toxicity

against Aquatic Organism: QSTR Models on Daphnia Magna. *Ecotoxicol. Environ. Saf.* **2019**, *173*, 285–292. https://doi.org/10.1016/j.ecoenv.2019.02.014.

(92) Galimberti, F.; Moretto, A.; Papa, E. Application of Chemometric Methods and QSAR Models to Support Pesticide Risk Assessment Starting from Ecotoxicological Datasets. *Water Res.* **2020**, *174*, 115583. https://doi.org/10.1016/j.watres.2020.115583.

(93) Jia, Q.; Liu, T.; Yan, F.; Wang, Q. Norm Index–Based QSAR Model for Acute Toxicity of Pesticides Toward Rainbow Trout. *Environ. Toxicol. Chem.* **2020**, *39*, 352–358. https://doi.org/10.1002/etc.4621.

(94) Marzo, M.; Lavado, G. J.; Como, F.; Toropova, A. P.; Toropov, A. A.; Baderna, D.; Cappelli, C.; Lombardo, A.; Toma, C.; Blázquez, M.; Benfenati, E. QSAR Models for Biocides: The Example of the Prediction of Daphnia Magna Acute Toxicity. *SAR QSAR Environ. Res.* **2020**, *31*, 227–243. https://doi.org/10.1080/1062936X.2019.1709221.

(95) Toropov, A. A.; Toropova, A. P.; Benfenati, E. QSAR Model for Pesticides Toxicity to Rainbow Trout Based on "Ideal Correlations." *Aquat. Toxicol.* **2020**, *227*, 105589. https://doi.org/10.1016/j.aquatox.2020.105589.

(96) Wang, J.; Yang, Y.; Huang, Y.; Zhang, X.; Huang, Y.; Qin, W. C.; Wen, Y.; Zhao, Y. H. Evaluation of Modes of Action of Pesticides to Daphnia Magna Based on QSAR, Excess Toxicity and Critical Body Residues. *Ecotoxicol. Environ. Saf.* **2020**, *203*, 111046. https://doi.org/10.1016/j.ecoenv.2020.111046.

(97) Yang, L.; Wang, Y.; Hao, W.; Chang, J.; Pan, Y.; Li, J.; Wang, H. Modeling Pesticides Toxicity to Sheepshead Minnow Using QSAR. *Ecotoxicol. Environ. Saf.* **2020**, *193*,

110352. https://doi.org/10.1016/j.ecoenv.2020.110352.

(98) Yang, L.; Wang, Y.; Chang, J.; Pan, Y.; Wei, R.; Li, J.; Wang, H. QSAR Modeling the Toxicity of Pesticides against Americamysis Bahia. *Chemosphere* **2020**, *258*, 127217. https://doi.org/10.1016/j.chemosphere.2020.127217.

(99) Kim, Y.; Choi, K.; Jung, J.; Park, S.; Kim, P. G.; Park, J. Aquatic Toxicity of Acetaminophen, Carbamazepine, Cimetidine, Diltiazem and Six Major Sulfonamides, and Their Potential Ecological Risks in Korea. *Environ. Int.* **2007**, *33*, 370–375. https://doi.org/10.1016/j.envint.2006.11.017.

(100) Singh, K. P.; Gupta, S.; Basant, N. QSTR Modeling for Predicting Aquatic Toxicity of Pharmacological Active Compounds in Multiple Test Species for Regulatory Purpose. *Chemosphere* **2015**, *120*, 680–689. https://doi.org/10.1016/j.chemosphere.2014.10.025.

(101) Sangion, A.; Gramatica, P. Ecotoxicity Interspecies QAAR Models from Daphnia Toxicity of Pharmaceuticals and Personal Care Products. *SAR QSAR Environ. Res.* **2016**, *27*, 781–798. https://doi.org/10.1080/1062936X.2016.1233139.

(102) Gramatica, P.; Papa, E.; Sangion, A. QSAR Modeling of Cumulative Environmental End-Points for the Prioritization of Hazardous Chemicals. *Environ. Sci. Process. Impacts* **2018**, *20*, 38–47. https://doi.org/10.1039/c7em00519a.

(103) Khan, K.; Benfenati, E.; Roy, K. Consensus QSAR Modeling of Toxicity of Pharmaceuticals to Different Aquatic Organisms: Ranking and Prioritization of the DrugBank Database Compounds. *Ecotoxicol. Environ. Saf.* **2019**, *168*, 287–297. https://doi.org/10.1016/j.ecoenv.2018.10.060.

(104) Serra, A.; Önlü, S.; Festa, P.; Fortino, V.; Greco, D. MaNGA: A Novel Multi-Niche Multi-Objective Genetic Algorithm for QSAR Modelling. *Bioinformatics* **2020**, *36*, 145–153. https://doi.org/10.1093/bioinformatics/btz521.

(105) Levet, A.; Bordes, C.; Clément, Y.; Mignon, P.; Chermette, H.; Marote, P.; Cren-Olivé, C.; Lantéri, P. Quantitative Structure-Activity Relationship to Predict Acute Fish Toxicity of Organic Solvents. *Chemosphere* **2013**, *93*, 1094–1103. https://doi.org/10.1016/j.chemosphere.2013.06.002.

(106) Levet, A.; Bordes, C.; Clément, Y.; Mignon, P.; Morell, C.; Chermette, H.; Marote, P.; Lantéri, P. Acute Aquatic Toxicity of Organic Solvents Modeled by QSARs. *J. Mol. Model.* **2016**, *22*. https://doi.org/10.1007/s00894-016-3156-0.

(107) Zuriaga, E.; Giner, B.; Valero, M. S.; Gómez, M.; García, C. B.; Lomba, L. QSAR Modelling for Predicting the Toxic Effects of Traditional and Derived Biomass Solvents on a Danio Rerio Biomodel. *Chemosphere* **2019**, *227*, 480–488. https://doi.org/10.1016/j.chemosphere.2019.04.054.

(108) Roberts, J. F.; Marshall, S. J.; Roberts, D. W. Aquatic Toxicity of Ethoxylated and Propoxylated Alcohols to Daphnia Magna. *Environ. Toxicol. Chem.* **2007**, *26*, 68–72. https://doi.org/10.1897/07-023R.1.

(109) Lechuga, M.; Fernández-Serrano, M.; Jurado, E.; Núñez-Olea, J.; Ríos, F. Acute Toxicity of Anionic and Non-Ionic Surfactants to Aquatic Organisms. *Ecotoxicol. Environ. Saf.* **2016**, *125*, 1–8. https://doi.org/10.1016/j.ecoenv.2015.11.027.

(110) Liu, W.; Wang, X.; Zhou, X.; Duan, H.; Zhao, P.; Liu, W. Quantitative Structure-Activity

Relationship between the Toxicity of Amine Surfactant and Its Molecular Structure. *Sci. Total Environ.* **2020**, *702*, 134593. https://doi.org/10.1016/j.scitotenv.2019.134593.

(111) Hossain, K. A.; Roy, K. Chemometric Modeling of Aquatic Toxicity of Contaminants of Emerging Concern (CECs) in Dugesia Japonica and Its Interspecies Correlation with Daphnia and Fish: QSTR and QSTTR Approaches. *Ecotoxicol. Environ. Saf.* **2018**, *166*, 92–101. https://doi.org/10.1016/j.ecoenv.2018.09.068.

(112) Önlü, S.; Saçan, M. T. Toxicity of Contaminants of Emerging Concern to Dugesia Japonica: QSTR Modeling and Toxicity Relationship with Daphnia Magna. *J. Hazard. Mater.* **2018**, *351*, 20–28. https://doi.org/10.1016/j.jhazmat.2018.02.046.

(113) Ren, S.; Schultz, T. W. Identifying the Mechanism of Aquatic Toxicity of Selected Compounds by Hydrophobicity and Electrophilicity Descriptors. *Toxicol. Lett.* **2002**, *129*, 151–160. https://doi.org/10.1016/S0378-4274(01)00550-1.

(114) Michielan, L.; Pireddu, L.; Floris, M.; Moro, S. Support Vector Machine (SVM) as Alternative Tool to Assign Acute Aquatic Toxicity Warning Labels to Chemicals. *Mol. Inform.* **2010**, *29*, 51–64. https://doi.org/10.1002/minf.200900005.

(115) Lozano, S.; Halm-Lemeille, M. P.; Lepailleur, A.; Rault, S.; Bureau, R. Consensus QSAR Related to Global or MOA Models: Application to Acute Toxicity for Fish. *Mol. Inform.* **2010**, *29*, 803–813. https://doi.org/10.1002/minf.201000104.

(116) Su, L.; Fu, L.; He, J.; Qin, W.; Sheng, L.; Abraham, M. H.; Zhao, Y. H. Comparison of Tetrahymena Pyriformis Toxicity Based on Hydrophobicity, Polarity, Ionization and Reactivity of Class-Based Compounds. *SAR QSAR Environ. Res.* **2012**, *23*, 537–552.

https://doi.org/10.1080/1062936X.2012.666567.

(117) Carriger, J. F.; Martin, T. M.; Barron, M. G. A Bayesian Network Model for Predicting Aquatic Toxicity Mode of Action Using Two Dimensional Theoretical Molecular Descriptors. *Aquat. Toxicol.* **2016**, *180*, 11–24. https://doi.org/10.1016/j.aquatox.2016.09.006.

(118) Ren, Y. Y.; Zhou, L. C.; Yang, L.; Liu, P. Y.; Zhao, B. W.; Liu, H. X. Predicting the Aquatic Toxicity Mode of Action Using Logistic Regression and Linear Discriminant Analysis. *SAR QSAR Environ. Res.* **2016**, *27*, 721–746. https://doi.org/10.1080/1062936X.2016.1229691.

(119) Boone, K. S.; Di Toro, D. M. Target Site Model: Application of the Polyparameter Target Lipid Model to Predict Aquatic Organism Acute Toxicity for Various Modes of Action. *Environ. Toxicol. Chem.* **2019**, *38*, 222–239. https://doi.org/10.1002/etc.4278.

(120) Boone, K. S.; Di Toro, D. M. Target Site Model: Predicting Mode of Action and Aquatic Organism Acute Toxicity Using Abraham Parameters and Feature-weighted K-nearest Neighbors Classification. *Environ. Toxicol. Chem.* **2019**, *38*, 375–386. https://doi.org/10.1002/etc.4324.

(121) Takata, M.; Lin, B. Le; Xue, M.; Zushi, Y.; Terada, A.; Hosomi, M. Predicting the Acute Ecotoxicity of Chemical Substances by Machine Learning Using Graph Theory. *Chemosphere* **2020**, *238*, 124604. https://doi.org/10.1016/j.chemosphere.2019.124604.

(122) Wu, X.; Zhang, Q.; Hu, J. QSAR Study of the Acute Toxicity to Fathead Minnow Based on a Large Dataset. *SAR QSAR Environ. Res.* **2016**, *27*, 147–164. https://doi.org/10.1080/1062936X.2015.1137353.

(123) Papa, E.; Villa, F.; Gramatica, P. Statistically Validated QSARs, Based on Theoretical Descriptors, for Modeling Aquatic Toxicity of Organic Chemicals in Pimephales p Romelas (Fathead Minnow) . *J. Chem. Inf. Model.* **2005**, *45*, 1256–1266. https://doi.org/10.1021/ci050212l.

(124) Dearden, J. C.; Cronin, M. T. D.; Zhao, Y.-H.; Raevsky, O. A. QSAR Studies of Compounds Acting by Polar and Non-Polar Narcosis: An Examination of the Role of Polarisability and Hydrogen Bonding. *Quant. Struct. Relationships* **2000**, *19*, 3–9. https://doi.org/10.1002/(SICI)1521-3838(200002)19:1<3::AID-QSAR3>3.3.CO;2-E.

(125) Raevsky, O. A.; Dearden, J. C. Creation of Predictive Models of Aquatic Toxicity of Environmental Pollutants with Different Mechanisms of Action on the Basis of Molecular Similarity and Hybot Descriptors. *SAR QSAR Environ. Res.* **2004**, *15*, 433–448. https://doi.org/10.1080/10629360412331297498.

(126) Katritzky, A. R.; Tatham, D. B.; Maran, U. Theoretical Descriptors for the Correlation of Aquatic Toxicity of Environmental Pollutants by Quantitative Structure-Toxicity Relationships. *J. Chem. Inf. Comput. Sci.* **2001**, *41*, 1162–1176. https://doi.org/10.1021/ci010011r.

(127) Klopman, G.; Stuart, S. E. Multiple Computer-Automated Structure Evaluation Study of Aquatic Toxicity. III. Vibrio Fischeri. *Environ. Toxicol. Chem.* **2003**, *22*, 466–472. https://doi.org/10.1897/1551-5028(2003)022<0466:MCASES>2.0.CO;2.

(128) Dimitrov, S. D.; Mekenyana, O. G.; Sinks, G. D.; Schultz, T. W. Global Modeling of Narcotic Chemicals: Ciliate and Fish Toxicity. *J. Mol. Struct. THEOCHEM* **2003**, *622*, 63–70. https://doi.org/10.1016/S0166-1280(02)00618-8.

(129) Casalegno, M.; Benfenati, E.; Sello, G. An Automated Group Contribution Method in Predicting Aquatic Toxicity: The Diatomic Fragment Approach. *Chem. Res. Toxicol.* **2005**, *18*, 740–746. https://doi.org/10.1021/tx049665v.

(130) Hsieh, S.-H.; Hsu, C.-H.; Tsai, D.-Y.; Chen, C.-Y. QUANTITATIVE STRUCTURE–ACTIVITY RELATIONSHIPS FOR TOXICITY OF NONPOLAR NARCOTIC CHEMICALS TO PSEUDOKIRCHNERIELLA SUBCAPITATA. *Environ. Toxicol. Chem.* **2006**, *25*, 2920. https://doi.org/10.1897/06-127R.1.

(131) Raevsky, O. A.; Grigor'ev, V. Y.; Weber, E. E.; Dearden, J. C. Classification and Quantification of the Toxicity of Chemicals to Guppy, Fathead Minnow and Rainbow Trout: Part 1. Nonpolar Narcosis Mode of Action. *QSAR Comb. Sci.* **2008**, *27*, 1274–1281. https://doi.org/10.1002/qsar.200860014.

(132) Raevsky, O. A.; Grigor'ev, V. Y.; Dearden, J. C.; Weber, E. E. Classification and Quantification of the Toxicity of Chemicals to Guppy, Fathead Minnow, and Rainbow Trout. Part 2. Polar Narcosis Mode of Action. *QSAR Comb. Sci.* **2009**, *28*, 163–174. https://doi.org/10.1002/qsar.200860016.

(133) Kühne, R.; Ebert, R. U.; Vonderohe, P. C.; Ulrich, N.; Brack, W.; Schüürmann, G. Read-across Prediction of the Acute Toxicity of Organic Compounds toward the Water Flea Daphnia Magna. *Mol. Inform.* **2013**, *32*, 108–120. https://doi.org/10.1002/minf.201200085.

(134) Aruoja, V.; Moosus, M.; Kahru, A.; Sihtmäe, M.; Maran, U. Measurement of Baseline Toxicity and QSAR Analysis of 50 Non-Polar and 58 Polar Narcotic Chemicals for the Alga Pseudokirchneriella Subcapitata. *Chemosphere* **2014**, *96*, 23–32. https://doi.org/10.1016/j.chemosphere.2013.06.088.

(135) Lyakurwa, F. S.; Yang, X.; Li, X.; Qiao, X.; Chen, J. Development of in Silico Models for Predicting LSER Molecular Parameters and for Acute Toxicity Prediction to Fathead Minnow (Pimephales Promelas). *Chemosphere* **2014**, *108*, 17–25. https://doi.org/10.1016/j.chemosphere.2014.02.076.

(136) Austin, T.; Denoyelle, M.; Chaudry, A.; Stradling, S.; Eadsforth, C. European Chemicals Agency Dossier Submissions as an Experimental Data Source: Refinement of a Fish Toxicity Model for Predicting Acute LC50 Values. *Environ. Toxicol. Chem.* **2015**, *34*, 369–378. https://doi.org/10.1002/etc.2817.

(137) Zhu, D.; Li, T. T.; Zheng, S. S.; Yan, L. C.; Wang, Y.; Fan, L. Y.; Li, C.; Zhao, Y. H. Comparison of Modes of Action between Fish and Zebrafish Embryo Toxicity for Baseline, Less Inert, Reactive and Specifically-Acting Compounds. *Chemosphere* **2018**, *213*, 414–422. https://doi.org/10.1016/j.chemosphere.2018.09.072.

(138) Bakire, S.; Yang, X.; Ma, G.; Wei, X.; Yu, H.; Chen, J.; Lin, H. Developing Predictive Models for Toxicity of Organic Chemicals to Green Algae Based on Mode of Action. *Chemosphere* **2018**, *190*, 463–470. https://doi.org/10.1016/j.chemosphere.2017.10.028.

(139) de Morais e Silva, L.; Alves, M. F.; Scotti, L.; Lopes, W. S.; Scotti, M. T. Predictive Ecotoxicity of MoA 1 of Organic Chemicals Using in Silico Approaches. *Ecotoxicol. Environ. Saf.* **2018**, *153*, 151–159. https://doi.org/10.1016/j.ecoenv.2018.01.054.

(140) Zhang, S.; Wang, N.; Su, L.; Xu, X.; Li, C.; Qin, W.; Zhao, Y. MOA-Based Linear and Nonlinear QSAR Models for Predicting the Toxicity of Organic Chemicals to Vibrio Fischeri. *Environ. Sci. Pollut. Res.* **2020**, *27*, 9114–9125. https://doi.org/10.1007/s11356-019-06681-y.

(141) Aptula, A. O.; Roberts, D. W.; Cronin, M. T. D.; Schultz, T. W. Chemistry−Toxicity Relationships for the Effects of Di- and Trihydroxybenzenes to Tetrahymena Pyriformis. *Chem. Res. Toxicol.* **2005**, *18*, 844–854. https://doi.org/10.1021/tx049666n.

(142) Chen, C. Y.; Ko, C. W.; Lee, P. I. Toxicity of Substituted Anilines to Pseudokirchneriella Subcapitata and Quantitative Structure-Activity Relationship Analysis for Polar Narcotics. *Environ. Toxicol. Chem.* **2007**, *26*, 1158–1164. https://doi.org/10.1897/06-293R.1.

(143) Dimitrov, S. D.; Mekenyan, O. G.; Walker, J. D. Non-Linear Modeling of Bioconcentration Using Partition Coefficients for Narcotic Chemicals. *SAR QSAR Environ. Res.* **2002**, *13*, 177–184. https://doi.org/10.1080/10629360290002299.

(144) Ren, S.; Frymier, P. D. Modeling the Toxicity of Polar and Nonpolar Narcotic Compounds to Luminescent Bacterium Shk1. *Environ. Toxicol. Chem.* **2002**, *21*, 2649–2653. https://doi.org/10.1002/etc.5620211217.

(145) Escher, B. I.; Baumer, A.; Bittermann, K.; Henneberger, L.; König, M.; Kühnert, C.; Klüver, N. General Baseline Toxicity QSAR for Nonpolar, Polar and Ionisable Chemicals and Their Mixtures in the Bioluminescence Inhibition Assay with Aliivibrio Fischeri. *Environ. Sci. Process. Impacts* **2017**, *19*, 414–428. https://doi.org/10.1039/c6em00692b.

(146) Klüver, N.; Vogs, C.; Altenburger, R.; Escher, B. I.; Scholz, S. Development of a General Baseline Toxicity QSAR Model for the Fish Embryo Acute Toxicity Test. *Chemosphere* **2016**, *164*, 164–173. https://doi.org/10.1016/j.chemosphere.2016.08.079.

(147) Fogel, G. B.; Cheung, M. Derivation of Quantitative Structure-Toxicity Relationships for Ecotoxicological Effects of Organic Chemicals: Evolving Neural Networks and Evolving

Rules. *2005 IEEE Congr. Evol. Comput. IEEE CEC 2005. Proc.* **2005**, *1*, 274–281. https://doi.org/10.1109/cec.2005.1554695.

(148) Grigor'ev, V. Y.; Razdol'skii, A. N.; Zagrebin, A. O.; Tonkopii, V. D.; Raevskii, O. A. QSAR Classification Models of Acute Toxicity of Organic Compounds with Respect to Daphnia Magna. *Pharm. Chem. J.* **2014**, *48*, 242–245. https://doi.org/10.1007/s11094-014-1086-7.

(149) Gramatica, P.; Vighi, M.; Consolaro, F.; Todeschini, R.; Finizio, A.; Faust, M. QSAR Approach for the Selection of Congeneric Compounds with a Similar Toxicological Mode of Action. *Chemosphere* **2001**, *42*, 873–883. https://doi.org/10.1016/S0045-6535(00)00180-6.

(150) Schwöbel, J. A. H.; Madden, J. C.; Cronin, M. T. D. Application of a Computational Model for Michael Addition Reactivity in the Prediction of Toxicity to Tetrahymena Pyriformis. *Chemosphere* **2011**, *85*, 1066–1074. https://doi.org/10.1016/j.chemosphere.2011.07.037.

(151) Khan, K.; Roy, K.; Benfenati, E. Ecotoxicological QSAR Modeling of Endocrine Disruptor Chemicals. *J. Hazard. Mater.* **2019**, *369*, 707–718. https://doi.org/10.1016/j.jhazmat.2019.02.019.

(152) Faucon, J. C.; Bureau, R.; Faisant, J.; Briens, F.; Rault, S. Prediction of the Daphnia Acute Toxicity from Heterogeneous Data. *Chemosphere* **2001**, *44*, 407–422. https://doi.org/10.1016/S0045-6535(00)00301-5.

(153) Tao, S.; Xi, X.; Xu, F.; Li, B.; Cao, J.; Dawson, R. A Fragment Constant QSAR Model for Evaluating the EC50 Values of Organic Chemicals to Daphnia Magna. *Environ. Pollut.*

2002, *116*, 57–64. https://doi.org/10.1016/S0269-7491(01)00119-1.

(154) Gini, G.; Craciun, M. V.; König, C.; Benfenati, E. Combining Unsupervised and Supervised Artificial Neural Networks to Predict Aquatic Toxicity. *J. Chem. Inf. Comput. Sci.* **2004**, *44*, 1897–1902. https://doi.org/10.1021/ci0401219.

(155) Devillers, J. A New Strategy for Using Supervised Artificial Neural Networks in QSAR. *SAR QSAR Environ. Res.* **2005**, *16*, 433–442. https://doi.org/10.1080/10659360500320578.

(156) Amini, A.; Muggleton, S. H.; Lodhi, H.; Sternberg, M. J. E. A Novel Logic-Based Approach for Quantitative Toxicology Prediction. *J. Chem. Inf. Model.* **2007**, *47*, 998–1006. https://doi.org/10.1021/ci600223d.

(157) Bowen, K. R.; Flanagan, K. B.; Acree, W. E.; Abraham, M. H. Correlating Toxicities of Organic Compounds to Select Protozoa Using the Abraham Model. *Sci. Total Environ.* **2006**, *369*, 109–118. https://doi.org/10.1016/j.scitotenv.2006.05.008.

(158) Pavan, M.; Netzeva, T. I.; Worth, A. P. Validation of a QSAR Model for Acute Toxicity. *SAR QSAR Environ. Res.* **2006**, *17*, 147–171. https://doi.org/10.1080/10659360600636253.

(159) Xue, Y.; Li, H.; Ung, C. Y.; Yap, C. W.; Chen, Y. Z. Classification of a Diverse Set of Tetrahymena Pyriformis Toxicity Chemical Compounds from Molecular Descriptors by Statistical Learning Methods. *Chem. Res. Toxicol.* **2006**, *19*, 1030–1039. https://doi.org/10.1021/tx0600550.

(160) Zhu, H.; Tropsha, A.; Fourches, D.; Varnek, A.; Papa, E.; Gramatical, P.; Öberg, T.; Dao, P.; Cherkasov, A.; Tetko, I. V. Combinatorial QSAR Modeling of Chemical Toxicants Tested against Tetrahymena Pyriformis. *J. Chem. Inf. Model.* **2008**, *48*, 766–784.

https://doi.org/10.1021/ci700443v.

(161) Eroglu, E.; Palaz, S.; Oltulu, O.; Turkmen, H.; Ozaydin, C. Comparative QSTR Study Using Semi-Empirical and First Principle Methods Based Descriptors for Acute Toxicity of Diverse Organic Compounds to the Fathead Minnow. *Int. J. Mol. Sci.* **2007**, *8*, 1265–1283. https://doi.org/10.3390/ijms8121265.

(162) Bašic, I.; Lučié, B.; Nikolić, S.; Papeš-Šokčević, L.; Nadramija, D. Improvement of Ensemble of Multi-Regression Structure-Toxicity Models by Clustering of Molecules in Descriptor Space. *AIP Conf. Proc.* **2009**, *1148 2*, 408–411. https://doi.org/10.1063/1.3225331.

(163) Moosus, M.; Maran, U. Quantitative Structure-Activity Relationship Analysis of Acute Toxicity of Diverse Chemicals to Daphnia Magna with Whole Molecule Descriptors. *SAR QSAR Environ. Res.* **2011**, *22*, 757–774. https://doi.org/10.1080/1062936X.2011.623317.

(164) In, Y.; Lee, S. K.; Kim, P. J.; No, K. T. Prediction of Acute Toxicity to Fathead Minnow by Local Model Based QSAR and Global QSAR Approaches. *Bull. Korean Chem. Soc.* **2012**, *33*, 613–619. https://doi.org/10.5012/bkcs.2012.33.2.613.

(165) Roy, K.; Das, R. N. QSTR with Extended Topochemical Atom (ETA) Indices. 15. Development of Predictive Models for Toxicity of Organic Chemicals against Fathead Minnow Using Second-Generation ETA Indices. *SAR QSAR Environ. Res.* **2012**, *23*, 125–140. https://doi.org/10.1080/1062936X.2011.645872.

(166) Singh, K. P.; Gupta, S.; Rai, P. Predicting Acute Aquatic Toxicity of Structurally Diverse Chemicals in Fish Using Artificial Intelligence Approaches. *Ecotoxicol. Environ. Saf.* **2013**,

95, 221–233. https://doi.org/10.1016/j.ecoenv.2013.05.017.

(167) Singh, K. P.; Gupta, S.; Kumar, A.; Mohan, D. Multispecies QSAR Modeling for Predicting the Aquatic Toxicity of Diverse Organic Chemicals for Regulatory Toxicology. *Chem. Res. Toxicol.* **2014**, *27*, 741–753. https://doi.org/10.1021/tx400371w.

(168) Singh, K. P.; Gupta, S. In Silico Prediction of Toxicity of Non-Congeneric Industrial Chemicals Using Ensemble Learning Based Modeling Approaches. *Toxicol. Appl. Pharmacol.* **2014**, *275*, 198–212. https://doi.org/10.1016/j.taap.2014.01.006.

(169) Tugcu, G.; Yilmaz, H. B.; Türker Saçan, M. Comparative Performance of Descriptors in a Multiple Linear and Kriging Models: A Case Study on the Acute Toxicity of Organic Chemicals to Algae. *Environ. Sci. Pollut. Res.* **2014**, *21*, 11924–11932. https://doi.org/10.1007/s11356-014-3182-3.

(170) Wang, Q.; Jia, Q.; Yan, L.; Xia, S.; Ma, P. Quantitative Structure-Toxicity Relationship of the Aquatic Toxicity for Various Narcotic Pollutants Using the Norm Indexes. *Chemosphere* **2014**, *108*, 383–387. https://doi.org/10.1016/j.chemosphere.2014.02.030.

(171) Cassotti, M.; Ballabio, D.; Todeschini, R.; Consonni, V. A Similarity-Based QSAR Model for Predicting Acute Toxicity towards the Fathead Minnow (Pimephales Promelas). *SAR QSAR Environ. Res.* **2015**, *26*, 217–243. https://doi.org/10.1080/1062936X.2015.1018938.

(172) Vikas, R. Exploring the Role of Quantum Chemical Descriptors in Modeling Acute Toxicity of Diverse Chemicals to Daphnia Magna. *J. Mol. Graph. Model.* **2015**, *61*, 89–101. https://doi.org/10.1016/j.jmgm.2015.06.009.

(173) Martin, T. M.; Young, D. M.; Lilavois, C. R.; Barron, M. G. Comparison of Global and

Mode of Action-Based Models for Aquatic Toxicity. *SAR QSAR Environ. Res.* **2015**, *26*, 245–262. https://doi.org/10.1080/1062936X.2015.1018939.

(174) Aalizadeh, R.; von der Ohe, P. C.; Thomaidis, N. S. Prediction of Acute Toxicity of Emerging Contaminants on the Water Flea Daphnia Magna by Ant Colony Optimization–Support Vector Machine QSTR Models. *Environ. Sci. Process. Impacts* **2017**, *19*, 438–448. https://doi.org/10.1039/C6EM00679E.

(175) Alves, V. M.; Golbraikh, A.; Capuzzi, S. J.; Liu, K.; Lam, W. I.; Korn, D. R.; Pozefsky, D.; Andrade, C. H.; Muratov, E. N.; Tropsha, A. Multi-Descriptor Read Across (MuDRA): A Simple and Transparent Approach for Developing Accurate Quantitative Structure-Activity Relationship Models. *J. Chem. Inf. Model.* **2018**, *58*, 1214–1223. https://doi.org/10.1021/acs.jcim.8b00124.

(176) Cao, Q.; Liu, L.; Yang, H.; Cai, Y.; Li, W.; Liu, G.; Lee, P. W.; Tang, Y. In Silico Estimation of Chemical Aquatic Toxicity on Crustaceans Using Chemical Category Methods. *Environ. Sci. Process. Impacts* **2018**, *20*, 1234–1243. https://doi.org/10.1039/c8em00220g.

(177) Jia, Q.; Zhao, Y.; Yan, F.; Wang, Q. QSAR Model for Predicting the Toxicity of Organic Compounds to Fathead Minnow. *Environ. Sci. Pollut. Res.* **2018**, *25*, 35420–35428. https://doi.org/10.1007/s11356-018-3434-8.

(178) Vazhev; Munarbaeva; Yergaliyeva; Vazheva; Gubenko. Modeling of Acute Aqueous Toxicity of Organic Compounds for Daphnia Magna. *Bull. Karaganda Univ. “Chemistry” Ser.* **2018**, *90*, 81–85. https://doi.org/10.31489/2018Ch2/81-85.

(179) Ai, H.; Wu, X.; Zhang, L.; Qi, M.; Zhao, Y.; Zhao, Q.; Zhao, J.; Liu, H. QSAR Modelling

Study of the Bioconcentration Factor and Toxicity of Organic Compounds to Aquatic Organisms Using Machine Learning and Ensemble Methods. *Ecotoxicol. Environ. Saf.* **2019**, *179*, 71–78. https://doi.org/10.1016/j.ecoenv.2019.04.035.

(180) Jiang, C.; Yang, H.; Di, P.; Li, W.; Tang, Y.; Liu, G. In Silico Prediction of Chemical Reproductive Toxicity Using Machine Learning. *J. Appl. Toxicol.* **2019**, *39*, 844–854. https://doi.org/10.1002/jat.3772.

(181) Matveieva, M.; Cronin, M. T. D.; Polishchuk, P. Interpretation of QSAR Models: Mining Structural Patterns Taking into Account Molecular Context. *Mol. Inform.* **2019**, *38*. https://doi.org/10.1002/minf.201800084.

(182) Sheffield, T. Y.; Judson, R. S. Ensemble QSAR Modeling to Predict Multispecies Fish Toxicity Lethal Concentrations and Points of Departure. *Environ. Sci. Technol.* **2019**, *53*, 12793–12802. https://doi.org/10.1021/acs.est.9b03957.

(183) Toropov, A. A.; Toropova, A. P.; Benfenati, E. The Index of Ideality of Correlation: QSAR Model of Acute Toxicity for Zebrafish (Danio Rerio) Embryo. *Int. J. Environ. Res.* **2019**, *13*, 387–394. https://doi.org/10.1007/s41742-019-00183-y.

(184) Liu, T.; Yan, F.; Jia, Q.; Wang, Q. Norm Index-Based QSAR Models for Acute Toxicity of Organic Compounds toward Zebrafish Embryo. *Ecotoxicol. Environ. Saf.* **2020**, *203*, 110946. https://doi.org/10.1016/j.ecoenv.2020.110946.

(185) Lunghini, F.; Marcou, G.; Azam, P.; Enrici, M. H.; Van Miert, E.; Varnek, A. Consensus QSAR Models Estimating Acute Toxicity to Aquatic Organisms from Different Trophic Levels: Algae, Daphnia and Fish. *SAR QSAR Environ. Res.* **2020**, *31*, 655–675.

https://doi.org/10.1080/1062936X.2020.1797872.

(186) Tinkov, O.; Polishchuk, P.; Matveieva, M.; Grigorev, V.; Grigoreva, L.; Porozov, Y. The Influence of Structural Patterns on Acute Aquatic Toxicity of Organic Compounds. *Mol. Inform.* **2020**, *2000209*, 1–14. https://doi.org/10.1002/minf.202000209.

(187) Wang, Y.; Chen, X. A Joint Optimization QSAR Model of Fathead Minnow Acute Toxicity Based on a Radial Basis Function Neural Network and Its Consensus Modeling. *RSC Adv.* **2020**, *10*, 21292–21308. https://doi.org/10.1039/D0RA02701D.

(188) Yu, X. Quantitative Structure-Toxicity Relationships of Organic Chemicals against Pseudokirchneriella Subcapitata. *Aquat. Toxicol.* **2020**, *224*, 105496. https://doi.org/10.1016/j.aquatox.2020.105496.

(189) Yu, X. Prediction of Chemical Toxicity to Tetrahymena Pyriformis with Four-Descriptor Models. *Ecotoxicol. Environ. Saf.* **2020**, *190*, 110146. https://doi.org/10.1016/j.ecoenv.2019.110146.

(190) Dimitrov, S.; Koleva, Y.; Schultz, T. W.; Walker, J. D.; Mekenyan, O. Interspecies Quantitative Structure-Activity Relationship Model for Aldehydes: Aquatic Toxicity. *Environ. Toxicol. Chem.* **2004**, *23*, 463–470. https://doi.org/10.1897/02-579.

(191) Kahn, I.; Maran, U.; Benfenati, E.; Netzeva, T. I.; Schultz, T. W.; Cronin, M. T. D. Comparative Quantitative Structure-Activity-Activity Relationships for Toxicity to Tetrahymena Pyriformis and Pimephales Prometas. *ATLA Altern. to Lab. Anim.* **2007**, *35*, 15–24. https://doi.org/10.1177/026119290703500112.

(192) Zhang, X. J.; Qin, H. W.; Su, L. M.; Qin, W. C.; Zou, M. Y.; Sheng, L. X.; Zhao, Y. H.;

Abraham, M. H. Interspecies Correlations of Toxicity to Eight Aquatic Organisms: Theoretical Considerations. *Sci. Total Environ.* **2010**, *408*, 4549–4555. https://doi.org/10.1016/j.scitotenv.2010.07.022.

(193) Furuhama, A.; Hasunuma, K.; Hayashi, T. I.; Tatarazako, N. Predicting Algal Growth Inhibition Toxicity: Three-Step Strategy Using Structural and Physicochemical Properties. *SAR QSAR Environ. Res.* **2016**, *27*, 343–362. https://doi.org/10.1080/1062936X.2016.1174151.

(194) Zolotarev, K. V.; Belyaeva, N. F.; Mikhailov, A. N.; Mikhailova, M. V. Dependence between LD50 for Rodents and LC50 for Adult Fish and Fish Embryos. *Bull. Exp. Biol. Med.* **2017**, *162*, 445–450. https://doi.org/10.1007/s10517-017-3636-y.

(195) Li, J. J.; Zhang, X. J.; Yang, Y.; Huang, T.; Li, C.; Su, L.; Zhao, Y. H.; Cronin, M. T. D. Development of Thresholds of Excess Toxicity for Environmental Species and Their Application to Identification of Modes of Acute Toxic Action. *Sci. Total Environ.* **2018**, *616–617*, 491–499. https://doi.org/10.1016/j.scitotenv.2017.10.308.

(196) Bouhedjar, K.; Benfenati, E.; Nacereddine, A. K. Modelling Quantitative Structure Activity–Activity Relationships (QSAARs): Auto-Pass-Pass, a New Approach to Fill Data Gaps in Environmental Risk Assessment under the REACH Regulation. *SAR QSAR Environ. Res.* **2020**, *31*, 785–801. https://doi.org/10.1080/1062936X.2020.1810770.

(197) Schultz, T. W.; Yarbrough, J. W.; Pilkington, T. B. Aquatic Toxicity and Abiotic Thiol Reactivity of Aliphatic Isothiocyanates: Effects of Alkyl-Size and -Shape. *Environ. Toxicol. Pharmacol.* **2007**, *23*, 10–17. https://doi.org/10.1016/j.etap.2006.05.005.

(198) Schultz, T. W.; Ralston, K. E.; Roberts, D. W.; Veith, G. D.; Aptula, A. O. Structure-Activity Relationships for Abiotic Thiol Reactivity and Aquatic Toxicity of Halo-Substituted Carbonyl Compounds. *SAR QSAR Environ. Res.* **2007**, *18*, 21–29. https://doi.org/10.1080/10629360601033424.

(199) Furuhama, A.; Hasunuma, K.; Aoki, Y. Interspecies Quantitative Structure–Activity–Activity Relationships (QSAARs) for Prediction of Acute Aquatic Toxicity of Aromatic Amines and Phenols. *SAR QSAR Environ. Res.* **2015**, *26*, 301–323. https://doi.org/10.1080/1062936X.2015.1032347.

(200) Florescu, C.; Caragea, C. PositionRank: An Unsupervised Approach to Keyphrase Extraction from Scholarly Documents. *ACL 2017 - 55th Annu. Meet. Assoc. Comput. Linguist. Proc. Conf. (Long Pap.* **2017**, *1*, 1105–1115. https://doi.org/10.18653/v1/P17-1102.

(201) Kim, S. N.; Medelyan, O.; Kan, M. Y.; Baldwin, T. Automatic Keyphrase Extraction from Scientific Articles. *Lang. Resour. Eval.* **2013**, *47*, 723–742. https://doi.org/10.1007/s10579-012-9210-3.

(202) Campos, R.; Mangaravite, V.; Pasquali, A.; Jorge, A.; Nunes, C.; Jatowt, A. YAKE! Keyword Extraction from Single Documents Using Multiple Local Features. *Inf. Sci. (Ny).* **2020**, *509*, 257–289. https://doi.org/10.1016/j.ins.2019.09.013.

(203) Bougouin, A.; Boudin, F. TopicRank: Topic Ranking for Automatic Keyphrase Extraction. *Rev. Trait. Autom. des Langues* **2014**, *55*, 45–69.

(204) Boudin, F. Unsupervised Keyphrase Extraction with Multipartite Graphs. *NAACL HLT*

2018 - 2018 Conf. North Am. Chapter Assoc. Comput. Linguist. Hum. Lang. Technol. - Proc. Conf. **2018**, *2*, 667–672. https://doi.org/10.18653/v1/n18-2105.

(205) Nguyen, T. D.; Luong, M. T. WINGNUS: Key Phrase Extraction Utilizing Document Logical Structure. *ACL 2010 - SemEval 2010 - 5th Int. Work. Semant. Eval. Proc.* **2010**, No. July, 166–169.

(206) Manzetti, S. Ecotoxicity of Polycyclic Aromatic Hydrocarbons, Aromatic Amines, and Nitroarenes through Molecular Properties. *Environ. Chem. Lett.* **2012**, *10*, 349–361. https://doi.org/10.1007/s10311-012-0368-0.

(207) Belanger, S. E.; Sanderson, H.; Fisk, P. R.; Schäfers, C.; Mudge, S. M.; Willing, A.; Kasai, Y.; Nielsen, A. M.; Dyer, S. D.; Toy, R. Assessment of the Environmental Risk of Long-Chain Aliphatic Alcohols. *Ecotoxicol. Environ. Saf.* **2009**, *72*, 1006–1015. https://doi.org/10.1016/j.ecoenv.2008.07.013.

(208) Wei, B.; Sun, J.; Mei, Q.; An, Z.; Wang, X.; He, M. Theoretical Study on Gas-Phase Reactions of Nitrate Radicals with Methoxyphenols: Mechanism, Kinetic and Toxicity Assessment. *Environ. Pollut.* **2018**, *243*, 1772–1780. https://doi.org/10.1016/j.envpol.2018.08.104.

(209) Vita, N. A.; Brohem, C. A.; Canavez, A. D. P. M.; Oliveira, C. F. S.; Kruger, O.; Lorencini, M.; Carvalho, C. M. Parameters for Assessing the Aquatic Environmental Impact of Cosmetic Products. *Toxicol. Lett.* **2018**, *287*, 70–82. https://doi.org/10.1016/j.toxlet.2018.01.015.

(210) Melnikov, F.; Kostal, J.; Voutchkova-Kostal, A.; Zimmerman, J. B.; Anastas, P. T.

Assessment of Predictive Models for Estimating the Acute Aquatic Toxicity of Organic Chemicals. *Green Chem.* **2016**, *18*, 4432–4445. https://doi.org/10.1039/c6gc00720a.

(211) Abe, T.; Saito, H.; Niikura, Y.; Shigeoka, T.; Nakano, Y. Embryonic Development Assay with Daphnia Magna: Application to Toxicity of Aniline Derivatives. *Chemosphere* **2001**, *45*, 487–495. https://doi.org/10.1016/S0045-6535(01)00049-2.

(212) Ahlers, J.; Nendza, M.; Schwartz, D. Environmental Hazard and Risk Assessment of Thiochemicals. Application of Integrated Testing and Intelligent Assessment Strategies (ITS) to Fulfil the REACH Requirements for Aquatic Toxicity. *Chemosphere* **2019**, *214*, 480–490. https://doi.org/10.1016/j.chemosphere.2018.09.082.

(213) Cassotti, M.; Consonni, V.; Mauri, A.; Ballabio, D. Validation and Extension of a Similarity-Based Approach for Prediction of Acute Aquatic Toxicity towards Daphnia Magna. *SAR QSAR Environ. Res.* **2014**, *25*, 1013–1036. https://doi.org/10.1080/1062936X.2014.977818.

(214) Bajusz, D.; Rácz, A.; Héberger, K. Why Is Tanimoto Index an Appropriate Choice for Fingerprint-Based Similarity Calculations? *J. Cheminform.* **2015**, *7*, 20. https://doi.org/10.1186/s13321-015-0069-3.

(215) Landrum, G. *RDKit Documentation. Release 2017.03.1*; 2017. https://doi.org/10.5281/zenodo.60510.

(216) Kessler, T. PaDELPy: A Python Wrapper for PaDEL-Descriptor Software. 2021.

(217) Lu, J.; Zhang, P.; Zou, X. W.; Zhao, X. Q.; Cheng, K. G.; Zhao, Y. L.; Bi, Y.; Zheng, M. Y.; Luo, X. M. In Silico Prediction of Chemical Toxicity Profile Using Local Lazy

Learning. *Comb. Chem. High Throughput Screen.* **2017**, *20*, 346–353. https://doi.org/10.2174/1386207320666170217151826.

(218) Shavalieva, G.; Papadopoulos, A. I.; Badr, S.; Seferlis, P.; Papadokonstantakis, S. Sustainability Assessment Using Local Lazy Learning: The Case of Post-Combustion CO2 Capture Solvents. In *13th International Symposium on Process Systems Engineering (PSE 2018)*; 2018; pp 823–828. https://doi.org/10.1016/B978-0-444-64241-7.50132-4.

(219) ECHA. *Guidance on Information Requirements and Chemical Safety Assessment*; 2017. https://doi.org/10.2823/128621.
